# Comparative 3-D Modeling of tmRNA

**DOI:** 10.1186/1471-2199-6-14

**Published:** 2005-06-15

**Authors:** Jody Burks, Christian Zwieb, Florian Müller, Iwona Wower, Jacek Wower

**Affiliations:** 1 Department of Animal Sciences, Cellular and Molecular Biosciences Program, Auburn University, Auburn, AL 36849 USA; 2 Department of Molecular Biology, University of Texas Health Science Center at Tyler, 11937 US Hwy 271, Tyler, 75708 TX USA; 3 Max Planck Institute for Molecular Genetics, Ihnestrasse 73, D-14195, Berlin, Germany

## Abstract

**Background:**

*Trans-*translation releases stalled ribosomes from truncated mRNAs and tags defective proteins for proteolytic degradation using transfer-messenger RNA (tmRNA). This small stable RNA represents a hybrid of tRNA- and mRNA-like domains connected by a variable number of pseudoknots. Comparative sequence analysis of tmRNAs found in bacteria, plastids, and mitochondria provides considerable insights into their secondary structures. Progress toward understanding the molecular mechanism of template switching, which constitutes an essential step in *trans-*translation, is hampered by our limited knowledge about the three-dimensional folding of tmRNA.

**Results:**

To facilitate experimental testing of the molecular intricacies of *trans-*translation, which often require appropriately modified tmRNA derivatives, we developed a procedure for building three-dimensional models of tmRNA. Using comparative sequence analysis, phylogenetically-supported 2-D structures were obtained to serve as input for the program ERNA-3D. Motifs containing loops and turns were extracted from the known structures of other RNAs and used to improve the tmRNA models. Biologically feasible 3-D models for the entire tmRNA molecule could be obtained. The models were characterized by a functionally significant close proximity between the tRNA-like domain and the resume codon. Potential conformational changes which might lead to a more open structure of tmRNA upon binding to the ribosome are discussed. The method, described in detail for the tmRNAs of *Escherichia coli*, *Bacillus anthracis*, and *Caulobacter crescentus*, is applicable to every tmRNA.

**Conclusion:**

Improved molecular models of biological significance were obtained. These models will guide in the design of experiments and provide a better understanding of *trans-*translation. The comparative procedure described here for tmRNA is easily adopted for the modeling the members of other RNA families.

## Background

Transfer-messenger RNA (tmRNA), also known as 10Sa RNA or ssrA RNA, is a hybrid of a tRNA-like domain (TLD) and a mRNA-like domain (MLD) connected by a variable number of pseudoknots [1]. TmRNA is a stable and essential component of *trans-*translation, a quality-control process that rescues ribosomes stalled on mRNAs lacking stop codons. During *trans-*translation, ribosomes switch from a defective mRNA (lacking its translation-termination signal) to the MLD of tmRNA. Because a stop codon is provided by the tmRNA, the ribosomes can dissociate and recycle [2]. As an additional advantage, the tandem translation of the two templates generates a tagged polypeptide which is degraded by housekeeping proteases [3,4].

For tagging, tmRNA has to be charged by aminoacyl-tRNA synthetases [5]. Assisted by protein SmpB, the charged tmRNA is delivered to stalled ribosomes as a quaternary complex with EF-Tu and GTP. Binding of tmRNA to ribosomes is facilitated by ribosomal protein S1, which interacts with the MLD and pseudoknots but not with the TLD [6-9]. Recently, cryo-EM revealed the shape of the tmRNA associated with SmpB and EF-Tu in its ribosome-bound form [10]. Despite this significant progress, high-resolution structures as obtained by NMR and X-ray crystallography are unavailable and expected to be difficult to obtain in the foreseeable future due to the relatively large size and flexibility of the tmRNA.

In the present work we used a stepwise procedure for arriving at high-resolution models for the entire tmRNA molecule. First, 2-D structures were obtained by covariation analysis of a large number of tmRNA sequences. The basepairing information was submitted to the ERNA-3D modeling program [11] to build the helical sections. Structural motifs of the loops and turns were identified in SCOR [12], high-resolution data were extracted from known structures, and these data were incorporated into the models. Overall, significantly improved 3-D models were obtained which will be useful to understand the role of tmRNA in *trans*-translation. The described approach can be adapted to obtain high-resolution models of the members of other RNA families.

## Results

### Identification of tmRNA sequences

The tmRNA sequences were identified previously and subjected to comparative sequence analysis (CSA) as described [1,13]. New tmRNA sequences were obtained from the tmRNA website [14], through keyword searches of the literature and GenBank [15], or BLAST [16,17], and various genome sequencing projects. The new sequences were examined iteratively as described in Materials and Methods to confirm tmRNA identity, remove sequence duplications, and create a meaningful alignment.

New potential tmRNA sequences were maintained as a preliminary alignment in BioEdit [18], separate EMBL-formatted sequence files, and a HTML-formatted phylogenetic list. The sequences were ordered phylogenetically using the information in the Ribosomal Database Project (RDP) [19]. If the organism name was not listed in the RDP, the sequence was placed next to its closest relative using the NCBI Taxonomy resource [20].

### Selection of tmRNA sequences

The new sequences were confirmed individually as tmRNAs by comparison with the closest relative using the pairwise alignment feature of BioEdit [18]. If there was a lack of obvious similarity, the sequence was inspected for evidence of biological features such as the ability to form a TLD and an open reading frame. Furthermore, the possibility of a two-part tmRNA was considered. A sequence suspected to be a new tmRNA was investigated further by CSA [13] as described in Materials and Methods.

Potential new sequences of the alpha-Proteobacteria and some Cyanobacteria that were encoded in two separate sections of their genes [21], were compared to the two-part tmRNA sequence from a closest relative for effective comparison with the one-part tmRNAs. The 3'- and 5'-ends of each section were determined by pairwise alignment to generate a single sequence. Each of the 20 new two-part tmRNAs (14 sequences from alpha-Proteobacteria and six from Cyanobacteria) was subjected to this rearrangement.

### Comparative Sequence Analysis

Sequences were ordered phylogenetically using the RDP [19] as a guide or by alignment with the closest relative. Identical regions were aligned first. Subsequently, similar regions were aligned using invariant positions as signposts. Regions of biological significance, such as the resume and stop codons, were then considered. Finally, common secondary structure features were used to align regions that lacked primary structure similarity or biological features. Supported Watson-Crick basepairs and G-U interactions were indicated in the alignment by uppercase letters. Gaps were introduced to account for differences in sequence length and to avoid the alignment of dissimilar regions.

Secondary structure was determined using covariation analysis as described [13] (see also Materials and Methods). The alignment was examined to identify compensatory base changes (CBCs) and other covariations. The numbers of CBCs and mismatches between the alignment columns were counted. CBCs provided positive evidence for the existence of a basepair; mismatches provided negative evidence. If the number of compensatory base changes was two times or greater than the number of mismatches, the basepair was considered supported. If a basepair was invariant, no evidence for or against its existence could be gained from CSA. A basepair was considered specific to a particular phylogenetic group if it was proven only in that group.

### Quality control

To check for the proper assignment of basepairs, the alignment was sent through an automated pipeline of programs from RNAdbTools [22]. The output was inspected visually and corrections were made manually using the BioEdit program [18]. The revised alignment was resubmitted to RNAdbTools, and the review process was repeated until a satisfactory alignment was produced.

### TmRNA alignment

The final alignment contained a total of 274 tmRNA sequences in 16 bacterial phylogenetic groups. A complete phylogenetic list is available at the tmRDB [23]. There was a substantial increase in the number of two-part tmRNAs for a total of 27 sequences: 20 from alpha-Proteobacteria (20 tmRNAs), one mitochondrial tmRNA, and six cyanobacterial tmRNAs. The nine organelle sequences included one from a cyanelle, six from chloroplasts, one from a plastid, and one from the *Reclinomonas americana *mitochondrion. The typical tmRNA was about 350 nucleotides long. The *R. americana *mitochondrion tmRNA contained only 189 nucleotides and, since it appeared to lack an ORF, may not be functional. Excluding this exception and any partial tmRNAs, the tmRNA of *Synechococcus *species PCC7009 was the shortest (250 nucleotides), and the longest was from *Chlamydophila psittaci *(425 nucleotides). The tmRNA alignment is provided as additional files 1: tmRNA-alignment.html, 2: tmRNA-alignment-wide.txt, 3:tmRNA-alignment-92col.txt, and 4: tmRNA-alignment.msf.

### Secondary structure of tmRNA

**Figure 1:**
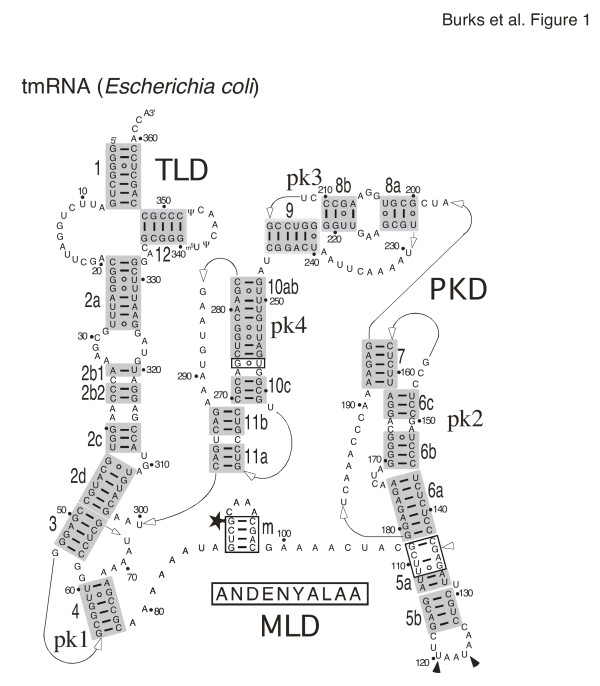
**Secondary structure of *E. coli *tmRNA**. Phylogenetically-supported helices are highlighted in gray and numbered from 1 to 12. The 5' and 3' ends are indicated. Arrows represent connections from 5' to 3'. Residues are numbered in increments of ten. Weakly supported regions and basepairs are shown in boxes. The disproved potential pairing of C109 with G136 is labeled with an open arrowhead. The star labels the first nucleotide of the resume codon. The tag peptide sequence is shown below the mRNA-like region. The stop codons are indicated with solid arrowheads. Three domains are distinguished: the tRNA-like domain (TLD), the mRNA-like domain (MLD), and the pseudoknot domain (PKD).

The tmRNA secondary structure features were extracted from the alignment and are listed in phylogenetic order in Table 1. The representative secondary structure of *Escherichia coli *tmRNA is shown in Figure 1. Secondary structures of *Bacillus anthracis *and *Caulobacter crescentus *are presented in as additional file 5: Banthracis2D.pdf and additional file 6: Ccrescentus2D.pdf, respectively.

### TLD (helices 1, 2a and 12)

Although a prominent feature of each tmRNA, the TLD was relatively weakly supported by CSA due to a high degree of sequence conservation. However, the structure of this region is well established by experimental evidence [24-26].

Helix 1 contained seven basepairs and was usually continuous with the exception of the *Anabaena *species tmRNA, which contained an insertion in the 3'-portion of helix 1. The first pair (1G-C359 in *E. coli *tmRNA) was conserved with one exception in *Alcaligenes eutrophus *where there was a 1U-C345 mismatch possibly due to a sequencing error. The second (2G-C358,*E. coli *numbering, Figure 1) and third pair (3G-U357) of helix 1 were invariant and therefore neither supported nor disproved by CSA. The identities of the bases involved in the fourth (4G-C356) and fifth pair varied. The closing pair of helix 1 (7G-C353) was conserved with the exception of a 7U-A388 pair the *Trichodesmium erythraeum *tmRNA. The single-stranded region between helices 1 and 2a ranged from ten in *Dehalococcoides ethenogenes *to 13 nucleotides in one *Clostridium acetobutylicum *sequence. A U-A basepair (U6 in chain A with A88 in chain B) was possible in the *R. americana *mitochondrion tmRNA.

Helix 2a was equivalent to the anticodon stem of tRNA and contained eight supported basepairs as well as a short variable internal loop in the 5'half of the helix that occurred in a few sequences (e.g. *Caulobacter crescentus*, see additional file 6: Ccrescentus2D.pdf). The first position in the helix was a conserved cytosine (C21 in *E. coli*) which formed a weakly-supported basepair with the conserved G333. The partial tmRNA from the chloroplast of *Pavlova lutheri *contained a uracil in the first position, but no information regarding the 3'portion of helix 2a was available.

The T-loop and helix 12 were highly conserved, although many sequences lacked information about helix 12 due to primer annealing during PCR amplification. Helix 12 contained four strongly supported basepairs and a fifth conserved G-C pair (340G-C348 in *E. coli*; Figure 1). The *Dehalococcoides ethenogenes *tmRNA had the potential to form a sixth basepair in helix 12. Helix 12 was almost always continuous, except for the tmRNA of *Carboxydothermus hydrogenoformans *which possessed four basepairs and a mismatched U333 and C347. A 331-GG-332 preceded U333 in *C. hydrogenoformans *and followed the conserved 328-GAC-330. Therefore, U333 was unlikely to pair. In the T-loop, the U341 and U342 (*E. coli *tmRNA) seen in most sequences were replaced by two guanines in the tmRNA from the *R. americana *mitochondrion (G79 and G80 in chainB) [21]. In the tmRNA from *Caulobacter crescentus*, the nucleotide corresponding to U342 in *E. coli *tmRNA was changed to G62 in chainB (see additional file 6: Ccrescentus2D.pdf).

### Helical sections 2b, 2c and 2d

Overall, sections 2b, 2c, and 2d were well supported. Sections 2a and 2b were separated by a variable loop ranging from one to seven nucleotides in the 5'portion and from one to nine nucleotides in the 3'portion. Sections 2b and 2c had the potential to form a continuously stacked helix (e.g. in *Chlamydophila psittaci *tmRNA). Usually, a bulge of two to six nucleotides separated helical sections 2c and 2d (residues 309–311 in *E. coli *tmRNA, Figure 1). An asymmetrical loop was present in some sequences (for example, residues 40–41 in chainA, and 27–31 in chainB of *Caulobacter crescentus *tmRNA, see additional file 6: Ccrescentus2D.pdf). Helix 2d was the most conserved of the three helical sections. The G43-U308 basepair (*E. coli *numbering) in helix 2d was only weakly supported, conserved in most phylogenetic groups, but altered in the Thermatogales, Cyanobacteria, alpha-Proteobacteria, and Gram-positive bacteria. A 46A-U334 pair was possible in the *Synechocystis *species PCC6803 tmRNA.

### Pseudoknot 1 (helices 3 and 4)

Pseudoknot 1 (pk1) was well supported. Of the three connecting regions, the two 5'-regions were very short (no or only one residue) while the third was relatively long (one to 11 residues). All pseudoknots in tmRNA followed the same general design [27]. Most sequences contained helices 3 and 4, with the exception of the tmRNA from *Oenococcus oeni *and the partial sequence from the chloroplast of *Pavlova lutheri*, both of which lacked helix 4 and thus did not form a pseudoknot. Helix 3 usually contained five basepairs. However, a sixth pair was possible in some bacteria.

Helix 4 could be split into helicalsections 4a and 4b by a bulge seen in 46 sequences (position57 in *B. anthracis *tmRNA; see additional file 5: Banthracis2D.pdf) or an internal loop seen in 52 tmRNA sequences. The adenine-rich terminal loop between the downstream halves of helices 3 and 4 ranged in length from twoto13 nucleotides.

### The mRNA-like region (MLD)

The MLD consisted of an open reading frame (ORF) preceding helix 5 and varied from 48 (*Heliobacillus mobilis*) to 126 nucleotides (*Odontella sinensis *chloroplast). The resume codon usually coded for alanine, but for glycine in 30 sequences (e.g. *Bacillus anthracis*), aspartic acid in three sequences (e.g. *Staphylococcus epidermidis*), arginine in two uncultured species (FS1 and LEM2), serine in the uncultured species RCA1, and glutamic acid in *Mycoplasma pulmonis*.

Although helix 5 was only weakly supported by CBCs, recent site-directed mutagenesis experiments combined with functional studies *in vivo *and *in vitro *[28] provide strong evidence for its existence. One to three stop codons were located within the helix 5 loop. A single UAA stop codon was present in 157 sequences. UAG (17 sequences) or UGA (10 sequences) were used less frequently. In 85 sequences there were two in-frame stop codons, where UAA was always the first codon, followed by another UAA (73 sequences), UAG (10 sequences) or UGA (2 sequences). Curiously, two sequences (*Bacillus megaterium *and *Chloroflexus aurantiacu*) were found to contain three tandem in-frame stop codons.

### Pseudoknot 2 (helices 6 and 7)

Pseudoknot 2 was well supported and similar in overall design to pk1. Helical sections 6b and 6c showed a potential to form a continuous helix in *Thermotoga maritima*. In beta-Proteobacteria, 6b was replaced by a short hairpin6d [1]. Helix 6d was observed also in three tmRNAs of the gamma-Proteobacteria *Acidithiobacillus ferroxidans *and *Francisella tularensis*.

### Pseudoknot 3 (helices 8 and 9)

Pseudoknot 3 was well supported but missing in Cyanobacteria and the organelles (Table 1). Helical sections 8a and 8b were likely to be continuously stacked because a single helix was present in some species such as *Aquifex aeolicus*. The unusual purine-rich internal loop between helical sections 8a and 8b was present in most gamma-Proteobacteria suggesting a special function.

### Pseudoknot 4 (helices 10 and 11)

This feature was well supported and was similar in design to the other tmRNA pseudoknots. Helical sections 10a and 10b had the potential to stack because a single helix was present in *Prevotella intermedia*. In some Cyanobacteria sequences, however, pk4 was replaced with two smaller tandem pseudoknots.

### Secondary structure prediction of the MLD

Because CSA was unable to determine secondary structure for a large portion of the MLD, energy calculations were carried out aimed to predict structure for the single-stranded portion of the open reading frame. The region corresponding to residues 79–107 of *E. coli *tmRNA (Figure 1) was extracted from the alignment. A representative alignment of 197 sequences was submitted to Mfold [29]. Each sequence had the potential to form at least one helix, designated "m" (see Figure 1, additional file 5: Banthracis2D.pdf, and additional file 6: Ccrescentus2D.pdf). Two or more adjacent helices were predicted for 17 sequences. The number of basepairs varied from two in *Chloroflexus aurantiacus *to ten in *Mycoplasma pulmonis*.

### Secondary structures of three representative tmRNA molecules

Secondary structures were determined for all sequences in the alignment but only three were extracted, diagrammed, and processed for 3-D modeling.

### Secondary structure of *E. coli *tmRNA

The 363-nucleotide tmRNA of the gamma-Proteobacterium *Escherichia coli *represented the typical tmRNA containing the TLD, the MLD, and four pseudoknots (pk1 to pk4) encompassing the pseudoknot domain (PKD). The 90-GCA-92 resume triplet coded for alanine. Two in-frame UAA stop codons (positions 120–125) were located within the terminal loop of helix 5 (Figure 1). Three basepaired regions (shown boxed) were only weakly supported by CSA. Helixm (residues 87–98) was predicted only by energy calculations. A slightly different helix involving residues 88–100 has been suggested by footprinting of *E. coli *tmRNA [30]. The evidence for the 112U-A133 basepair was weak, but was included due to the possibility of extending helix 5 (Materials and Methods). Helical section 5a (residues 108–113 and 134–137) was enlarged by the weakly supported 108G-C137, 110U-A135 and 111U-G134. The 109C-G136 pair was disproved. In helix 10ab, the basepair between 256G-C275 was only weakly supported. Helix 10ab (residues 248–256 and 274–283) could be extended by the boxed 257U-G274 pair.

### Secondary structure of *Bacillus anthracis *tmRNA

Overall, the secondary structure of *Bacillus anthracis *tmRNA (see additional file 5: Banthracis2D.pdf) was similar to that of *E. coli*. One notabledifference was a bulged uridine (U57) between helical sections 4a and 4b in pk1. A three-basepair helixm was predicted. The resume triplet (residues 89–91) coded for glycine, and the UAA stop codon was located at residues 119–121. Two weakly-supported pairs (108C-G132 and 109U-A131) extended helical section 5a.

### Secondary structure of *Caulobacter crescentus *tmRNA

*Caulobacter crescentus *tmRNA (additional file 6: Ccrescentus2D.pdf) consisted of two chains, A and B, of 213 and 83 residues, respectively. The resume codon (82-GCG-84 of chainA) coded for alanine and was followed by a UAA stop codon at residues 121–123. Helical sectionsm1 and m2 were predicted by energy calculations. There was weak support for 5a (G109-U135 and 111C-G133 in chainA), and the 106U-A138, 107U-A137, 108C-G136, and 110C-G134 in chainA were disproved. The pseudoknots were relatively small. Helix 11 corresponded to the absent pk4 (residues 1–18 in chain B).

### Tertiary structure modeling and visualization of tmRNA

ERNA-3D, a program developed to model RNA in three dimensions [11], was used on an SGI workstation as described in Materials and Methods. *E. coli *tmRNA was selected because this tmRNA is the subject of extensive research. *B. anthracis *tmRNA was chosen as an example of a tmRNA from a Gram-positive bacterium, and *C. crescentus *tmRNA was selected it represents a two-part tmRNA.

In order to create the initial models, the sequence and basepairing information were entered into an ERNA-3D input file to automatically generate A-form RNA for the helices sections and specify the single-stranded regions using ERNA-3D's algorithm [11]. Since ERNA-3D avoided an XYZ coordinate system as reference for the user, the manipulation of the model from the viewer's perspective was simple and intuitive. The coordinates of each model were saved in PDB format [31] for compatibility with other molecular modeling programs. Motifs (listed in Tables 2 to 4) were selected to model the loops and turns of a particular tmRNA. ERNA-3D selection files were generated to define clusters and place the motif in 3-D without disturbance to the rest of the model. The 3-D cursor box was used to manipulate a cluster in three-dimensional space, similar to the manipulation of a section of a physical model.

Numerous high-resolution structures determined by NMR or X-ray crystallography represented a rich source of detailed information for defining biologically meaningful motifs. The SCOR database [12] provided a way to find suitabletemplates. In rare cases when a SCOR search for a motif did not result in an acceptable match (e.g. motif 9, Table 2), the nucleotides were positioned manually in ERNA-3D. Otherwise, the coordinates were obtained from the Protein Data Bank PDB [32], extracted using the program Swiss-PDBViewer [33], and imported into ERNA-3D. The source motif and the region to be modeled were selected as separate clusters and aligned in three dimensions using common features (usually a shared basepair). Once superimposed, the coordinates of the residues in the source motif were copied onto the corresponding residues in the model. The template was then deleted, leaving a biologically meaningful structure. The backbone connections between the motif and the rest of the model were inspected visually and, if needed, manual adjustments were made to correct bond lengths and tetrahedral angles involving the phosphorous atom at the joint between the extracted motif and the helical structures generated by ERNA-3D.

As an example of the motif modeling process, the purine-rich loop in *E. coli *pk3 (positions 204–206 and 223–225) was constructed using a similar loop in the 30S ribosomal subunit. First, the purine-rich loop was defined as motif 11a (Table 2), and used to search the SCOR database. Positions 780–782 and 800–802 in the structure of the *Thermus thermophilus *30S ribosomal subunit [34] were found to conform to the motif. The 30S ribosomal subunit coordinates (1J5E.pdb in this case) were downloaded from the PDB and displayed using Swiss-PDBViewer. The coordinates of the loop and the closing basepairs were extracted and inspected to confirm that the structure was compatible. The clustered regions were aligned with the ends of helical sections 8a and 8b at the basepairs 203U-G226 and 207A-U222 of the *E. coli *model and 779C-G803 and 783C-G799 of the template. Template positions 780-AAA-782 and 800-GUA-802 were then copied onto 204-GGA-206 and 223-GAA-225 of the model. The template was deleted and the bond lengths and angles involving the atoms of the phosphates of residuesU203, G222, A206, and U222 were adjusted.

In some instances, the tmRNA sequence alignment was reinvestigated using ideas derived from the 3-D model. For example, the alignments of pk1 in *Bacillus anthracis *tmRNA and relatives was changed from a two nucleotide bulge (56-AU-57) between helical sections 4a and 4b to a more feasible and equally well supported one-nucleotide bulge (U57, see additional file 5: Banthracis2D.pdf). The alignment of helix 10 in pk3 in *B. anthracis *tmRNA and relatives was altered from a 237C-A269 mismatch and an asymmetrical loop (C239 and 266-GU-267) to a single looped-out C269. The alignment of pk3 of *Caulobacter crescentus *and relatives was changed from four basepairs and a weakly supported fifth pair in helix 8 (between 174G-C196 of chainA) to the four basepair structure seen in additional file 6: Ccrescentus2D.pdf. Information about spatial neighborhoods as obtained from cross-linking, site-directed mutagenesis, and functional testing of *E. coli *tmRNA was introduced and is described in detail below. All models were inspected visually for correct bond angles and distances around the phosphorous atoms at the joints between the extracted motifs and the helical regions generated by ERNA-3D. The coordinates are provided as additional file 7: Ecoli-closed.pdb, 8: Ecoli-open.pdb, 9: Banthracis-closed.pdb, and 10: Ccrescentus-closed.pdb.

### 3-D model of *E. coli *tmRNA

**Figure 3:**
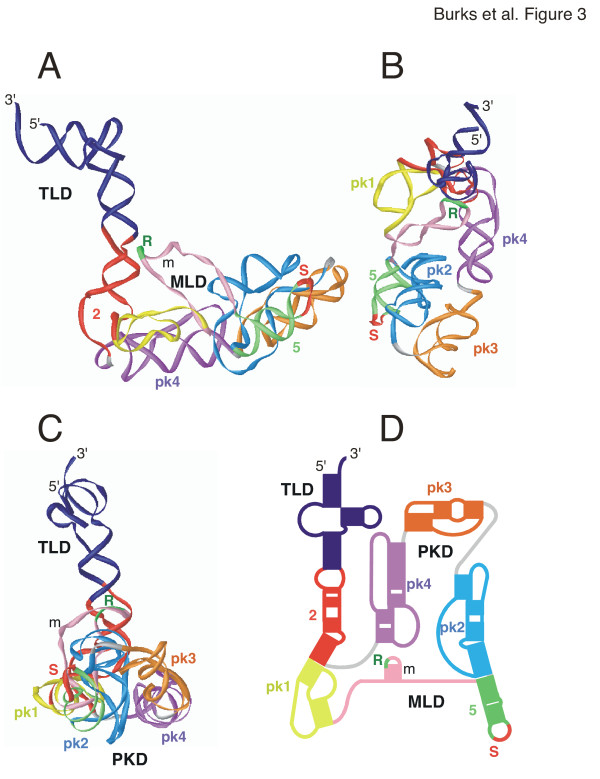
**3-D model of *Escherichia coli *tmRNA**. The 3-D model of *Escherichia coli *tmRNA is viewed as a ribbon diagram from the side in panel A, the top in panel B, and in panel C turned by approximately 90° around the y-axis in relation to A. Panel D shows a representation of the corresponding 2-D structure using the identical coloring scheme. Labeled are the 5' and 3' ends, the resume (R) and stop codons (S), and the three domains (TLD, MLD, PKD). The figure was produced with iMol [53] and the PDB coordinates of additional file 7: Ecoli-closed.pdb.

The model shown as a ribbon diagram in Figure 3 consists of a compacted MLD and PKD with the TLD extending from the body of the molecule due to the near-coaxial stacking of the helix 2 sections. The coordinates for the TLD were taken from a previous model [35] which is based on two cross-linked sites, one formed between nucleotides U9/U10 near the 5' end and nucleotides C346/U347 in the T loop, the other involving residues at positions 25–28 and 326–329 within helix 2a (motif 2 in Table 2). Important features of the TLD include the non-Watson-Crick base pairs formed by 19-GA-20 and 333-GA-334 which have been confirmed by site-directed mutagenesis [36].

A very efficient UV-induced cross-link observed between the stop codon loop of helix 5 and pk2 of *E. coli *tmRNA (Wower et al., unpublished) introduced a considerable constraint of helices 5, 6, and 7, and, as has been shown recently, is consistent with the cryo-EM structure of ribosome-bound tmRNA of the initial stage of *trans*-translation [10]. Also considered was the previously-discovered covariation [37] between C44 and C66 (*E. coli *numbering, Figure 1) which determines the orientation of helix 2 in relation to helix 3 and thus the approximate angle by which the TLD protrudes. The 44/66 covariation is strongly supported (26 covariations *versus *four mismatches) in an alignment of 143 representative sequences (not shown). Since this is a non-Watson-Crick covariation, it is difficult to propose a precise structure in this region. More extensive studies will be required to better understand the nature of the 44/66 covariation.

Overall, the distance between the 3'end and A231 in pk3 was 180 Å, and 70 Å between the outside edges of pk1 and pk4. Helix 5 and pk2 were positioned in a parallel fashion. The nucleotides in the bulge between helical sections 6a and 6b (motif 9, Table 2) were adjusted manually to allow for a close fit of helix 5 and pk2. This model is supported by the finding that mutations that destroy base pairing in helix 5 substantially decrease tmRNA tagging activity (Wower et al, in press) and abolish the long-distance interaction between helix 5 and pk2 as judged by the absence of a cross-link between the stop codon loop of helix 5 and pk2 (Wower, unpublished data). Evidence for the existence of helix 5 has been provided by the analysis of compensatory mutations which completely restore tagging [28]. Each of the four pseudoknots displayed the previously determined structural properties characterized by extensive helical stacking [27]. The MLD and pseudoknots were arranged in a central loop with the resume codon positioned near the junction between helices 2a and 2b (motif 3a, Table 2). Our model reflects the finding that the pseudoknots are functionally interchangeable [38] and thus are likely to retain a considerable level of structural independence. Furthermore, data derived from cross-linking experiments showing that pk2 and pk3 are in close proximity whereas helix 5 and pk4 are further apart (Wower, unpublished data) agree with the presented model.

### 3-D model of *B. anthracis *tmRNA

**Figure 4:**
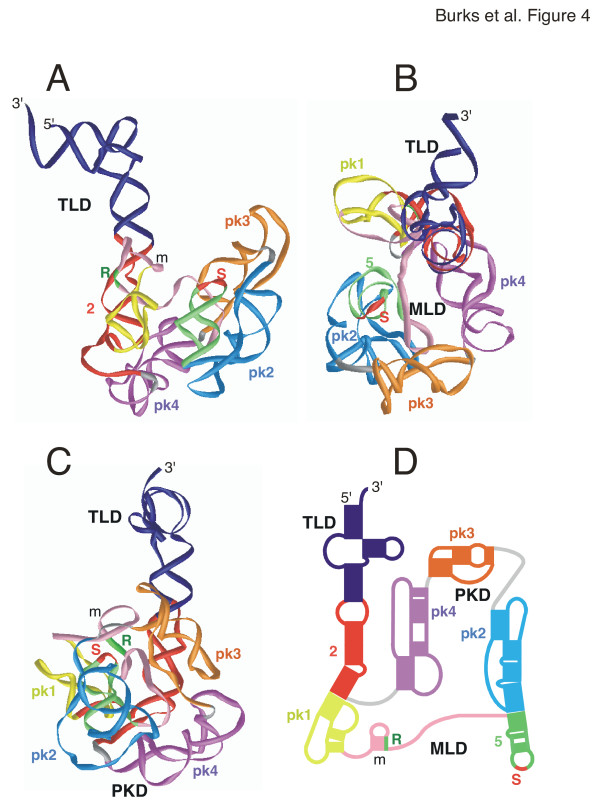
**3-D model of *Bacillus anthracis *tmRNA**. The 3-D model of *Bacillus anthracis *tmRNA is viewed as a ribbon diagram from the side in panel A, the top in panel B, and in panel C turned by approximately 90° around the y-axis in relation to A. Panel D shows a representation of the corresponding 2-D structure using the identical coloring scheme. Labeled are the 5' and 3' ends, the resume (R) and stop codons (S), and the three domains (TLD, MLD, PKD). The figure was produced with iMol [53] and the PDB coordinates of additional file 9: Banthracis-closed.pdb.

The 3-D model of the *B. anthracis *tmRNA (Figure 4) was similar to the *E. coli *model. A sharper angle was modeled between helix 2 axis and the PKD. The dimensions were 150 Å from the 3'end to the distant edge of pk2, and approximately 80 Å between the outer edges of pk2 and pk4, respectively.

### 3-D model of *C. crescentus *two-part tmRNA

**Figure 5:**
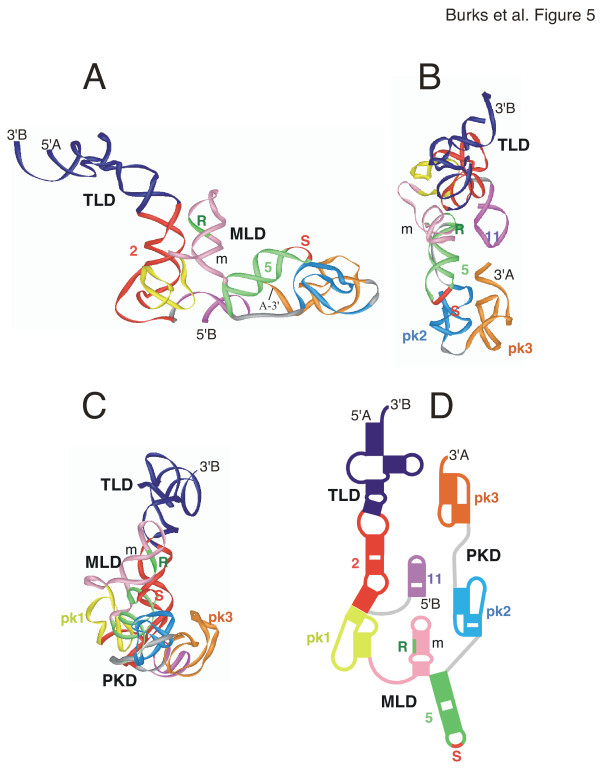
**3-D model of *Caulobacter crescentus *tmRNA**. The 3-D model of *Caulobacter crescentus s *tmRNA is viewed as a ribbon diagram from the side in panel A, the top in panel B, and in panel C turned by approximately 90° around the y-axis in relation to A. Panel D shows a representation of the corresponding 2-D structure using the identical coloring scheme. Labeled are the 5' and 3' ends, the resume (R) and stop codons (S), and the three domains (TLD, MLD, PKD). The figure was produced with iMol [53] and the PDB coordinates of additional file 10: Ccrescentus-closed.pdb.

Compared to the other two tmRNA models, the two-part tmRNA *Caulobacter crescentus *model (Figure 5) was slightly more elongated. It measured 195 Å from the 3'-end of chainB to the single-stranded region between pk2 and pk3. The distance between helix 5 and the 3'end of chainA was 55 Å.

## Discussion

We have compared a growing number of tmRNA sequences from all groups of bacteria to produce an alignment from which the secondary structure of any tmRNA could be easily extracted. Most basepairs were supported by phylogenetic evidence, whereas only a few helical sections required energy calculations. Uncertainties in assignment of basepairs, such as the pseudoknot region of chloroplasts and one-piece cyanobacterial tmRNAs, may be eliminated in the future when more sequences will become available.

The common layout of the secondary structures indicated a similar function in all bacteria. The number and size of the pseudoknots varied, supporting the idea that the pseudoknots may only enhance the essential functions carried by the TLD and the MLD [28]. Differences in the secondary structure features were usually not random but occurred between groups of related organisms. For example, helix 6d was present only in the beta- and three close relatives of the gamma-Proteobacteria. Whether these group-specific features are responsible for differences in the *trans*-translation mechanism remains to be determined. However, strategies that exploit these differences, for example for developing new antibiotics targeted at a specific group of bacteria, can now be envisioned.

In principle, tertiary structure models of any tmRNA in the alignment could be built using the described procedures. Here, we have shown how to generate a biologically more meaningful model of *E. coli *tmRNA which represents a significant progress from a previous model [27]. We also constructed 3-D model of the tmRNAs of *B. anthracis *and *C. crescentus*. Overall, the three models were similar in shape and size confirming that all tmRNAs have the potential to function similarly in *trans*-translation. The TLD mimicked the L-shape of canonical tRNA [39] and may be necessary for proper association tmRNA with the EF-Tu, SmpB, and subsequent binding to the ribosomal A-site. The lack of a D-stem has been suggested to confer flexibility [40], but SmpB may be responsible for stabilizing this region [41,42].

Differences in the shapes of the three tmRNA models (e.g. the angle between helix 2 and the main body of the molecule) may be due to the difficulty in determining the precise arrangement of the pseudoknots. Considering that the pseudoknots are likely to constitute relatively independent structural units, conformational changes might occur around the connecting single strands, as well as in the MLD and the weakly-supported helix m. TmRNA may become less flexible when bound to proteins such as SmpB and ribosomal protein S1. EF-Tu, however, likely binds to the coaxially-stacked helices 1 and 12 [43], and therefore appears to have little effect on the conformation of the TLD. Protein SmpB was found to bind near helix 2a [41,44], has two RNA binding sites [45], and thus could make additional contacts with other regions. Protein S1 is the largest ribosomal protein, has been shown to be close to numerous sites, and to be required for the binding of tmRNA to the ribosome [7]. Since S1 is a flexible, beadlike protein [46] it may not restrict the conformational potential of the tmRNA molecule. Instead, the protein may instill some constraint to the large central loop formed by the PKD and the MLD. Because S1 is known to melt helices in mRNAs [47], it is also possible that it unwinds helix m and exposes the resume codon and the preceding nucleotides U85 and A86 for efficient *trans*-translation [48,49].

**Figure 6:**
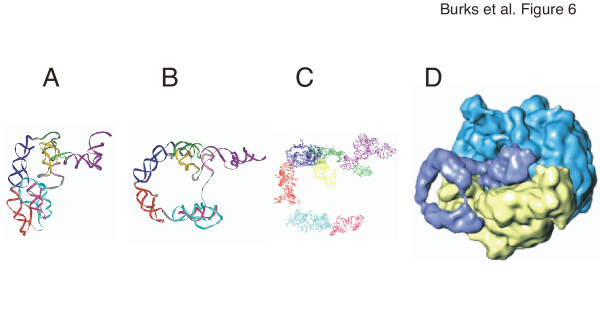
**Conformational changes in *Escherichia coli *tmRNA**. Panel A: closed form of the *E. coli *model as shown in Figure 3. Panel B: open conformation adjusted to more closely resemble the ribosome-bound form as determined by cryo-EM [10] using additional file 8: Ecoli-open.pdb. Panel C: coordinates extracted from the cryo-EM model [10]. The TLD is shown in dark purple, helix 2 in green, pk1 in yellow, helix 5 in pink, pk2 in turquoise, pk3 in red, and pk4 in dark blue. Panel D: Electron density map of the 50S subunit in light blue, the 30S subunit in yellow, and the bound tmRNA (in the absence of ribosomal protein S1) in dark blue (from [10]).

The tmRNA models show the resume codon in close proximity to the internal loop formed between helical sections 2a and 2b. This arrangement would allow the ribosome to "jump" a relatively short distance from the end of the broken mRNA onto the ORF of tmRNA. In a recent cryo-EM study of the initial stage of *trans*-translation [10], the tRNA-like region, SmpB, EF-Tu, and part of pk4 were located in the A-site of the ribosome. We suggest that this more open arrangement is made possible due to the flexibility of tmRNA, the melting of helix m, and/or a change in conformation induced by the binding of tmRNA to the ribosome (Figure 6). The opening of the central loop seems to be accompanied by a rotation of the TLD around the helix 2 axis (compare Figure 6A and 6B) and thus might properly align the resume codon with the 3'-end of broken mRNA in the ribosomal decoding centre. At the later stages of the transit of tmRNA across the ribosome even more dramatic conformational changes were shown to disrupt helix 5 and the pseudoknotted regions (Wower et al, in press). These downstream alterations are likely mediated not by protein S1 but by the intrinsic helicase activity of the ribosome [50] and are required to maintain the ribosomal subunits in close proximity to the unfolded tmRNA in order to monitor *trans*-translation.

## Conclusion

This study significantly advances our understanding of *trans*-translation by providing biologically feasible 3-D models for the entire tmRNA molecule. Although the modeling of only three tmRNAs has been described here, 3-D models of every tmRNA can be extracted from the alignment. The models are characterized by a functionally significant features, including biologically relevant structures for the single-stranded regions and the close proximity between the TLD and the resume codon. Conformational changes induced by binding of tmRNA to SmpB, ribosomal protein S1, and the ribosome suggest a transformation of a free compact tmRNA to a more open ribosome-bound structure. The comparative modeling approach described here for tmRNA is easily adapted for other RNA classes.

## Methods

### Comparative sequence analysis

The tmRNA sequences were arranged in phylogenetic order using information available in the RDP [19]. When the phylogenetic order could not be determined, the sequence was placed next to the closest relative as determined by the ClustalW plug-in of BioEdit [18,51]. The sequences were made available at the tmRDB [52].

Aligning was done manually using BioEdit [18] with details described previously [13]. Briefly, closely related sequences were aligned first. Then, invariant positions were used as guides to align the dissimilar regions. Next, common secondary structure elements were identified by observing covariations and find support for basepairs, tertiary interactions, or other structural features. Compensatory base changes (CBCs) were observed if a change in one residue of a Watson-Crick or G-U pair was compensated by a second change to maintain basepairing. Two residues were mismatched if they did not form a Watson-Crick or G-U pair. CBCs and mismatches were counted to determine positive and negative evidence in order to prove or disprove the existence of a particular pair. A basepair was considered proven if there was at least twice as much positive than negative evidence. Invariant pairs provided neither positive nor negative evidence. If a basepair was proven in one phylogenetic group and disproved in another group, the basepair was considered to be specific to that group.

The alignment and suggested CBCs were checked using RNAdbTools [22] to eliminate incorrectly-paired nucleotides, suggest extensions of helices, and determine the phylogenetic support for each basepair. Weakly supported basepairs adjacent to supported basepairs were considered an extension of the helix and usually included in the secondary structures (Figure 1, additional file 5: Banthracis2D.pdf and 6: Ccrescentus2D.pdf).

### 3-D model building

The secondary structure information was used as input for ERNA-3D [11] installed on an SGI workstation running IRIX 6.5. ERNA-3D generated A-form RNA for each helix and calculated the conformations of single-stranded regions. The models were examined using CrystalEyes stereovision goggles and an StereoGraphics infrared emitter. Structural motifs were identified using SCOR [12], the coordinates were obtained from the Protein Data Bank (PDB) [32], extracted using Swiss-PDBViewer [33], and superimposed onto the model. Data obtained from site-directed mutagenesis, cross-linking experiments, or the literature were incorporated, and bond lengths and angles were adjusted manually to produce biologically feasible models. The final models were saved in PDB format (additional files 7: Ecoli-closed.pdb, 8: Ecoli-open.pdb, 9: Banthracis-closed.pdb, and 10: Ccrescentus-closed.pdb and viewed in iMol [53] to create the ribbon diagrams shown in Figures 3 to 5.

## Authors' contributions

JB identified and aligned a large number of the tmRNA sequences, carried out the 3-D modeling experiments, and drafted portions of the text. CZ conceived the study, participated in all of its aspects, and wrote the final manuscript. FM developed the ERNA-3D program and incorporated new functions to allow comparative modeling of RNA. IW developed assays which allowed to test the biological significance of the *Escherichia coli *tmRNA model. JW participated in the coordination of the study and provided RNA-RNA cross-link data which were crucial for constraining the models in 3-D. All authors read and approved the final manuscript.

## Supplementary Material

Additional File 1 tmRNA alignment. Species names are shown on the left with their tmRDB ID (see ). Supported base pairing are shown with upper case letters and are indicated on the bottom. Secondary structure features are indicated on the top. Click here for file

Additional File 2 tmRNA alignment. Species names are shown on the left with their tmRDB ID (see ). Supported base pairing are shown with upper case letters and are indicated on the bottom. Secondary structure features are indicated on the top. Each sequence is shown on a single long line suitable for import into other programs. Click here for file

Additional File 3 tmRNA alignment. Species names are shown on the left with their tmRDB ID (see ). Supported base pairing are shown with upper case letters and are indicated on the bottom. Secondary structure features are indicated on the top. The alignment is shown in sections for easier viewing and printing. This arrangement is not suited for import into other programs. Click here for file

Additional File 4 tmRNA alignment. tmRNA alignment in msf format suitable for import into editors which support the msf format. Can be used to convert into other sequence formats using, for example the Readseq server at Click here for file

Additional File 5 Secondary structure of *Bacillus anthracis *tmRNA. Phylogenetically-supported helices are highlighted in gray and numbered from 1 to 12. The 5' and 3' ends are indicated. Arrows represent connections from 5' to 3'. Residues are numbered in increments of ten. Weakly supported regions and basepairs are show in boxes. The star labels the first nucleotide of the resume codon. The tag peptide sequence is shown below the mRNA-like region. The stop codon is indicated with a solid arrowheads. Three domains are distinguished: The tRNA-like domain (TLD), the mRNA-like domain (MLD), and the pseudoknot domain (PKD). Click here for file

Additional File 6 Secondary structure of *Caulobacter crescentus *tmRNA. Phylogenetically-supported helices are highlighted in gray and numbered from 1 to 12. The 5' and 3' ends of both chains are indicated. Arrows represent connections from 5' to 3'. Residues are numbered in increments of ten. Weakly supported regions and basepairs are show in boxes. The star labels the first nucleotide of the resume codon. The tag peptide sequence is shown below the mRNA-like region. The stop codons are indicated with solid arrowheads. Three domains are distinguished: The tRNA-like domain (TLD), the mRNA-like domain (MLD), and the pseudoknot domain (PKD). Click here for file

Additional File 7 PDB coordinates of the closed form of *Escherichia coli *tmRNA. 3-D model of the closed form of *Escherichia coli *tmRNA. Click here for file

Additional File 8 PDB coordinates of the open form of *Escherichia coli *tmRNA. 3-D model of the open form of *Escherichia coli *tmRNA. Click here for file

Additional File 9 PDB coordinates of the closed form of *Bacillus anthracis *tmRNA. 3-D model of the open form of *Bacillus anthracis *tmRNA. Click here for file

Additional File 10 PDB coordinates of the closed form of *Caulobacter crescentus *tmRNA. 3-D model of the closed form of *Caulobacter crescentus *tmRNA. Click here for file

## Figures and Tables

**Figure 2:**
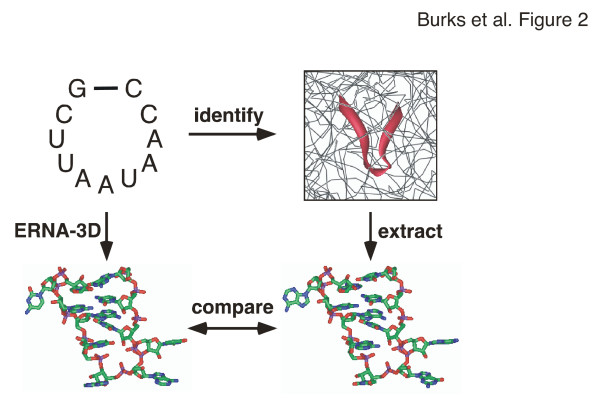
**Motif modeling procedure**. Motifs, for example the nonamer-loop shown in the top-left panel, were identified in the known high-resolution structures (top-right) with the help of SCOR [12]. The PDB coordinates were extracted (bottom-right) and compared with the 3-D model generated by ERNA-3D (bottom-left) to deduce relevant models.

**Table 1:** Phylogenetic distribution of tmRNA features

**Phylogenetic Group**	**TLD**	MLD	**pk1**	**pk2**	**pk3**	**pk4**	**Other**
Thermophilic Oxygen Reducers							
Thermatogales							
Green Non-sulfur & Bacteria elatives							
Flexibacter Cytophaga Bacteroides							
Green Sulfur Bacteria							
Planctomyces & elatives							
Cyanobacteria					**1**	**1,2**	**3**
Plastids					**-**	**-**	
Mitochondria		**-**	**-**	**-**	**-**	**-**	
Fibrobacter/Acidobacter & elatives							
Spirochetes & elatives							
Proteobacteria, alpha				**4**		**5**	**3**
Proteobacteria, beta				**6**			**3**
Proteobacteria, gamma				**6**			
Proteobacteria, delta							
Proteobacteria, epsilon							
Fusobacteria							
Gram Positive Bacteria							

The tRNA-like domain (TLD), mRNA-like domain (MLD), and the four pseudoknots pk1 to pk4 are shown on the top. Other features peculiar to a phylogenetic group are in the right column. White fields indicate the presence, dashes the absence of a feature. Numbers suggest the following structural features: (1) certain Cyanobacteria lack these pseudoknots. (2) One-chain cyanobacterial tmRNAs contain two smaller tandem pseudoknots named pk4a and 4b. (3) The tmRNAs of some species in this group consist of two basepaired molecules [14, 21]. (4) The genus *Ricketsia *and its relatives lack pk2. (5) pk4 of the alpha-Proteobacteria has been reduced to a single helix (named helix 11). (6) Some species in this group contain an additional helix (helix 6d).

**Table 2:** Structural motifs used for the *Escherichia coli *tmRNA model

**Motif**	**SCOR class**	**tmRNA Res.**	**Source Res.**	**Coordinates**	**Comments**
**1**		1–7, 353–363	1–7, 12–22	1IKD.pdb (chain W)	ACCA end and G3-U357 pair
**2**		8–28, 325–352	8–28, 325–352	tmx-34.pdb from tmRDB	
**3a**	*internal loop with unpaired stacked bases*	29–33, 321–324	1775–1779, 1765–1768	1JJ2.pdb	
**3b**	*stacked duplex with one non-WC pair*	C35, A319		ERNA-3D	
**4**	*stacked duplex with two non-WC pairs*	38–39, 315–316	2874–2875, 2882–2883	1JJ2.pdb	
**5**		309–311		ERNA-3D	
**6a**	*pseudoknot*	49–78	1–33	1RNK.pdb	pk1
**6b**	*tetraloop*	87–98	5–8	1AFX.pdb	the only YRRR tetraloop in SCOR
**7**	*nonaloop*	118–126	1834–1842	1JJ2.pdb	
**8**	*one unpaired and stacked U*	U131	U30	1B36.pdb	
**9**		171–174		ERNA-3D	
**10a**	*stacked duplex with two non-WC pairs*	149–150, 165–166	288–289, 363–364	1JJ2.pdb	
**10b**	*pseudoknot*	138–196		**6a**	pk2
**11a**	*internal loop*	204–206, 223–225	780–782, 800–802	1J5E.pdb	
**11b**	*pseudoknot*	197–247		**6a**	pk3
**12**	*stacked duplex with one non-WC pair*	G258, A273	A-G6, B-A27	420D.pdb	
**13a**	*stacked duplex with one non-WC pair*	C266, U296		ERNA-3D	
**13b**	*pseudoknot*	248–299		**6a**	pk4

Shown in columns one to four are the motif numbers in bold, their SCOR classification [12], the residue positions in the tmRNA model and the source structure. column five lists the filenames containing the atomic coordinates that were derived from the PDB [31], the tmRDB [52], or were generated by ERNA-3D [11].

**Table 3:** Structural motifs used for the Bacillus anthracis tmRNA mode

**Motif**	**SCOR class**	**tmRNA Res.**	**Source Res.**	**Coordinates**	**Comments**
**1**		1–11, 12–27, 316–355	1–11, 13–28, 324–363	*E. coli ***1**	ACCA end and G3-U349 pair
**2**	*loop with stacked interdigitated bases*	28–33, 312–316	477–482, 451–455	1J5E.pdb	
**3**		300–301		ERNA-3D	
**4a**		43–53, 61–65, 294–299	43–53, 63–67, 302–308	*E. coli *model	helix 2d, helix 3
**4b**	*pseudoknot*	58–60, 66, 67–73	12–14, 20, 23–29,	1RNK.pdb	pk1, helix 4b
**4c**		54–56, 74–75	54–56, 77–78	*E. coli ***6a**	pk1, helix 4a
**5**	*heptaloop*	82–88	335–341	1JJ2.pdb	
**6a**	*one looped out A*	A112	210	1GID.pdb	
**6b**		C114, C127		ERNA-3D	
**6c**	*octaloop*	117–124	1499–1506	1JJ2.pdb	
**7a**		134–138, U163, A164, 165–169,	140–144, A171, A174, 175–179	*E. coli *10b	helix 6a, looped out U163 and A164.
**7b**	*one looped out U*	U158	U87	1JJ2.pdb	
**7c**		144–147, 148–156, 180–185	259–262, 264–272, 293–298	*E. coli ***13b**	helix 6c, helix 7, pk2
**8**		G133, C170, 171–177, 178–179	C138, G181, 182–188, 190–191	*E. coli ***10b**	
**9a**		C190, 191–198, 208–215, 216	G200, 203–210, 219–226, U229	*E. coli ***11a**, **11b**	purine-rich loop, pk3
**9b**	*pseudoknot*	202–206, 228–232	9–13, 28–32	1RNK.pdb	pk3, helix 9
**10a**		199–201	154–156	*E. coli ***6a**	pk3
**10b**		217–227	229–239	*E. coli ***6a**	pk3
**11a**	*one looped out C*	C269	C6	1BVJ.pdb	
**11b**	*stacked duplexes with four non-WC pairs*	241–245, 260–263	795–798, 815–818	1JJ2.pdb	
**12**		246–259, 275–290	259–277, 284–299	*E. coli ***6a**	helix 10c, helix 11ab, pk4

Motif numbers in bold, their SCOR classification [12], residue positions in the tmRNA model and the source structure are shown in columns one to four. Column five lists the filenames containing the atomic coordinates that were derived from the PDB [31], the *E. coli *model (see Table 2), or were generated by ERNA-3D [11].

**Table 4:** Structural motifs used for the *Caulobacter crescentus *tmRNA model

**Motif**	**SCOR class**	**tmRNA Res.**	**Source Res.**	**Coordinates**	**Comments**
**1**		A-1-13, A-14-21, B-50-83	1–13, 15–22, 322–355.	*B. anthracis ***1**	-
**2a**	*one non-Watson Crick pair and one looped-out A*	A-22, B-48-49	14, 4–5	5MSF.pdb	disruption in anticodon stem
**2b**	*loop with base triple*	A-26-31; B-40-44	8–13, 24–28	1FMN.pdb	
**2c**		A-C36, B-C35		ERNA-3D	
**3**		A-40-41, B-27-31		ERNA-3D	
**4**		A-47-61, A-62-70	196–210, 224–232	*B. anthracis ***8a, 8b, 9a, 9c**	pk1
**5**		A-87-93	82–88	*B. anthracis ***5**	terminal heptaloop of helix m2
**6**	*one looped out A and one or more non-WC pairs*	A-78-81, A-99-101	113–116, 205–207	1GID.pdb	internal loop between helices m1 and m2
**7**		B-8-11	284–285, 291–292	1D6K.pdb	
**8a**		A-112-114, A-131-132		ERNA-3D	
**8b**		A-119-125	82–88	*B. anthracis ***5**	
**9a**		U148, C163		ERNA-3D	
**9b**		A-156-165, A-146-153, A-167-171	157–166, 149–156, 192–196	*E. coli ***10b**	pk2. Single-stranded regions were adjusted to connect helices
**10**		A-154-156, A-170-171	54–55, 76–77	*E. coli ***6a**	Correction of two pairs in pk2 based on *E. coli *pk1
**11**		A-211-214, A-177-182, A-184-193, A-177-178, A-192-198, A-194-209	360–363, 207–212, 213–222, 200–201, 228–234, 230–245	*E. coli ***1 **(ACCU tail) and 11b	pk3 and ACCU tail of pk3

Motif numbers in bold, their SCOR classification [12], residue positions in the tmRNA model and the source structure are shown in columns one to four. Column five lists the filenames containing the atomic coordinates that were derived from the PDB [31], the *E. coli *or *B. anthracis *models (see Tables 2 and 3), or were generated by ERNA-3D [11].
