# Supplementary material for: Comparative 3-D Modeling of tmRNA
Source: BMC Mol Biol. 2005 Jun 15;6:14. doi: 10.1186/1471-2199-6-14 (PMC1168896; doi:10.1186/1471-2199-6-14)
Supplement: Additional File 1 — tmRNA alignment. Species names are shown on the left with their tmRDB ID (see ). Supported base pairing are shown with upper case letters and are indicated on the bottom. Secondary structure features are indicated on the top. [file 1471-2199-6-14-S1.html]

 tmRNA alignment


```
p-pre              aaaaaaa--------------bbbbb-bbb-------ddddd---eeeee---ffffffggggg-----hhhhhhh-------ggggg-----------hhhhhhh--------------------------------------------------------------------------------------------------iiiiiiiiijjjj-------------------------------------------jjjj--iiiiiiiii-------------------------------------kkkkkkk--------lllllmmmmmm-----------nnnnnn-mmmmmm---------------oooo------------oooo-lllll----------------kkkkkkk-------------nnnnnn---------ppppppp-----ppp--pppp----------qqqqqqqqqrrrrr----ssssssssrrrrr-qqqqqqqqq----------ssssssss----tttttuuuuu----vvv----wwww-xxxvvv---uuuuu-ttttt---zzz-zz--AAAAAzzzzz---AAAAABBBB--CCCCC--BBBB-----CCCCC--xxx--wwww----fff-fff------eeeee---ddddd---------bbbbbbbb----DDDDD-------DDDDD-aaaaaaa----
psknts             -----------------------------------------------------------((((((((((((((((((((pseudoknot-I)))))))))))))))-----------------------------rrr------------------------------------------------------------------------------------sss---------------------------------------------------------------------------------------(((((((((((((((((((((((((((((((((((((((((((((((((((((((((((((((((pseudoknot-II-))))))))))))))))))))))))))))))))))))))))))))))))))))))----------------------------------------(((((((((((((((((((((-pseudoknot-III-))))))))))))))))))))))----(((((((((((((((((((((((((((((((((((((((((((((((((((--pseudoknot-IV-)))))))))))))))))))))))))-))))))))))))))))))))--------------------------------------------------------------------------------
hel-10             ----------------------------------------------------------------------------------------------------------------------------------------------------------------------------------------------------------------------------------------------------------------------------------------------------------------------------------------------------------------------------------------------------------------------------------------------------------------------1111111-----111--1111-------------------------------------------------------------------------1111111111----111----1111-111111---11111111111---111-11--1111111111---111111111--11111--1111-----11111--111--1111---------------------------------------------------11111-------11111------------
hel-1              1111111--------------222222222-------2222222222222---22222233333-----4444444-------33333-----------4444444--------------------------------------------------------------------------------------------------5555555555555-------------------------------------------5555--555555555-------------------------------------6666666--------66666666666-----------777777-666666---------------6666------------6666-66666----------------6666666-------------777777---------7777777-----777--7777----------88888888888888----9999999988888-888888888----------99999999----0000000000----000----1111-111000---00000000000---333-33--4444433333---444445555--66666--5555-----66666--111--1111----2222222------2222222222222---------22222222----22222-------22222-1111111----
hel-x              ---------------------aaaaa-aaa-------bbbbb---ccccc---dddddd-------------------------------------------------------------------------------------------------------------------------------------------------aaaaaaaaabbbb-------------------------------------------bbbb--aaaaaaaaa-------------------------------------aaaaaaa--------bbbbbcccccc------------------cccccc---------------dddd------------dddd-bbbbb----------------aaaaaaa-----------------------------------------------------------aaaaaaaaabbbbb------------bbbbb-aaaaaaaaa----------------------aaaaabbbbb----ccc----aaaa-bbbccc---bbbbb-aaaaa----------------------------------------------------------bbb--aaaa----ddd-ddd------ccccc---bbbbb---------aaaaaaaa---------------------------------
Aqui.aeol.         GGGGGCGga-aaggauu-cgaCGGGG-ACaggcg---GUCCc---cGAGGa--GCAGGCCGGG-------UGGCU---------CCCGuaac--------AGCCG----------------------cuaaaacagcucccgaagcugaacuc------------------------------------------------------gcucuCGCUG----ccuaauuaaa--------------------------cggCAGC--G---------------------------------------------CGUCC-C--------CGGUA--GGUU----------uGCGGGU-GGCC--------------------------------------UACCG----------------GaGGGCGucag-----agacACCCGCuc-------------------------------------gGGCUACU--CGGUc----GCACGGG--GCUG---AGUAGCUgacacc--uaaCCCGUGC----UaCCCUCGGG--gaGCUu---GCCC-GUGGGCga-CCCGA-GGGgAaau-------------------------------------------------ccugaaCAC--GGGC-uaaGCC-UGUagag--CCUC-----GGAUguggcc-gccGUCCUCGgac-GCGGGuucgauuCCCGC-CGCCUCCacca
Ther.mari.         GGGGGCGaa--cggguu-cgaCGGGG-AUGga-----GUCCc---CUGGGaa-gCGAGCCGAGGu---cCCCACCU-------CCUCGuaaaaa-----AGGUGGGacaa----------------agaauaagugccaacgaaccuguu---------------------------------------------------------gcuguuGCCG----cuuaauagauaa--------------------------gCGGC------------------------------------------------CGUCCUC--------UCCGA--AGUU-----------GGCUG--GGCU--------------------------------------UCGGA-------a--------GAGGGCGugag-----agaucCAGCCua--------------------------------------CCGAUUCA-GcUUc----GCCUUCC--GGcC--UGAAUCGGgaaaac--ucaGGAAGGCug---UGGGAGaGGa-cacCCu---GCCC-GUGGGagg-UCcCU-CCCGagagcg-------------------------------------------------aaaaCAC--GGGC-ugcGCU-CGgagaag-CCCAG----GGGCcu------cCAUCUUCGgac-GGGGGuucgaauCCCCC-CGCCUCCacca
Chlo.aura.-1.      nnnnnnnnnnnnnnnnnnnnnnAGGG-AGUgaac---GCCG----UAGACu--GCAGGUCGUGc----cGCCCagU--------CACGuaaaa------G--GGGCaa-----------------ggcaacaacugccaacaccaacacucgggcccaggcucgccug---------------------------------------gcccuCGCCG----cuuaauaauag----------------------------CGGC--G--------------------------------------------cGCUCGcCguagcucccCcGA--UGGU-----------UAC-GG-GUCGagcg----------------------------------UCaGca---------------GuCGGGUgcu----------CCgGUA--------------------------------------acgccugcCU-gAGC-----GUU--AUCgGCU--aAGa---------------GAUcaGGC-----UgGCccaacg-guCGCuuu-GCCa-CUCGUG---cgacu-GCgGgugaga-----------------------------------------------ugaaacGAG--gGGC-uaaACC-UGUagau--GUCUG----CGGUcua------GCUUUCUggac-ggggnnnnnnnnnnnnn-nnnnnnnnnnn
Chlo.aura.-2.pa.   ----------------------------------------------------------------------------------------------------------------------------------------------------------cccaugaucgccug---------------------------------------gcccuCGCCGcuu-cauaauag-------------------------------UGGC--G--------------------------------------------cGCUCGcCguagcucccCcGA--UGGU-----------UAC-GG-GUCGagcg----------------------------------UCaGca---------------GuCGGGUgcu----------CCgGUA--------------------------------------acgccugcCU-gAGC-----GUU--AUCgGCU--aAGa---------------GAUcaGGC-----UgGCccaacg-guCGCuuu-GCCa-CUCGUG---cgacu-GCgGgugaga-----------------------------------------------ugaaacGAG--gGGC-uaaacc-uguagau--gucug----cggucua------gcuuucuggac-GGGGGuucgacuCCCCC-caucuccacca
Ther.ther.         GGGGGUGaa-acggucu-cgaCGGGG-GUCgccga-gGGCGu----GGCU---GCGCGCCGAGGu----GCGGGUg------gCCUCGuaaaa-------ACCCGCaac-----------------ggcauaacugccaacaccaacuac---------------------------------------------------------gcucUCGCGG----cuuaauga-------------------------------CCGC--GA--------------------------------------------CCUCGCc--------CGGU--A-GCCcu---------GCCGGG-GGC-U-------------------------------------cACCGga--------------aGCGGGGacac------aaaCCCGGCua--------------------------------------GCCCGGG--GccAc----GCCCUC---UaaC---CCCGGGCgaagcu--ugaaGGGGGCuc---gCUCCUGGC---cGCCc---GUCC-GCGGGCcaaGCCAG-GAGgacac-------------------------------------------------gcgaaaCGC--GGAC-uacGCG-CGUaga---GGCC-----CGCCgua----gaGACCUUCGgac-GGGGGuucgacuCCCCC-CACCUCCacca
Dein.radi.         GGGGGUGac-ccgguuu-cgaCAGGG-Gaacugaa--GGUG-----aUGUu--GCGUGUCGAGGu----GCCGUUg------gCCUCGuaaaca------AACGGCaaagc---------------cauuuaacuggcaaccagaacuac---------------------------------------------------------gcUCUCGCUg----cuua------------------------------------AGU--GAGA------------------------------------------UGacGAC--------CGUGc-A-GCCcg---------GCCUUU-GGCgU--------------------------------------CGCGgaa-------------GUCacUAaa--------aaaGAAGGCua--------------------------------------GCCca----GGCga--uuCUCCAUA---GCC----gacGGCgaaacu----uUAUGGAGcuacGGCCu-GCGa--gaACCu---gCCC-ACUGGUga-gCGC--cGGCCcgacaa-u---------------------------------------------caaacAGU--GGGa-uacACA-CGUagac--GCA------CGCUgga----cggaCCUUUGgac-GGCGGuucgacuCCGCC-CACCUCCacca
Porp.ging.         GGGGCUGa--ccggcuu-ugaCA-GC-GUGaugaa--gCGGU----AUGUaa-GCAUGUAGUGCgu--gGGUGgCU------uGCACUauaau---cucAGaCAUCaaa-----------------aguuuaauuggcgaaaauaa---------------------------------------------------------cuacGcUCUcGCUG----cguaau--------------------------cgaagaaUAGU---AGAuUa--gacgcuucaucgccgccaaaguggcagcgacgaga--CAuCGCC--------CGA--GCaGCUuuu--------UCCCGA-AGUaGC--------------------------------------UCGauggugc---------GGUGcUG--------acaaaUCGGGAacc-------------------------------------GCUA-----CAGGa--ugCUUCC-U-gCCUG------UGGUcagauc---gaAcGGAAGaua-agGAuCGuGCa-uuGGGuc-gUUUC-AGcCUCc--GCuCG-cUCacgaaaa------------------------------------------------uuccaaCU--GAAAcuaaACA-UGUagaaa-GCAU----AUUGauu------cCAUGUuUGgac-GAGGGuucaauuCCCUC-CAGCUCCacca
Bact.frag.         GGGGUUGa--cugguuu-ugaCA-GC-GGGcagaa--AUgGU----GGGUaa-GCAUGCAGUGCgu--cGGUGAUU------uGCACUuuaau---cucAGUUAUCaaa-----------------auuuuaucuggcgaaacuaauua---------------------------------------------------------cGcUCUuGCUG----cuuaau--------------------------cgaaucaUAGU---AGAuUa-----gcuuaauccaggcaccaggugccaggacgaga--CauCACU--------CGG--AA-GCUguug-------CUCCGA-AGCaUU--------------------------------------CCGguucagu---------GGUGcaG--------uuacaUCGGGGaua-------------------------------------GUCA-----GAAGU--ggCCUUgc--GCUUC------UGAUgaaac-----uuuaAAGGaua-agGCAGGAaUu-gauGG----CUUU-GgUUCu---GcUCC-UGCacgaaaaa-----------------------------------------------cuu-aGgC--AAAGauaaGCA-UGUagaaa-GCUU----AUgAUuu------cCUCGUuUGgac-GAGGGuucgaauCCCUC-CAGCUCCacca
Bact.fors.         GGGGCUGa--ccgguuu-ugaCA-GC-GGGcagaa--gUGGU----UUGUaa-GCAUGCAGUGCgu--cGUUGGCC------gGCACUuuaau---cucGGUUGAUcca-----------------auuuuaacuggcgaaaauaacua---------------------------------------------------------CGcUCUcGCUG----cuuaau--------------------------cgaagcaCAGU---AGAuUG---aagcuuaauccuuacacaagguguuuggacgaga--CauCACC--------CGG--Aa-GCCgugg-------CUUCGA-GGCaaU--------------------------------------CCGaaccggu---------GGUGcaGa--------aaugUCGGAGaua-------------------------------------GCUUGa---CGgAU---gUCCC----GUaCG----gCAAGUgaaaa-----uucaGGGAaua-agGUUGCGGUg-gguGG----CUUC-GgUCUu---GCCGC-AGCacgaaaa-------------------------------------------------ugaaGgC--GAAGauaaGCA-UGUagaaa-GCAA----AUUAguu------cCUCGUuUGgac-GAGAGuucgaauCUCUC-CAGCUCCacca
Tann.fors.         GGGGCUGa--ccgguuu-ugaCA-GC-GGGcagaa--gUGGU----UUGUaa-GcaUGCAGUGCgu--cGUUGGCC------gGCACUuuaau---cucGGUUGAUccaa-----------------uuuuaacuggcgaaaauaacua---------------------------------------------------------CGcUCUcGCUG----cuuaau--------------------------cgaagcaCAGU---AGAuUG---aagcuuaauccuuacacaagguguuuggacgaga--CauCACC--------CGG--Aa-GCCgugg-------CUUCGA-GGCaaU--------------------------------------CCGaaccggu---------GGUGcaGa--------aaugUCGGAGaua-------------------------------------GCUUGa---CGgAU---gUCCC----GUaCG----gCAAGUgaaaa-----uucaGGGAaua-agGUUGCGGUg-gguGG----CUUC-GgUCUu---GCCGC-AGCacgaaaa-------------------------------------------------ugaaGgC--GAAGauaaGCA-wrUagaaa-GCAA----AUUAguu------cCUCGUuUGgac-GAGAGuucgaauCUCUC-CAGCUCCacca
Prev.inte.         GGGCUUGu--auggauu-ugaCG-GC-AAGacgaa--AUgGU----AUGUaa-GCAUGCGGAGCau--uGGUAGCU------gGCUCCuaaau---aucGGUUAUCaaa-----------------caauuaauuggcgaaaacaauua---------------------------------------------------------cGcUCUcGCUG----ccuaau--------------------------cgaaguaCAGU---AGAuUa--cuggcuuuauucgacuauuagauaguugaacgaga--CacUGCU---------CCa-aa-GAUguug-------UUCCGA-AUCgga--------------------------------------GGguaa-agc---------GGUGcaGg-------uuaaaUCGGAAaua-------------------------------------GUUUGG---GUA-U---gCCUC----GcUGC----CCAAAUgaaa------uuuaGAGGaua-aGGCAUUGGUu-gguGG----UUCA-GgUCUu---GCCAA-UGCUcgaaaa------------------------------------------------ccaaaGgC--UGAAauaaGCA-UGUagaaa-GCGU----AUgGUuu------cCUUGUgCGgac-CGGAGuucgacuCUCCG-CAGGUCCacca
Cyto.hutc.         GGGGUCGa--caggaau-ugaCAGGuuGAG-ugau-uAC-GU----GuGUaa-GCAUGCAGGCUc----aUGGGAUga----gAGCCUuaaaag--auaGUUCCGa--------------------acaauaauuggcgaagaaucuua---------------------------------------------------------cGccAUGGCUg----cuuaauua------------------------cgaacua-AGU-uUGUaaUaaugagcguugaguggagcuguuaagaacagcacccggccACAUCUC---------aCA-CGGCUU-cug-------UUCUGc-GGGCUG-------------------------------------UGaacu--------------GAGAUGUcgaacagca-ggacUAGGGga--------------------------------------GUac------AAGU---ugcGggU--GCUUc--------GCgaaa------uuAagUaacua-aGGUUaUAGUu-g-GGU----GCCG-GUGACCu--GCUAc-AGCUcgaaaa-------------------------------------------------ugaaCAUc-CGGC-uaaGCA-UGUagaag-AUuC---gAUaGUgg------cCUUcUCUGgac-GAGAGuucgaauCUCUC-CGACUCCacca
Chlo.tepi.         GGGGAUGa--caggcuaucgaCAGGA-UAggugug-aGAUGU---cGUU----GCACUCCGAGUuucagCAUGGACg-----gACUCGuuaaac---aaGUCUAUGua------------------ccaauagaugcagacgauuauucguau------------------------------------------------------GcAAUgGCUg----ccugauua---------------------------gcacaAGU-uaAUU-Cagaagccaucguccug-----------------cggugaaUGcGCUUac------UcUGAA-GCCGcc------------Gga-UGGCaUaacccgcgc---------------------------UUGaGccu--a-c--------GGGUuCGcg---------caagUaagc---------------------------------------UCCGUACA-UUCaU----gCCCGA--GgGGG--UGUGCGGGuaacc-----aaUCGGG-aua-agGGgaCGAa---cgcUg---CUGGcGGUGuaa---UCGg-aCCacgaaaaa-------------------------------------------------ccaACCa-CCAGagauGAG-UGUgg----uAACu---GCAUCgag----cagUGUCCUGgac-GCGGGuucaaguCCCGC-CAUCUCCacca
Chla.psit.         GGGGGUGua-uagguuu-cgaCUUGG-aAAugaag--UGUU-----AAUU---GCAUGCGGAGGgc---GUUGGCUg-----gCCUCCuaaaa------AGCCGACaaa-----------------acaauaaaugccgaaccuaaggcugaaugcgaaauuauca------------------guuucucugaccucaccgaggAaAgAuUAGCUg----cuuaauua---------------------------gcaaaAGU--UGuUgUcUagauaaccucua--------------------gauaaccCGGUGc--------UCAUA--GACUcc---------ACCAGA-GGUU--------------------------------------UGUGA-------a--------aCACCGuca-------ucuaUCUGGUug--------------------------------------GAAUcuAC-CUUCU-cuaAUUCUCA-AGGAG--GUuuAUUCgagau---uauUGAGAGU----CGUUGGCUUC--caUAGau-gUUCU-UAGCUGa-gGAAGUuCAAUGuaug--------------------------------------------------auuaCUA--GGGAcuaaGCA-UGUaga---GGUU-----AGCAggg---aguUUaCUAAGgac-GAGAGuucgaguCUCUC-CACCUCCacca
Chla.pneu.         GGGGGUGua-uagguuu-cgaCUUGa-AAAugaag--UGUU-----AAUU---GCAUGCGGAGGgc---GUUGGCUg-----gCCUCCuaaaa------AGCCAACaaa-----------------acaauaaaugccgaaccuaaggcugaaugcgaaauuauua------------------gcuuguuugacucaguagaggAaagAcUAGCUg----cuuaauua---------------------------gcaaaAGU--UGuUagcUagauaaucucua--------------------gguaaccCGGUAU--------cUGCG--AGCUcc---------ACCAGA-GGCU--------------------------------------UGCAa-------a--------AUACCGuca-------uuuaUCUGGUug--------------------------------------GAACuuAC-UUUCU-cuaAUUCUCA-AGGAA--GUucGUUCgagau---uuuUGAGAGU----CAUUGGCUgC--uaUAGag-gCUUC-UAGCUAa-gGgAGUcCAAUGuaaac-------------------------------------------------aauuCUA--GAAGauaaGCA-UGUaga---GGUU-----AGCAggg---ag-UUUgUAAGgac-GAGAGuucgaguCUCUC-CACCUCCacca
Chla.abor.         GGGGGUGua-uagguuu-cgaCUUAG-gAAugaag--UGUU-----AAUc---GCAUGCGGAGGgc---GUUGGCUg-----gCCUCCuaaaa------AGCCGACaaa-----------------acaauaaaugccgaaccuaaggcuaaaugcgaaauuauca------------------guuucucugaacucucugagcAAAgAuUAGCUg----cuuaauua---------------------------gcaaaAGU--UGuUaUUUagauaaccucua--------------------gauaaccCGGUcU--------UCAUG--GACUcu---------ACCAGA-GGUU--------------------------------------UGUGAa---------------GuACCGuca-------ucuaUCUGGUug--------------------------------------GAAUcuAC-CUCCU-cuaAUUCUCA-AGGGG--GUuuAUUCgagau---uauUGAGAAU----CGUUGGUUUC--caUAGau-gUUCU-UAGCUGa-gGAAAUgCAACGuuaga--------------------------------------------------cuaCUA--GGGAcuaaGCA-UGUaga---aGUU-----AGCAggg---aguUUaCUAAGgac-GAGAGuucgaguCUCUC-CACCUCCacca
Chla.trac.         GGGGGUGua-aagguuu-cgaCUUAG-aAAugaag--CGUU-----AAUU---GCAUGCGGAGGgc---GUUGGCUg-----gCCUCCuaaaa------AGCCGACaaa-----------------acaauaaaugccgaaccuaaggcugaaugcgaaauuauca------------------gcuucgcugaucucgaagaucuaagAGUAGCUg----cuuaauua----------------------------gcaaAGU--UGUUaccuaaau-acg------------------------ggugaccCGGUGU--------UCGcG--AGCUcc---------ACCAGA-GGUU--------------------------------------UuCGAa---------------ACACCGuca-------uguaUCUGGUua--------------------------------------GAACUUAg-gUCCU-uuaAUUCUCG-AGGAa--aUGAGUUUgaaau--uuaaUGAGAGU----CGUUAGUCUC--uaUAGgg-gUUUC-UAGCUGa-gGAGACaUAACGuauag--------------------------------------------------uacCUA--GGAAcuaaGCA-UGUaga---GGUU-----AGCGggg---aguUUaCUAAGgac-GAGAGuucgacuCUCUC-CACCUCCacca
Chla.muri.         GGGGGUGua-aagguuu-cgaCUUAG-aAAugaag--CGUU-----AAUU---GCAUGCGGAGGgc---GUUGGCUg-----gCCUCCuaaaa------AGCCGACaaa-----------------acaauaaaugccgaaccuaaggcugaaugcgaaauuauca------------------gcuucgcugaucuuaaugaucuaagAGUuGCUg----cuuaauua----------------------------gcaaAGU--uGUUaccuaagu-acu------------------------gguaaccCGGUGU--------UCGcG--AGCUcc---------ACCAGA-GGUU--------------------------------------UuCGAa---------------ACGCCGuca-------uuuaUCUGGUua--------------------------------------GAAUUAGg-gCCUU-uuaACUCUCA-AGGGa--aCUAAUUUgaauu--uuaaUGAGAGU----CGUUGGUCUC--uaUAGag-gUUUC-UAGCUGa-gGAGAUaUAACGuaaaa-------------------------------------------------uauuCUA--GAAAcuaaGCA-UGUaga---GGUU-----AGCGggg---aguUUaCUAAGgac-GAGAGuucgaauCUCUC-CACCUCCacca
Osci.spec.         nnnnnnnnn-nnnnnnn-nnnnAGUG-GAGugaaa--GCUgc---uCCGUgauuCAGGCCGAGAg----UGAGUUGu-----cUCUCGucaaucc---cCAACUCAaaaaa---------------aaaguaaaugcgaacaacaucguuccuuuc------------------------------------gcucguaaagcgGcuCCUguaGCUG----ccuaa---------------------------aaaccucUAGC-----AGGuuUgagc--------------------------------gGUCUaUA--------GCUU---gac------------UCCGUU-aa-----------------------------------------AGGC----------------UAcAGACaaac-----ccucAACGGAugcu------------------------------------CUAGUAAg---GUu-u--CUCuGGUU-AC-----UUGCUAGuuaaga-cucAACCuGAGu----------------------------------------------------AUC-CUguCAUCCGGGAUaauGGAUGGUCCccGCCCAgaGGGU-uaaaUGGGC------------uacGCC-UGugaaugaGCGGu----aGGCgaau---acCUCUGCUacac-agcannnnnnnnnnnnn-nnnnnnnnnnn
Anab.spec.         GGGUCcGu---cgguuu-cgaCAGGU-UGGcgaac--GCUaC---UCUGUgauuCAGGUCgAGAg----UGAGUCUc-----cUCU-Gcaaauca----AGGCUCAaaaca---------------aaaguaaaugcgaauaacaucguuaaauuu------------------------------------gcucguaaggacgCUcuAGuAGCUG----ccuaa---------------------------------aUAGC-cUcUUucAG-guucgagc-----------------------------GUCUUCG--------GUUU---gac------------UCCGUU-aa-----------------------------------------GGAC----------------UGAAGACcaac-----ccccAACGGAugcu------------------------------------CUAGCAAu---GUU----CUCUGGUUGGC-----UUGCUAGcuaaga-uuuAAUCAGAGc----------------------------------------------------AUC-CUa-CGUUCGGGAUaauGAACGAUUCccGCCUUgaGGGU-cagaAAGGC------------uaaACC-UGugaaugaGCGGG---G-GGUcaau---acCCAAUUUGgac-AGCAGuucgacuCUGCU-C-GAUCCacca
Chro.spec.         nnnnnnnnn-nnnnnnn-nnnnAGGU-UGGcgaaa--GCuAa---UCUGUgauGCAGGUCGAGAg----UGAGUCgc-----cUCUCGgaaauac----aGGCUCAaaucua--------------aauauagaugcaaacaacaucguaaaauuc------------------------------------------gagcgucaAGCAGuaUUUG----cuuaa---------------------------gcccaacUGAG--gcUUGCUc--------------------------------------GcUUUUA--------GUCc---gac------------UCCGUU-aa-----------------------------------------cGGC----------------UAAAA-Cgaaa-----ccccAACGGAugca------------------------------------cCuuUUA----GUU-ac-UUUCGGUuGAC-----UAAuuGcguaaagacuucACCGAAAc----------------------------------------------------AUC-CUacUAUCAGGGAUaauUGAUAGUUCcaGUUCUugGGAU-uauaAGAAC------------uaaACC-UGUgaaugaGCAGA---aU-GUcauu---auCCAGUCUggac-agcannnnnnnnnnnnn-nnnnnnnnnnn
Nost.punc.         GGGUCCGuc-acgguuu-cgaCAGGU-UGGcgaac--GCUgC---UCUGUgauuCAGGUCGAGAg----UGAGUCUc-----cUCUCGgaaauca---aAGGCUCAaacaa---------------aaaguaaaugcgaauaacaucguuaacuuu------------------------------------gcucguaaggauGCUCuAGUagcUG----ccuaaa--------------------------caccucuUAua--gGUUcGAGC-------------------------------------GUCUUUA--------GUUU---gac------------UCCGUU-aa-----------------------------------------GGAC----------------UGAAGACcaaa-----ccccAACGGAugcu------------------------------------CUAGUAAg--cGUU----CUCUGGUUGGC-----UUGCUAGcuaaga-uuaAAUCAGAGa----------------------------------------------------AUC-CUa-CGUUCGGGAUaauGAACGAUUCccGCCUUgaGGGU-cagaAAGGC------------uaaACC-UGugaaugaGCGGG---G-GGUcaau---acCCAAUUUGgac-AGCAGuucgacuCUGCU-CGGAUCCacca
Frem.dipl.         nnnnnnnnn-nnnnnnn-nnnnAGGU-UGGcgaac--GCUgC---UCUGUgauuCAGGUCGAGAg----UGAGUCUc-----cUCUCGcaaaucc---aAGGCUCAaaaca---------------aaaguaaaugcgaacaauaucguaaaauuu------------------------------------gcucguaaggaaGCcCuaGUUgcUG----ccuaauag--------------------------ccucuUAua--GGUccGaGC-------------------------------------GUCUUUA--------GUCC---gac------------UCCGUU-aa-----------------------------------------GGGC----------------UAGAGACcaa------ccccAACGGAugcu------------------------------------CUAGCAAG--uaUU----CUCUGGUUGA-----CUUGCUAGcuaaga-cuuAAUCAGAGc----------------------------------------------------AUC-CUa-CGUUCGGGAUaauGAACGAUUCccGCCUugaGGGU-cagauAGGC------------uaaACC-UGugaaugaGCGGA---G-GGUcaau---acCCAAUUUggac-agcannnnnnnnnnnnn-nnnnnnnnnnn
Plec.bory.         nnnnnnnnn-nnnnnnn-nnnnAGGU-UGGcgaac--GUUCc---AUCGAgauGCAGGUCGAGAg----gGAGUCca-----cUCUCGuaaaucu----aGGCUCaaaaaa------------------auagaugcaaacaacaucguaccuuuc------------------------------------gcucguaaaacuGCaCcuGUUgcAG----cauaa-----------------------aacaccucuaaUUca--GGUucGaGC-------------------------------------GCUUACc--------GUCU---gac------------ACCGUU-aa-----------------------------------------AGAU----------------aGUAAGCacaa-----ccccAACGGUugcu------------------------------------CUAGAAUu-ucGCC-----UUUGGUcGGC-----AUUCUAGcuaaga-caauACCAAAgc----------------------------------------------------aUC-CUauUGUCCGGGAcaaaGGACAGUUCccGCUUCgaGGAU-uagaGAAGC------------uaaACC-UGUgaaugaUUGAU---aGAGCuaau---acCCAGUUUggac-acggnnnnnnnnnnnnn-nnnnnnnnnnn
Tric.eryt.         GGGGCAUua-acgguuu-cgaCAGGG-UGGugaa---AUCUgc--uUUGUgauGCAGGcCGAGAg----UGAGUCUu-----cUCUCGaaaauc---cuGGACUCAaaaa----------------auaauaagugcaaacaacauuguaccuuuu------------------------------------gcucguaagcaaGUagcuGCUcuAG----cuuaa--------------------uugcaaaaacaccuCUuauaGGUucgaGC-------------------------------------GUCUGUG--------GUUu---gac------------UCCGUU-aa-----------------------------------------uAAU----------------UGCAGACuuaa-----ccccAACGGAugcu------------------------------------CUAGCaau-uuuuc-----ucugguuaccu------GCUAGuuaaga-uuuaaccagagc----------------------------------------------------AUC-CCacUAUCCGGGAUaauGGAUAGUCCcaGCUCUgaGGGUuaagaAGGGC------------uaaaCC-UGUgaaugaGUGA----aGGGUgaau---agCCAUUUUGgac-AUGGGuucgacuCCCAU-AUGCUCCacca
Syne.spec.-1.      GGGGCCGca-augguuu-cgaCAGGU-UGGcgaaa--GCUUgc---CCGUgauACAGGUCGAGAg----UGAGUCUc-----cUCUCGcaaauca---aAGGCUCAaaaa----------------aaaguaacugcgaauaacaucgucagcuuc------------------------------------------aaacggGUaGcCaUaGCAG----ccuagucuguaa--------------aagcuacauuuucUUGU-caAaGaCcGUuu-----------------------------------ACUUCUu--------UUCU---gac------------UCCGUU-a------------------------------------------AGGAu---------------uAGAGGUuaa------ccccAACGGAugcu------------------------------------UUGUU----UGGCU-cuuCUCUAGUUAGCUA-----AACAAucaagacucaGACUAGAGc----------------------------------------------------AUC-CCacCAUCaGGGAUaaucGAUGGuCCccGUCCUagGGcU--agaAGGAC------------uaaACC-UGUgaaugaGCGG----aAAGUuaau---acCCAGUUUGgac-AGCAGuucaauuCUGCU-CGGCUCCacca
Syne.spec.-2.      GGGGCUGua-augguuu-cgaCGUGU-UGGugaau--cCUUca---CCGUgauuCAGGCCGAGAg----gGAGUCca-----cUCUCGuaaaucc----aGGCUCaacca----------------aaaguaacugcgaacaacaucguuccuuuc------------------------------------gcucguaaggcugcuCcUGuAGCUG----cuuaa-------------------acgccacaaacuuucUGGC--U-CGaGc---------------------------------------GUCUAGu---------CGUA--gac------------UCCGUU-aa----------------------------------------UACG-----------------cCUAGACuuaaac---ccccAACGGAugcu------------------------------------CGAGu----GGCGG---cCUCAGGU-CCGUC-c---uCUCGcuaag-caaaaACCUGAGc----------------------------------------------------AUC-CCgcCAACGGGGAUaauCGUUGGCUCccGCACaguGGGU-caaccGUGC------------uaaGCC-UGugaacgaGCGG----aAAGuuacu---agUCAAUGCGgac-AGCGGuucgauuCCGCU-CAGCUCCacca
Syne.spec.-3.      GGGGCUGca-augguuu-cgaCGGGG-CAUga----gGaGGG---UgACU--------gaaGCC-------UGCUC-------GGUca-----------GAGCGaa--------------------cccguaacagcgaacaacaucguucguuuc------------------------------------ucccgucaagccgcCCcCGUggCUG----ccugaccucuu--------------------aacggagaCGG---gGUGaGG---------------------------------------UCAGcCU--------ugUCAcccaa------------auGaCC-ca----------------------------------------UGG------------------GGcCUGG--aa---------GGgCccuucccauacua------------------------------------------------------------------------------------------------------------------------------------------------------------------------------------------------------------------------------GGUgG---UCCaCac-----caGUGUCUCGgac-AGCGGuucgauuCCGCU-CAGCUCCacca
Syne.spec.-4.      GGGGCUGca-augguuu-cgaCGGGG-CAUga----gGaGGG---UgACU--------gaaGCC-------UGCUC-------GGUga-----------GAGCAaa--------------------cccguaacugcgaacaacaucguucguuuc------------------------------------ucccgucacgcagcCccUGUUgCUG----ccugacccuu---------------------aggggagaUGG---GGUGaaG---------------------------------------UCAGCCU--------uaUCAcccaa------------auGaCU-ca----------------------------------------UGG------------------GGGCUGG--aa---------GGcCccucaaaccacua------------------------------------------------------------------------------------------------------------------------------------------------------------------------------------------------------------------------------GGUgA---UCCaCac-----caGUGUUUCGgac-AGCGGuucgauuCCGCU-CAGCUCCacca
Proc.mari.-2.      GGGGCUGca-augguuu-cgaCGGGG-UAUga----gGaGGG---UgACU--------gaaGCC-------UGCUC-------GGUaa-----------GAGCAaa--------------------uccguaacugcgaacaacaucguucguuuc------------------------------------ucccgucagccugcCCUCGUggCUG----ccugacccuaau-------------------aagggagaUGG---gGUGAGG---------------------------------------uCAGCCU--------uaUCAcccaa------------auGaCC-ca----------------------------------------UGG------------------GGGCUGc--ga---------GGcCccuuuaacuacua------------------------------------------------------------------------------------------------------------------------------------------------------------------------------------------------------------------------------GGUgA---UCCaCac-----caGUGUCUCGgac-AGCGGuucgauuCCGCU-CAGCUCCacca
Proc.mari.-1.      GGGGUUGua-augguuu-cgaCGGGG-CGUaa----ggaAGA---UgACU--------gaAGCC-------UGCUC-------GGUUa-----------GAGCAaa--------------------aacacaaacgcuaacaaaaucguuaguuuc------------------------------------ucccgucaaacagcaccaGUUgCUG----cuugaucuc----------------------aaaggagaUGG---GGUgaua---------------------------------------UCAGcCU--------uaUCaaccaa------------auGaUC-ca----------------------------------------aGG------------------AGcCUGG-aag---------GGcUccacca-uuacua------------------------------------------------------------------------------------------------------------------------------------------------------------------------------------------------------------------------------GGUgA---UCUcaac-----cgAUGUUUCGgac-AGCGGuucgauuCCGCU-CAACUCCacca
Syne.spec.-5.      GGGGCUGca-augguuu-cgaCGGGG-CAUga----ggaggg---ugacu--------gaaGCC-------UGCUC-------GGUaa-----------GAGCGaa--------------------cccguaacagcgaacaacaucguucguuuc------------------------------------ucccgucaagccgcCCcCGUggCUG----ccugacacucuu-------------------aacggagaCGG---gGUGaGG---------------------------------------ucagcCU--------ugUCAcccaa------------augacc-ca----------------------------------------UGG------------------GGnnnnn-nnn---------nnnnnnnnnn-nnnnnn------------------------------------------------------------------------------------------------------------------------------------------------------------------------------------------------------------------------------nnnnn---nnnn----------GUGUUUCGgac-AGCGGuucgauuCCGCU-CAGCUCCacca
Syne.spec.-6.      GGGGCUGca-augguuu-cgaCGGGG-CAUga----ggaggg---ugacu--------gaaGCC-------UGCUC-------GGUca-----------GAGCGaa--------------------cccguaacagcgaacaacaucguucguuuc------------------------------------ucccgucaagccgcCCcUGUggCUG----ccugacacucuu-------------------aacggagaCGG---gGUGaGG---------------------------------------ucagcCU--------ugUCAcccaa------------augacc-ca----------------------------------------UGG------------------GGnnnnn-nnn---------nnnnnnnnnn-nnnnnn------------------------------------------------------------------------------------------------------------------------------------------------------------------------------------------------------------------------------nnnnn---nnnn----------GUGUCUCGgac-AGCGGuucgauuCCGCU-CAGCUCCacca
Cyan.para.cy.      GGGGCUGuu-uagguuu-cgaCGUUU-UUUucu---aAUUAUg--UUUGUuaaGCAAGUCGAGGa----uUUgUUcu-----aUCUCGaaaau------cAAgAAcucucaa--------------aauuuaaacgcaacuaauauuguacgu---------------------------------------uuuaaccguaaagcAGcUUUcgCUG----uuuaauaauuacu--uuuaauuuaaaaaccuaauuuuuuUAG----GAAuUUauuu-----------------------------------AUUUAUU-------------GuuUAu-----------------ccUGcuU---------------------------------------------------------AAUGAAU-------------------------------------------------------------------------------------------------------------uaaaaaaagc---------------------------------------------------------------------------------------------------------------------uauACU-UGUg----aAUAAAcgcAUAAUu------uaAAAAAACGgac-GUGGGuucaaauCCCAC-CAGCUCCacca
Porp.purp.cl.      GGGGCUGc--aagguuu-cuaCAUUG-UGAaaaaa--CAAAu---AuAUGaaagUaAAAcGAGCu---caUUAUUag-----aGCUUuuagu------uaAAUAA----------------------------augcagaaaauaauauuauu--------------------------------------gcuuuuucucgaaaAuuAG-CUGUUG----cauaaauagucu--------caauuuuuguaauucgaagUGAU--AGaCUcuUauacac-----------------------------uacgaaUAUU------------cuGuUAG----------------agUUGcUc---------------------uu----------------------------------AAUAaaa-------------gaaaa------------------------------------------------------------------------------------------guaaaaaaaua--caaa-----------uucuuauGuUU-UUUaccu-----------------------------------------------------------------------GAAuuGAuU-caaUUU-aAgg-uuagUAU-U---uUUUGau-----uuUUACAAUGgac-GUGGGuucaaguCCCAC-CAGCUCCacca
Odon.sine.cl.      GGGGCUGac-uugguuu-cgaCAUUU-AAAaauu---GUUAc---AGUAUgauGCAGGUCGAAG------UUUCUaa-----uCUUCGuaaaaaa-----AGAGAaauuu----------------auaauaaaugcuaauaauuuaauuucuu---------cuguguuuaaaaguuuaucaacuaagcaaaauaguuuaaauuuaaguUUUGCUG----uuuaaguuuuaug------------cacauuuaaugaucUAGU--AAAuaacuuug--------------------------------uucGCUAUAA-------------uUUAUA---------------UU-UAUAAcu--------------AGACuuuu--------GUCUuuu-------------------UUAUAGU----------uuaGAauaac-----------------------------------------------------------------------------------------uuuaucauuu--caaa-----------ccuc---GUUCcAUc--u------------------------------------------------------------------------aGU-uGAAC-uaaACC-UGUgaacgaAUACU---aUAAUaaaa---uuUUUAGAUGgac-GUGGGuucgacuCCCAU-CAGCUCCacca
Pavl.luth.cl.pa.   ggggcuguu-uagguuu-cgauccua-aaaaaaac--uaaua---agugugaugcaaguAGAGG-----------aaa----cCCUCUuaaaaaa-----aaagcaa-------------------acaauaaaugcaaacaauauuuuaucuuuu--aaucgaguagcuguagccccguauaguuauauagcauuaacuccuuAUGGUCaaaAAAU----c-uagaaa-------------------------------GUUU-----GAUCGUaaagcaaa------------------------ccuuagcuagaa---------------GcaC-------------acaa-GaaUaa-------------------------------------------------------------------------------------------------------------------------------------------------------------------------------------------------------------------------------------------------------------------------------------------------------------------------------------------------------------------------------------------
Boli.spec.pl.      nnnnnnnnn-nnnnnnn-nnnnAUUA-UAAaaauuu-UAAAUu--aUGAU---GCAAGUCGAAA------aAUAUU------aUUUCGuuaaaa-----AAUAUaccuuu----------------aaaauaaaugcaaauaacauuuuagcuuuu---------------------------------------aaccguaaaUcUuuAAGUUUCG----cuuaa-------------------------aauuauuuaUGGA-aAUUUuuAaGagug--------------------------uccaucucUUAaAA----------a--UUUGaA-----------aAcUU--UaUGGA------------------------------------ca-------------------UUaUAAa-acuagacuuuucuAGaUc------------------------------------------------------------------------------------uuuuaaguuaaauuauuacuuaauuccuuu---UAAuu-uGUUC-AUUUUA------------------------------------------------------------------------AAU--GAAC-uaaACU-UGUgaacg-AUUA----AUUUAucac---uuUUAUAAUggac-guggnnnnnnnnnnnnn-nnnnnnnnnnn
Guil.thet.cl.      GGGGCUGau-uuggauu-cgaCAUau-aAAuuugcguGuUUC---AUUAUgaaGCAAGUCAAG------UUUAAUGa------uCUUGuaaaaaa----CAUUAAAgua-----------------caaauaaaugcaagcaauauaguuucauuua------------------------------------guucaaaacguUUAGUcUcUUUUG----cauaa---------------------------------gCAAA--AuGuGUUAAuaac---------------------------uuucuuAGUAgAA----------A--UUGGaG-----------aAGUU--UaCUAA------------------------------------gaU------------------UUaUAUU------uacuccauAAUUa------------------------------------------------------------------------------------uuuuaaagauggua-------------------aAA----AGGU-G-AUUcaucauu------------------------------------------------------------------UgUauGUUUcuaaACUuUGUgaaagaAUAGU---GGGcUcc-----auUUauaAUGaac-GUGGGuucaaauCCCAC-CAGCUCCacca
Thal.weis.cl.      GGGGCUGau-uugguuu-cgaCAUUU-AAAacuu---CUUucua-UgUGU----CAGGUCAAAGuu---uGUAUU--------CUUUGuaaaaa------AAUACuaaaaua--------------cuaauaaaugcuaauaauauaauaccguuua---------uuuuuaaagcaguaaaaacaaaaaaagaagcaauggcuuuAAAUuUUGcUG----uauag-------------------uucauuaacuuagguUAuU-aAAuAUUU---------------------------------uuuc--AUUAUAA-------------CUGGAc------------UuUUC--UCUAG--------------------------------------u------------------UUAUAGU----------uuaGAAuAaa------------------------------------------------------------------------------------uuuaaauuuugcaaa-----------------AcUc---GUUC-GAAAaUu-----------------------------------------------------------------------UUC--GGGC-uaaACC-UGuaa--acGCAaA-uacuAAGaa-----auUUUAGAUGgac-AUGGGuucaauuCCCAU-CAGUUCCacca
Meso.viri.cl.      GGGGUUGaa-augguuu-cgaCGUUU-UAGuaua---uAUCU---UCUUacaaGCAAGUCGAGAa----UGAACca------aUCUCGuaaacuu----gaGUUCAauug----------------caaauaaaugcuaacaauauuuuaccuuuua---------------------------------------aucguaagacaGccGUaGcUG----uuuaa--------------------------aaguuuuuUGaU--aACuuCg-------------------------------agcauuuuUgAUUUG--------uUAAA-uacaa-----------UUGUUau---------------------aaaacaaauuaaauaauaguuUUUGuuucuuu-a--------CAAAUaAug--------uagaAAUAAuuuuu--------------------------------------------------------------------------------uuuagcuuuucaag-------------------CUcc-aGCUU-UGUGGauua---------------------------------------------------------------------GCA--AAGC-uaaACU-UGUgaauacaAAGA-agAGAUca------aaCUAAAGCGgac-GCGGGuucaaaaCCCGC-CAGCUCCacca
Recl.amer.mt.      GGGGAUGuuuuuaguauucgaCAUAG-UgGaa-----UUUAUuuguGGAAuuuUUAAAUuuuAuu----UUUUUaUcu----acUuu-uaa--------G-AAGGAuuuuuu---aaaaacaccuuguuaaauaaguaaauaaaaaaaa--------------------------------------------------------------------------------------------------------------------------------------------------------------------------------------------------------------------------------------------------------------------------------------------------------------------------------------------------------------------------------------------------------------------------------------------------------------------------------------------------------------------------------uaaaAUU-UAAau--uaUUUUu-uuAUAAG-------aaUgACUAUGgaa-CCGAGggcggauCUCGG-CAUCUCCacca
Fibb.succ.         GGGGGUGcu-cugguuu-cgaCAGGG-UUAccgaa--GUgUU--aguUGCaaguCGAGgucUcAg---acgAGGgCaa----cUcGuuaaaaa------G-UCUgaa-----------------aaaaaauaagugcugacgaaaac---------------------------------------------------------uacgcacUCGcUG----ccuaa---------------------------------uUAaC--GGcaa----------------------------------------cgCCGGGCc---u---CAUUC----CGc----------UCCCAU-CG----------------------------------------GGAUGuac-------------GUCCGGacg-------caauAUGGGAua--------------------------------------gGGaAGugucAUGC----CUGG--gGGCAU-----CUcCCg--agauuuacUaggCUGG-ucaaacUCCGCGC----Cga-cc-UUCU--UG--G---GCGUG-GAuaaga-cgagauc-----------------------------------------uuaaa--UAcgaAGGGaac-aCU-UGuaggaacGUAc--auGG-ACg--------UGAUUUUGgac-AGGGGuucgacuCCCCU-CACCUCCacca
Trep.dent.         GGGGAUGuu-ccgguuu-cgaCCGGA-AAGacgga--GGcUU----AAGCu--GCAGGCGGAG------UGCCGA--------uCUCCuga--------uUCGGCAaa-----------------cacuauaacugccgaaaauaacgacaguu---------------------------------------------ucgauuacgccuUaGCuG----cauaa----------------------------------UcGC---G--------------------------------------------GAAUCug--------CUUUG--caCU-----------GCUCCG-AG----------------------------------------CGGGG-------c--------auGAUUCcg---acgccaauCGGGGCuu--------------------------------------gCUUUUUagcGGCG-----UGUA-AACGUU----AAAAGGg---acuuuu-UU-UACAc-uaaGGAUUCGacG----cUu----UCGG-UGCuGc---UacCG-GAUCCcuacaa---------------------------------------------ccaccucGCA--CCGA-uaaGCC-UGUa--gacGCUU--cuGAuUCc--------CUUUUCGGgac-GGGGGuucaauuCCCCC-CAUCUCCacca
Trep.pall.         GGGGAUGac-uagguuu-cgaCUAGG-GAuguggg--GUGUU----GCGCu--GCAGGUGGAG------UGUCGA--------uCUCCuga--------uUCGGCGcc------------------uuuauaacugccaauucugacaguuuc------------------------------------------------gacuacgcgcUcGCcG----cguaa----------------------------------UcGC---G--------------------------------------------GGCCuGU--------GUUUG--cgCU-----------GCUCUG-AG----------------------------------------CGAAC-----a-u--------AUcGGCCcg---acgccaaaCGGAGCuu--------------------------------------gCUCUUACG--UUG-----UGCAcGGCGGa--CGUAGGGGg---acuuuugUC-UGUGc-uaaGACUCUGgcG----CgU----GCGG-UGCAgGcc-UagCA-GAGUCcgacaaa-------------------------------------------cgcaguacGCA--CCGC-uaaACC-UGUa--ggcGCGC----AGCACu------cgcUCUUUAGgac-GGGGGuucgauuCCCCC-CAUCUCCacca
Borr.burg.         GGGGAUGuu-uuggauu-ugaCUGAA-AAUguuaa--UAUUG---UAAGUu--GCAGGCAGAGG---------GAA-------UCUCUuaaaa-----cUUCu-----------------------aaaauaaaugcaaaaaauaauaacuuu---------------------------------------acaagcucaaaucuuguaaugGCUG----cuuaa--------------------------------guUAGC------------------------------------------------AGAGGGUu---uu--GuUGA--AUUUg----------GCUUUG-AGGU--------------------------------------UCAcU----ua-u--------ACUCUUUucg------acauCAAAGCuu--------------------------------------gCUUAAAAauGUUu-----UCAAGU-uGAU---UUUUAGGg---acuuuuauACUUGA---gaGCAAUuUGGU----GGuuu-gCUAG-UAUuUCcaaACCAu-AUUGCuu--------------------------------------------------aauaaaAUA--CUAGauaaGCU-UGUa--gaaGCUUA---UAGUA-------uuAUUUUUAGgac-GCGGGuucaauuCCCGC-CAUCUCCacca
Rhod.palu.         GGGGGCGaaauaggau--cgaCGAG--GGcguaaa--gGGCu---cGCUu----UUUCCcGGUau----UGuUCCG------ccGUUau----------CGGGcUAcu------------------gcaauaguugccaacgacaacuau------------------------------------------------gcuccgGUUgcUCAGGCUG----cguaa--------------------------------cgCAGU--UUGAaaGAC-------------------------------------------------cacUUUAA-agcCc-----------UAACGG--G----------------------------------------UUAAG----------------------------------cuCCGUUA---------------------------------------------gGCgGGGUuc---GGAGGC---ACCU-gGCaacagaa-----------GCCUCCa----------------cUc-----GUCG-GGg-cA------------------------------------------------------------------------aCC--CGAC-uauGGA-AAua---aauGGC----cGCCgcaau---aaaCCaUUCGgac-CCGGGggcgguaCCCGG-CGCCUCCacca
Magn.magn.-1.      GGGGGCGaaauaggau--cgaCGA-G-GGcguaaa--gGGGG----AGCuu---UCGCUcGGCau----GGuUCCG------ccGUUau----------CGGGcCUuu------------------gcaauaguugccaacgacaacuuu------------------------------------------------gcuccgGUggcUGuCGCCG----cguaa---------------------------------gCGGU--GcCAaaaAC------------------------------------------------cga-cCUAa-aguCC-----------UA-GCG-GG-----------------------------------------UAGc----------------------------------acCGCaUA---------------------------------------------gGCgGGGUuc---GGAGGU---ACCU-gGCaacagaa-----------ACCUCCa----------------cUc-----GCCG-GUCgcAa-----------------------------------------------------------------------GGC--CGGC-uauGGC-GAua---aacGUU----CUUCgcaau---aaaCCUaUCGgac-CCGGGggcgguaCCCGG-CGCCUCCacca
Magn.magn.-2.      GGGGGCGaaauaggau--cgaCGUG--CGcaguaaa-gGUcU----GGUuu---GCGUUcGGCau----GAUaCCa------ccGUUau----------cGG-GUCaauc----------------cuaacaagugccaacgacaac---------------------------------------------------guugaacuugccgcUGCGG----cuuag---------------------------------uCCGU-aA--------------------------------------------------------------------------------------------------------------------------------------------------------------------------------------------------------------------------------GCgCGGUcc---GGGGGGA--ACCG-gGCaacagaac---------UCCCCCCa----------------cUU-----GCCG-Ggc-AA--------------------------------------------------------------------------C--CGGC-uauGAC-GCua---aacGCC----AcACggaau---agaCGuUACGgac-CCGGGggcgguaCCCGG-CGCCUCCacca
Rhod.rubr.         GGGGGCGaaauaggau--cgaCACG--CGUgguaaa-gAUAA----AGGuu---GUGUUcGGCau---gGuaUCCa------ccGUUau----------cGGG-cCaacu----------------cugacaagugccaacgacaacguu---------------------------------------------------gaacuugccgcUGCGG----ccuaa------------------------------acacCCGU-aA--------------------------------------------------------------------------------------------------------------------------------------------------------------------------------------------------------------------------------GCgCGGUuc---GGGGGGGc-ACCG-gGCaacagaag---------CCCCCCCa----------------cuU-----GCCG-GcuuGu--------------------------------------------------------------------------C--CGGC-uauGAC-GCua---aacCCU----UUAUggaau---aaGCGuUGUGgac-CCGGGggcgguaCCCGG-CGCCUCCacca
Meth.exto.pa.      GGGGGCGaaauaggau--cga--------------------------------------------------------------------------------------------------------------------------------------------------------------------------------------------------------------------------------------------------------------------------------------------------------------------------------------------------------------------------------------------------------------------------------------------------------------------------------------------------------------------------------------------------------------------GCCG-GUCgcaa-----------------------------------------------------------------------GGC--CGGC-uauggc-gaua---aacguu----cuucgaaau---aaaccuaucggac-CCGGGggcgguaCCCGG-CGCCUCCacca
Brad.japo.         GGGGGCGaaauaggau--cgaCGAG--GGcguaaa--gGGCG---uGCUu----UUUCCcGGUau----uGUuCCG------ccGUUau----------CGG-GCuacu-----------------gcaauaguugccaacgacaacuuugcu------------------------------------------------ccggUUgcUCAGGCUG----cguaa--------------------------------cgCAGU--UUGAaaGA-------------------------------------------------ccauCUUAA-aguCc-----------UAACGG--G----------------------------------------UUAAG----------------------------------cuCCGUUA---------------------------------------------gGCgGGGUuc---GGAGGC---ACCU-gGCaacagaa-----------GCCUCCa----------------cuu-----GCCG-GUuc---------------------------------------------------------------------------GC--CGGC-uauGGA-AAua---aacGGU----CGUCguaau---aaaCCaUUCGgac-CCGGGggcgguaCCCGG-CGCCUCCacca
Meso.loti.         GGGGGCGaaauaggau--cgaCGAA--GGUguaaa--gAUCG---uaCUu----UUGCCcGGCau----uGUaCCa------ccGUCau----------cGG-GCuaaa-----------------cuuauaguugccaacgacaacuaugcg------------------------------------------------gaaGCacGUCuCGCUG----cuuaa--------------------------------ugCAGU--GcGAUa-GC-----------------------------------------------uucaaaUcAA--gccc-----------UAGGGG-------------------------------------------UUcGca-----------------------------------CUUCUA---------------------------------------------gGCgGGGUuc---GGAGGU---ACCU-gGCaacagaa-----------ACCUCCa----------------cuu-----GUCG-GcUu--------------------------------------------------------------------------GaC--CGAC-uauGGC-GAua---aauGGg----CGAUgaaau---aaGCCgUUCGgac-CCGGGggcgguaCCCGG-CGCCUCCacca
Bruc.meli.         GGGGGCGaaacaggau--cgaCAA-G-GGUguaaa--gAUCG---cUCUu----UUACUcGGCau----GAUaCCa------ccGUUau----------cGG-GUCaac-----------------agaguaguugcaaaugacaacaaugcu---------------------------------------------caggguUaUgcUcUCGCUG----ccuaa----------------------------------UGGC--GGuGcgG-G-------------------------------------------aaacccacucUaAA--gucc-----------UUAGGG-------------------------------------------UUaGcc-----------------------------------CCUUAA---------------------------------------------gGCgGGGUuc---GGAGGC---ACCU-gGCaacagaa-----------GCCUCCa----------------cUU-----GUCG-GCgcAA-------------------------------------------------------------------------GC--CGAC-uauGGU-GAua---aacGGA----CGGUguaau---aaACCCaUUGgac-CCGGGggcgguaCCCGG-CGCCUCCacca
Sino.meli.         GGGGGCGaaauaggau--cgaCAA-G-GGcguaaa--gAUCG---aCUUu----UUGCUcGGCau----uGUaCCa------ccGUUau----------cGG-GCuaaa-----------------cuuauaguugcaaacgacaacuaugcu------------------------------------------------gaaGcaCGUcUCGCUG----ccuaa----------------------------------CGGC--GGuGCGa-C---------------------------------------------acuucaaaUcAa--guCc-----------UUcCGG--G-----------------------------------------UaGca-----------------------------------CCGuAA---------------------------------------------gGCgGGGUcc---GAAGGC---ACCU-gGCaacagaa-----------GCCUUCa----------------ccu-----GCCG-UCUuc-------------------------------------------------------------------------GGG--CGGC-uauGGC-AAua---aacGGG----CGAUgcaau---aaaCCCaUUGaac-CCGGGggcgguaCCCGG-CGCCUCCacca
Rhiz.legu.         GGGGGCGaaacaggau--cgaCAA-G-GGUguaaa--gAUCG---uCUUu----UUGCUcGGCau----uGUaCCG------ccGUUau----------CGG-GCuaaa-a----------------uuguaguugcaaacgacaacuaugcg------------------------------------------------gaagcucGUCuCGCUG----cuuaa-------------------------------ucgCGGU--GuGAC-------------------------------------------------acuucaa-UcAA-agucc-----------UAGuGG-------------------------------------------UUcGca-----------------------------------CCuCUA---------------------------------------------gGCgGGGUcc---GAAGGC---ACCU-gGCaacagaa-----------GCCUUCa----------------ccu-----GCCG-GCUuucg-----------------------------------------------------------------------GGU--CGGC-uauGGC-GAua---aauGAG----CGGUguaau---aaACCUaUUGgac-CCGGGggcgguaCCCGG-CGCCUCCacca
Agro.tume.         GGGGGCGaaauaggau--cgaCAA-G-GGcguaaa--gAUCG---aACUu----UUGCUcGGCau----GAUaCCG------ccGUUau----------CGG-GUCaca-----------------aguguaguugcaaaugacaacaacgcu---------------------------------------------aaggaaUaCgcUcUCGCUG----ccuaa----------------------------------UGGC--GGuGcgG-G--------------------------------------------aauuccgcuCUAA--gucc-----------UUACGG-------------------------------------------UUAGcc-----------------------------------CCGUAA---------------------------------------------gGCgGGGUuc---GGAGGU---ACCU-gGCaacagaa-----------ACCUCCa----------------ccU-----GCCG-GGgcAa-------------------------------------------------------------------------CC--CGGC-uauGGC-GAua---aacGGU----CGGUguaau---aaaCCUaUUGgac-CCGGGggcgguaCCCGG-CGCCUCCacca
Caul.cres.         GGGGCCGau-cagcau--cgaCAGa--CGUguaaa--gGUGU---cUGCuu---UCUCUcGGCuu----GG-CCCa------ccGUU-u----------cGGG-CCuuu------------------aucuaaaugcgaacgauaacuucgcu---------------------------------------------gaagagUUCGCCguCGCUG----cguaa--------------------------------ugCGGU--GcaGGUGAA---------------------------------------------uucgccuCUuAA--gucc-----------UAGGGG-------------------------------------------UUcAGa-----------------------------------UCCCUA---------------------------------------------gGcgGGGCcc---GGAGGGA--GCCU-gcCaacagaa----------UCCCUCCa----------------ccu-----GUcC-GGCuucg-----------------------------------------------------------------------GCC--GaAC-uauGGG-GAua---aacGCGc---ACGUaau-----aaGCGgaCUGgac-CCGGGugcgauuCCCGG-CGGCUCCacca
Sili.pome.         GGGGCCGaaacaggau--cgaCGGa--CGUcuaaa--gGGGU---uaGCuu---UGUCUcGGCGg----GGuACCa------cCGUUau----------cGGU-CCgcaaa----------------gcauaauugccaacgacaaucgugcu------------------------------------------------ccgguUGcUcUGGCUG----cguaa---------------------------------gCAGU-cCGaAaCA------------------------------------------------ccgaaacUUAa--gccc-----------UUGCGC-------------------------------------------cUAGca-----------------------------------GCGUAA---------------------------------------------gGCgGGGUuc---GCAGGU---ACCU-gGCaacagaa-----------ACCUGCa----------------uuu-----GUUC-CCu---------------------------------------------------------------------------cGG--GAAC-uauGGA-CAua---aacGC-----GCUCguaau---aaGCGguUCGgac-CCGGGggcgguaCCCGG-CGGCUCCacca
Rhod.spha.         GGGGCCGaaacaggau--cgaCGAa--CGUcuaaa--GGgGu---ugGCuu---UGUCCcGGUGa----GGuACCa------cCGUUau----------cGGU-CCgaa----------------aaguacaguugccaacgacaaccgugcu------------------------------------------------ccgGUGGCUCuGGCUG----cguaa---------------------------------gCAGU--UcGAGUUAU-----------------------------------------------cgaaacUUAA--gccc-----------UUGCGC-------------------------------------------UUAGcc-----------------------------------GCGUAA---------------------------------------------gGCgGGGUuc---GCAGGU---ACCU-gGCaacagaa-----------ACCUGCa-----------------cuU----GUCC-GGGGcaa-----------------------------------------------------------------------CCC--GGAC-uauGGA-CAua---aacGCgc---uCgUCau-----aaGCGgaUCGgac-CCGGGggcgguaCCCGG-CGGCUCCacca
Rick.prow.         GGGGGCGaaauaggau--cgaCGU-G-CCUaauaaa-aAAGU----UGCuu---UUACUcGGuau----GAuUCCAccggu-gguUUuugccaua----UGGA-UCaaaa----------------caaagaaacgcaaacgauaaucguuau------------------------------------------guagguguuccagcuuU-AGcUgcagcuuaa----------------------------------GcCU---A--------------------------------------------------------------------------------------------------------------------------------------------------------------------------------------------------------------------------------CgCGGCug---GGGAUUU--GCCG-gGcaacagaa----------AAAUCCCa-----------------UAc----GCUA----aUA-----------------------------------------------------------------------------UAGC-uauAGU-AAua---aacGUA----AUUUagaau---agAGGUuGCGgac-UCGGGggcaguaCCCGA-CGCCUCCacca
Rick.typh.         GGGGGCGaaauaggau--cgaCGU-G-CCUaauaaa-aAAAU----UGCuu---UUACUcGGuau----GAuCCCAccgau-ggcUUuugccaua----UGGG-UCaaaa----------------caaagaaacgcaaacgauaauaagcgu---------------------------------------uauguagguguugccgcuuU-AGcugcagcuuaa----------------------------------ucCU---A--------------------------------------------------------------------------------------------------------------------------------------------------------------------------------------------------------------------------------CgCGGCuu---GGGAUUU--GCCG-gGcaacagaa----------AAAUCCCaug---------------------cGCUA----aua-----------------------------------------------------------------------------UAGC-uauAGU-AAua---aacGUA----AUUUagaau---agAGGUuGCGgac-ucgggggcaguacccga-CGCCUCCacca
Rick.cono.         GGGGGCGaaauaggau--cgaCGU-G-CCUaauaaa-aAGAU----UGCuu---UUACUCGGGau----GAuUCCGccgau-ggCUUGugccaua----UGGG-UCaaaa----------------caaagaaacgcaaacgauaauaaucguucu---------------------------------------guaggucauuuagcuuU-AGcAg---cuuaa----------------------------------UcCU---A--------------------------------------------------------------------------------------------------------------------------------------------------------------------------------------------------------------------------------CgCGGCugg-aGGGuGU---GCCG-gGcaacagaac---------aACcCUCa-----------------cuU----GCUA----Aua-----------------------------------------------------------------------------UAGC-uauAGU-AAua---aacGUA----AUUUagaau---agAGGUuGCGgac-UCGGGggcaguaCCCGA-CGCCUCCacca
Sphi.arom.         GGGGCCGaacuaggau--cgaCGU-G-UGUugaaa--aGCGU---UGUuu----UCACCCGGGcu----GAGU---------aaCCCGuucaa--------gGCUCaaa----------------acucacaagugccaacgacaacgaagcacuu---------------------------------------------------GCUCUcGcGG----cguaa----------------------------------UUcU--aGGGGC---------------------------------------------------------UAAc-gcC-------------CUGAa--G----------------------------------------UUAcua----------------------------------agUUAG----------------------------------------------aGCaCGGUucg--gaCCGA---ACCG-gGUaacagaa-----------UCGGaaa-----------------ccGg---GCCG-GUUCa-------------------------------------------------------------------------GGC--CGGC-uauGGU-GAua---aacuGCG---GCGUgcaau---agGCACaGCGgac-CCGGGggcgguaCCCGG-CGGCUCCacca
Ehrl.chaf.         GGGGAUGaaacaggau--cgaC-AUG-CAUaguaaa-gGGGA--uaGUUU----UUGCUCGGUga----GAaaAc--------aGCCGuuuaaa-------UgcUCaaaau----------------uuauaagugcaaacgauaauuucguuuuu------------------------gcuaaugauaauaauagcagugcuaacuUAGUAGcUG----cuuag-------------------------------uuuUA-U--UAUUA----------------------------------------------------------------------------------------------------------------------------------------------------------------------------------------------------------------------------GCgCGGuuuuuuuAAGGGuuuuCCG-gGCaacagaaa--------aaCCCUUu------------------uuu----GUGC--u-uuu--a--------------------------------------------------------------------------GCAC-uauGGC-AAua----aAGGCa---UCUUaaaau---agAUGUAUuGgac-CCGAGggcagugCUCGG-CAUCUCCacca
Wolb.spec.         GGGGACGaauaaggau--cgaC-AUG-CAUagu----AaAGau--GGUAGcu--UUGCUCGGUua----GACaC---------cACCGcuaa---------GaGUUcau-----------------aauuuaaaugcaaacgauaauuuugcugcu------------------------------------gaagacaaugucgacgcaaUaGCUg----cuuaa-------------------------------uuuaAGC---Ac--------------------------------------------------------------------------------UAUAaC----------------------------------------------------------------------uucauu--------GcUAUA---------------------------------------------aGCaCGGUuca---GGGAA--cGCCG-gGUaacagaag----------UUCCCca-----------------AAA----GUAC-au-UUU--gu-------------------------------------------------------------------------GUAC-uauAGC-AAua--aaaUUACC----UUgUaau----agGUGUAUuGgac-CCGAGggcaguaCUCGG-CGUCUCCacca
Chro.viol.         nnnnnnnnnnnnnnn---nnnCGGGG-GUUgcgaa--GCAGa----UGAGg--GCAUACCGGGau---uUCAGUc-------acCCCGuaaaac-------GCUGAauu-----------------uauauagucgcaaacgacgaaacuuac------------------------------------------------------gcUCUGGCaG----ccuaa------------------------------cggcCgGC--CAGA------------------------------------------CACUACA------------aCGGUUC-----------GCAGAU-GGGCCGg--------------GGGc----gucaaa--aCCC----------------------UGUAGUGucacu----cuacAUCUGCua--------------------------------------gUGCuGUU---CCGg---GUUACU--UGGuu-cAGU-GCGaaau-----aauaGGUAACuc--GCCaaaGUCca---GCCu---GUCc-GUCGGC--guGGCa-gaGGUuaaa-----------------------------------------------uccaaauGACa-cGAC-uaaGUA-UGUa--gaaCUCA-----CUGUag-----agGACUUUCGgac-gcGGGuucaacuCCCnn-nnnnnnnnnnn
Neis.gono.         GGGGGCGaccuugguuu-cgaCGGGG-GUUgcgaa--GCAGA----uGCGg--GCAUACCGGGGu---cUCAGaU--------UCCCGuaaaac------A-CUGAauu-----------------caaauagucgcaaacgacgaaacuuac------------------------------------------------------GcuUUAGCCg----cuuaa-----------------------------------GGC--UAG--C----------------------------------------CGUUGCa-------------GCAGUCgg---------UCAAUG-GGCUGUg--------------UGGC----gaaa----GCCA---------------------ccGCAACGucau-----cuuaCAUUGAcug-------------------------------------GUUUCCa---GCCGg---GUUACU--UGGC----aGGAAAU--aaga-cuuaaGGUAACug--GUUUCCAaaA---gGCCu---GUUG-GUCGGC--aUgaUG-GAAAUaaga--------------------------------------------uuuucaaauaGACa-CAAC-uaaGUA-UGUa--gaaCGC-----UUUGUag-----agGACUUUCGgac-GGGGGuucgauuCCCCC-CGCCUCCacca
Neis.meni.-1.      GGGGGCGaccuugguuu-cgaCGGGG-GUUgcgaa--GCAGA----uGCGg--GCAUACCGGGGu---cUCAGaU--------UCCCGuaaaac------A-CUGAauu-----------------caaauagucgcaaacgacgaaacuuac------------------------------------------------------GcuUUAGCCg----cuuaa-----------------------------------GGC--UAG--C----------------------------------------CGUUGCa-------------GCAGUCgg---------UCAAUG-GGCUGUg--------------UGGC----gaaa----GCCA---------------------ccGCAACGucauc-----uuaCAUUGAcug-------------------------------------GUUUCCU---GCCGg---GUUAUU--UGGC----AGGAAAU--gaga-uuuaaGGUAACug--GUUUCCAaaA---gGCCu---GUUG-GUCGGC--aUgaUG-GAAAUaaga--------------------------------------------uuuucaaauaGACa-CAAC-uaaGUA-UGUa--gaaCGC-----UUUGUag-----agGACUUUCGgac-GGGGGuucgauuCCCCC-CGCCUCCacca
Neis.meni.-2.      GGGGGCGaccuugguuu-cgaCGAGG-GUUgcgaa--GCAGA----uGCGg--GCAUACCGGGGu---cUCAGaU--------UCCCGuaaaac------A-CUGAauu-----------------caaauagucgcaaacgacgaaacuuac------------------------------------------------------GcuUUAGCCg----cuuaa-----------------------------------GGC--UAG--C----------------------------------------CGUUGCa-------------GCAGUCgg---------UCAAUG-GGCUGUg--------------UGGC----gaaa----GCCA---------------------ccGCAACGucauc-----uuaCAUUGAcug-------------------------------------GUUUCCU---aCCGg---GUUACU--UGGc----AGGAAAU--aaga-cuuaaGGUAACug--GUUUCCAaaA---gGCCu---GUUG-GUCGGC--aUgaUG-GAAAUaaga--------------------------------------------uuuucaaauaGACa-CAAC-uaaGUA-UGUa--gaaCGC-----UUUGUag-----agGACUUUCGgac-GGGGGuucgauuCCCCC-CGCCUCCacca
Uncu.u02-.         nnnnnnnnnnnnnnnnnnnnnnGUGG-AUUgcaaa--GCUAU----cGAGg--GCGUGUCGAGGa----UCAGuUa-------CCUCGuaaauc-----cAuCUGAaaa-----------------acaguaaacgcaaacgacgaacaguuu------------------------------------------------------gcUCUAGCaG----cuuaa------------------------------ucgcUcGC--UAGA------------------------------------------UCUCACa-------------CCAGCUug---------CCGAUG-GGCUGGg--------------CAGGg---ucaa---aCCUG---------------------caGUGGGAucauc-----gcaCAUCGGauc---------------------------------------GCUGC--gUGCAgg--GUUGCGA-UGCAa---GUGGCuaaaucaagcgacUCGCGGUu---GUCGUa--------GGGu---GUCc-CcUCCC-----gacaAUGAUcguuaaaa--------------------------------------------ccaaauAaGa-cGAC-uacACA-CGUa--gaaCUU-----GUAGUgg-----agGAUUUGCggac-guggnnnnnnnnnnnnn-nnnnnnnnnnn
Nitr.euro.         GGGGGCGauc-cgguuu-cgaCGUGG-GUUgcaaa--GCAGc----GCAGg--GCAUACCGAGGu----UCAGUa-------cCCUCGuaaaac------aGCUGAaaa-----------------aaaauagucgcaaacgacgaaaacuac------------------------------------------------------gcUUUAGCCG----cuuaa--------------------------------ucCGGC--UAAA------------------------------------------CUCCACa-------------CCGGUGgg---------CCUCaa-CGCCGGg--------------UCUG----gcaa----CAGA---------------------caGUGGAGucau------uaac-GAGGauc---------------------------------------GCGUUU--UGCAgg--GUUACUU-UGCA---GAGCGCuaaaaaa-----uAGGUAACuc--GCCUGUcaUC---aGCCu---GCCG-GUUGGC---GGauGcCAGGUcaaa------------------------------------------------uuaaauAACa-UGGC-uaaGUA-UGUa--gaaCUGU-----CUGUag-----agGACUUGCGgac-GCGGGuucaauuCCCGC-CGCCUCCacca
Meth.glyc.         nnnnnnnnnnnnnnnnnnnnnCGgGG-GUUgcaaa--GCAGc----GCAGg--GCAUACCGAGGc----CUAGUca-------CCUCGuaaauaa------ACUAGaaca----------------aguauagucgcaaacgacgaaacuuac------------------------------------------------------gcUCUAGCCG----cuuaa-------------------------------uccCGGC--UGGA------------------------------------------CGCUGCa-------------CCGAaggg---------CCUCUC-ggUCGGg--------------UGGG----guaa----CCCA---------------------caGCAGCGucau------uaaGAGAGGa---------------------------------------UcGUGCGA--UAUUgg--GUUACUU-AAUA---UCGUAUuAaaucc-----aAGGUAACuc--GCCUGcUGUUu---GCUu---GCUc-GUUGGU--gAGCAu-CAGGUuaaa------------------------------------------------ucaaacAACa-cAGC-uaaGUA-UGUa--gaaCUGU-----CUGUgg-----agGGCUUgCGgac-gggGGuucgauuCCnnn-nnnnnnnnnnn
Nitr.cryo.         nnnnnnnnnnnnnnnnnnnnnCGUGG-GUUgcaaa--GCAGc----GCAGg--GCAUACCGAGGa----CCAGAaUa------CCUCGuaaauac----A-UCUGGaaa-----------------aaaauagucgcaaacgacgaaaacuac------------------------------------------------------GcuUUAGCCG----cuuaa-------------------------------ua-CGGC--UAG--C----------------------------------------CUCUGCa-------------CCGAUGgg---------CCUUaa-CGUCGGg--------------UCUG----gcaa----CAGA---------------------caGCAGAGucau------uagc-AAGGa---------------------------------------UcGCGUUC--UGUAgg--GUCACUU-UACA---GAACGUuAaacaa-----uAGGUGACuc--GCCUGcCAuCa---GCCc---GCCA-GCUGGC--gGuUGu-CAGGUuaaa------------------------------------------------uuaaagAGCa-UGGC-uaaGUA-UGUa--gaaCUGU-----CUGUag-----agGACUUGCGgac-gcGGGuucaacuCCCnn-nnnnnnnnnnn

Uncu.u04-.pa.      nnnnnnnnnnnnnnnnnnnnnngugg-guugcaaa--gcagu----gcagg--gcauacCGAGGa----CUAGUca-------CCUCGuaaaucc------ACUGGaacaa---------------guuauagucgcaaacgacgaaacuuac------------------------------------------------------gcUCUAGCCG----cuuaa-------------------------------uccCGGC--UGGA------------------------------------------CGCUGCa-------------CCGGaggg---------CCUCUC-ggCCGGg--------------CGGA----gcaa----UCCG---------------------caGCAGCGucau------auaGAGAGGa---------------------------------------ucgu------------------------------------------------------------------------------------------------------------------------------------------------------------------------------------------------------------------------------------------------------------
Alca.faec.         nnnnnnnnnnnnnnnnnnnnnCGgGG-GUCaagaa--GCAGc----ACAGg--GCGUGUCGAGCa----CCAGUac-------GCUCGuaaaucc------ACUGGaaa-----------------acuauaaacgccaacgacgagcguuuc------------------------------------------------------GcUCUAGCCg----cuuaa-----------------------------------GGC--UGGG-C----------------------------------------CACUGCa-------------CUAAUUug---------UCUUUG-GGUUAGg--------------UAGG----gcaa----CCUA---------------------caGCAGUGuuau------uuaCAAAGAa---------------------------------------UcGAAUCG-GuCUgc---gCCACg--aAGuC--CGGUUCuAaaa-----cuua-GUGGauc--GCCaaGGAaag---GCCu---GUCA-AUUGGC-auagUCC-aaGGUuaaa--------------------------------------------acuuaaaauuAAU--UGAC-uacACA-UGUa--gaaCUGU-----CUGUgg-----acGGCUUgCGgac-ggGGGuucgauuCCCnn-nnnnnnnnnnn
Bord.para.         GGGGCCGauc-cggauu-cgaCGUGG-GUCaugaa--aCAGC----uCAGg--GCAUGCCGAGCa----CCAGUaa-------GCUCGuuaaucc------ACUGGaac-----------------acuacaaacgccaacgacgagcguuuc------------------------------------------------------GcuCUCGCCG----cuuaa---------------------------------gCGGU--GAG--C----------------------------------------cGCUGCa-------------CUGAUCug---------UCCUUG-GGUCAG-------gc-gggg-GAAG----gcaa----CUUCccagggggcaaccccgaa---ccGCAGCagcgaca---uucaCAAGGAa---------------------------------------UcGGCCAc-CGCUGgg--gUCACa--CGGCG--uUGGUUuAaa-------uuacGUGAauc--GCCCUGGuCcg---GCCc---GUCG-AUCGGC-uaaGuCC-AGGGUuaaa----------------------------------------------uccaaauaGAU--CGAC-uaaGCA-UGUa--gaaCUG-----GUUGcgg-----agGGCUUGCGgac-GGGGGuucaauuCCCCC-CGGCUCCacca
Bord.pert.         GGGGCCGauc-cggauu-cgaCGUGG-GUCaugaa--aCAGC----uCAGg--GCAUGCCGAGC----aCCAGUaa-------GCUCGuuaauc------cACUGGaac-----------------acuacaaacgccaacgacgagcgucuc------------------------------------------------------GcuCUCGCCG----cuuaa---------------------------------gCGGU--GAG--C----------------------------------------cGCUGCa-------------CUGAUCug---------UCCUUG-GGUCAG-------gc-gggg-GAAG----gcaa----CUUCacagggggcaaccccgaa---ccGCAGCagcgaca---uucaCAAGGAa---------------------------------------UcGGCCAc-CGCUGgg--gUCACa--CGGCG--uUGGUUuAaa-------uuacGUGAauc--GCCCUGGuCcg---GCCc---GUCG-AUCGGC-uaaGuCC-AGGGUuaaa----------------------------------------------uccaaauaGAU--CGAC-uaaGCA-UGUa--gaaCUG-----GUUGcgg-----agGGCUUGCGgac-GGGGGuucaauuCCCCC-CGGCUCCacca
Bord.bron.am.      GGGGCCGauc-cggauu-cgaCGUGG-GUCaugaa--aCAGC----uCAGg--GCAUGCCGAGCa----CCAGUaa-------GCUCGuuaauc------cACUGGaac-----------------acuacaaacgccaacgacgagcguyuc------------------------------------------------------GcuCUCGCCG----cuuaa---------------------------------gCGGU--GAG--C----------------------------------------cGCUGCa-------------CUGAUCug---------UCCUUG-GGUCAG-------gc-gggg-GAAG----gcac----UUUCncagngggcacnnccgaa---ccGCAGCagcgaca---uucaCAAGGAa---------------------------------------UcGGCCAc-CGCUGng--gUCACa--CGGCG--uUGGUUuAaa-------uuacGUGAauc--GCCCUGGuCcg---GCCc---GUCG-AUCGGC-uaaGuCC-AGGGUuaaa----------------------------------------------uccaaauaGAU--CGAC-uaaGCA-UGUa--gaaCUG-----GUUGcgg-----agGGCUUGCGgac-GGGGGuucaauuCCCCC-CGGCUCCacca
Coma.test.         GGGGCCGauucuggauu-cgaCGUGG-GUUcggg---aCCGG---uGCGGu--GCAUGUCGAGCu----uGAGUGac------GCUCGuaaaucuc----CAUUCaaa------------------aaacuaacugcaaacgacgaacguuuc------------------------------------------------------gcaCUCGCCG----cuuaa--------------------------------ucCGGU--GAG-------------------------------------------CCUUGCA-------------aCAGCacg---------CUAGUG-gGCUGg-------gcaaggg-GGUA----gcaa----UACC-----------ucccgg----cUGCAAGGgaa------uuuuCAUUAG----------------------------------------CUgGCUGGAUACCGg---GCUUCU--UGGUA-UUUGGCgAGauu------uuaGGAAGCug--GCUACCCAAGc-a-gCGu---GUGC-CUGCGg-ggUUUGG-GUGGUgagau-----------------------------------------------uuaaaaCAGa-GCAC-uaaACA-UGUa--gauCUGU----CCGGcg------aaGGCUUACGgac-GCGGGuucaauuCCCGC-CGGCUCCacca
Hydr.pall.         nnnnnnnnnnnnnnnnnnnnnCGUGG-GUUcgga---cgCGc---AGCAGg--GCAUGUCGAGGu-----UCUGuC------aCCUCGuaaauca----G-CAGAaaa------------------aaaccaacugcaaacgacgaacguuuc------------------------------------------------------gcaCUCGCCG----cuuaa------------------------------acacCGGU--GAG-------------------------------------------CCUUGCA-------------aCAGCagg---------CCGAUG-gGCUGg-------gcaaggg-GGUC----gcaa----GACC-----------ucccgg----cUGCAAGGuaa------uuuaCAUCGG----------------------------------------CUgGUUCUGCGUCGg---GCAcCU--UGGCG-CAGGAUgAGauu------caaGGaUGCug--GCUuCCCGuuu-a-GCGu---GCCa-CUGCGC-gacuCGG-GcGGUgagac-----------------------------------------------ccaaauCAGa-cGGC-uacACA-UGUa--gaaCUGCU----CGaaa------aaGGCUUGCGgac-ggGGGuucaacuCCCnn-nnnnnnnnnnn
Vari.para.         nnnnnnnnnnnnnnnnnnnnnCGUGG-GUUcgga---GUCGC---aGCGGg--GCAUGUCGAGCu-----GAAUgC-------GCUCGuaaaaca----G-AUUCaaac-----------------aaacuaacugcaaacgacgaacguuuc------------------------------------------------------gcaCUCGCUG----cuuaa------------------------------uugcCAGU--GAG-------------------------------------------CCUUGCA-------------aCAGUUgg---------CCgAUG-GGCUGg-------gcaaggg-GGUCuggagcaauccuGACC-----------ucccgg----cUGCAAGGaua------acuaCAUgGG----------------------------------------CUgGCUCCGAUCCGg---GUACCU--UGGGU-CGGGGCgAGaaa------auaGGGUACug--GCgUCCGGuuu-a-GCGu---GUGA-CUGCGC-gacuCCG-GAaGUgagac-----------------------------------------------ucaaaaCAGa-UCAC-uaaACA-UGUa--gaaCUGC----GCGAUg------aaGGCUUGCGgac-ggGGGuucaacuCCCnn-nnnnnnnnnnn
Burk.pseu.         GGGGGCGacc-ugguuu-cgaCAGGG-GUugugaa--GCGGC-----UAGg--GCAUGUCGAGGa----CCCGUca-------CCUCGuuaauca------AUGGGaaa-----------------aacguaacugcaaacgacgauacguuc------------------------------------------------------gcacUGGCaG----ccuaa-------------------------------gggCcGC--CGu-------------------------------------------CCUCUGc-------------CUAGUUca---------CUGACG-GGCUAGu-gu--cgcaagaccGGUA----gcaa----UACCg---------------------aCAGAGGuca------uauaCGUCAGuua------------------------------------aG-CCUGU---CCGgc---gUCACg--aCGG----ACAGGuCgaaa----aucaaGUGAauc--GCCGUaACGga---GCGu---GUUC-GUCCGC--gaCGUc-ACGGUuaaa------------------------------------------------ucaaauGACa-GAAC-uaaACA-UGUa--gaaCUG-----GUCGUgg-----accGCUUCUGgac-GCGGGuucgauuCCCGC-CGCCUCCacca
Burk.mall.         GGGGGCGacc-ugguuu-cgaCAGGG-GUugugaa--GCGGC-----UAGg--GCAUGUCGAGGa----CCCGUca-------CCUCGuuaauca------AUGGGaaa-----------------aacguaacugcaaacgacgauacguuc------------------------------------------------------gcacUGGCaG----ccuaa-------------------------------gggCcGC--CGu-------------------------------------------CCUCUGc-------------CUAGUUca---------CUGACG-GGCUAGu-gu--cgcaagaccGGUA----gcaa----UACCg---------------------aCAGAGGuca------uauaCGUCAGuua------------------------------------aG-CCUGU---CCGgc---gUCACg--aCGG----ACAGGuCgaaa----aucaaGUGAauc--GCCGUaACGga---GCGu---GUUC-GUCCGC--gaCGUc-ACGGUuaaa------------------------------------------------ucaaauGACa-GAAC-uaaACA-UGUa--gaaCUG-----GUCGUgg-----accGCUUCUGgac-GCGGGuucgauuCCCGC-CGCCUCCacca
Burk.cepa.         GGGGGCGacc-ugguuu-cgaCAGGG-GUugugaa--GCGGC-----UAGg--GCAUGUCGAGGa----CCCGUca-------CCUCGuuaauca------AUGGGaaa-----------------aucguaacugcaaacgacgauacguuc------------------------------------------------------gcacUGGCaG----ccuaa-------------------------------gggCcGC--CGu-------------------------------------------CCUCUGc-------------CUAGUUca---------CUGACG-GGCUAGu-gu--cgcaagaccGGUA----gcaa----UACCg---------------------aCAGAGGuca------uuuaCGUCAGuua------------------------------------aG-CCCUG---uCGgc---gUCGCg--aCGc----CAGGGuCgaaa----aucuaGCGAauc--GCCGUAGUGca---GCGu---GUUC-GUCCGC--guCGCU-GCGGUuaaa------------------------------------------------ucaaaaGACu-GGAC-uaaACA-UGUa--gaaCUA-----GUCGUgg-----agcGCUUCUGgac-GCGGGuucgauuCCCGC-CGCCUCCacca
Burk.fung.         GGGGGCGacc-ugguuu-cgaCAGGG-GUugcgaa--GCGGC-----UAGg--GCAUGUCGAGGa----CCCGUca-------CCUCGuuaauca------AUGGGaaa-----------------aacguaacugcaaacgacgauacguuc------------------------------------------------------gcacUGGCaG----ccuaa-------------------------------gggCcGC--CGu-------------------------------------------CCUCUGc-------------CUAGUUua---------CUGACG-GGCUAGa-gu--cgcaagaccGGUA----gcaa----UACCg---------------------aCAGAGGuca------uauaCGUCAGuua------------------------------------aG-CCUuG---GUGgu---gUCACg--aCAU----CcGGGuCgaaa----aucuaGUGAauc--GCCGUAGUGca---GCGu---GUUC-GUCCGC--guCGCU-ACGGUuaaa------------------------------------------------ucaaauGACa-GAAC-uaaACA-UGUa--gaaCUG-----GUCGUag-----aguGCUUCUGgac-GCGGGuucgauuCCCGC-CGCCUCCacca
Alca.eutr.         uGGGCCGacc-ugguuu-cgaCGUGG--UUacaaa--GCAGu-----GAG---GCAUACCGAGGa----cCCGUca-------CCUCGuuaauca------AUGGaau------------------gcaauaacugcuaacgacgaacguuac---------------------------------------------------------GCacUCG----cuuaa-------------------------------uugCGGc--cGU-------------------------------------------CCUCGCa-------------CUGGCUcg---------CUGACG-GGCUAG---------------GGUC----gcaa----GACC---------------------acGCGAGGuau-------uuaCGUCAGaua------------------------------------aG-CUCCG---Gaagg---GUCACg--aagC----CGGGGaCgaaa----accuaGUGACuc--GCCGuCGUAg---agCGu---GUUC-GUCCGa----UGCG-cCGGUuaaa------------------------------------------------ucaaauGACa-GAAC-uaaGUA-UGUa--gaaCUCu-----CUGUgg-----agGGcUUACGgac-GCGGGuucgauuCCCGC-CGGCUCcacca
Rals.eutr.         nnnnnnnnnnnnnnnnnnnnnCGUGG-GUUacaaa--GCAGu----GGAGg--GCAUACCGAGGa----CCCGUca-------CCUCGuuaauca------AUGGGaau-----------------gcaauaacugcuaacgacgaacguuac------------------------------------------------------gcacUGGCCG----cuuaa-------------------------------uugCGGC--CGu-------------------------------------------CCUCGCa-------------CUGGCUcg---------CUGACG-GGCUAG---------------GGUC----gcaa----GACC---------------------acGCGAGGuca------uuuaCGUCAGaua------------------------------------aG-CUCCG---Gaagg---GUCACg--aagC----CGGGGaCgaaa----accuaGUGACuc--GCCGuCGUAga---GCGu---GUUC-GUCCGC--gaUGCG-cCGGUuaaa------------------------------------------------ucaaauGACa-GAAC-uaaGUA-UGUa--gaaCUCU-----CUGUgg-----agGGCUUACGgac-GCGGGuucgauuCCCGC-cggcuccacca
Rals.meta.         GGGGCCGacc-ugguuu-cgaCGUGG-GUUacaaa--GCAGu----GGAGg--GCAUACCGAGGa----CCCGUca-------CCUCGuuaauca------AUGGGaau-----------------gcaauaacugcuaacgacgaacguuac------------------------------------------------------gcacUGGCaG----ccuaa-------------------------------gggCcGC--CGu-------------------------------------------CCUCGCa-------------CUGGCUcg---------CUGACG-GGCUAG--------------gGUUC----gcaa----GAACc---------------------aGCGAGGuca------uuuaCGUCAGaua------------------------------------aG-CUUCG---GGagu---GUCACg--ggCC----CGAGGaCgaaa----accuaGUGACuc--GCCGuCGUAga---GCGu---GUUC-GUCCGC--gaUGCG-cCGGUuaaa------------------------------------------------ucaaauGACa-GAAC-uaaGUA-UGUa--gaaCUCU-----CUGUgg-----agGGCUUGCGgac-GCGGGuucgauuCCCGC-CGGCUCCacca
Rals.sola.         GGGGCCGacc-ugguuu-cgaCGUGG-GUUgcgaa--GCAGu----AGCGg--GCAUACCGAGGa----CCCGUca-------CCUCGuuaauca------AUGGGaac-----------------gcaauaacugcuaacgacaaccguuac------------------------------------------------------cagcUGGCcg----cuuaa------------------------------uugcgcGC--CGu-------------------------------------------CCUCGCa-------------CUAGCUug---------CCGAUA-GGCUAG---------------GGCC----gcaa----GGCC---------------------acGCGAGGuca------uucaUAUCGGauc-------------------------------------GCUUCGG---CCGgu---GUCACg--aCGG----CCGGGGUuaac----uccaaGUGACuc--GUCGuCGCGca---GCGu---GUUC-GUCCGC--guCGCG-cCGGUuaaa------------------------------------------------ucaaauGACu-GAAC-uaaGUA-UGUa--gaaCGCU-----CUGUag-----agGACUUGCGgac-GCGGGuucgauuCCCGC-CGGCUCCacca
Rals.pick.         nnnnnnnnnnnnnnnnnnnnnCGgGG-GUUgcgaa--GCAGc----GGAGg--GCAUACCGAGGa----CCCGUca-------CCUCGuuaauca------AUGGGaau-----------------gcaauaacugcuaacgacgaacguuac------------------------------------------------------gcacUGGCaG----ccuaa-------------------------------gggCcGC--CGu-------------------------------------------CCUCGCa-------------CUGGCUcg---------CUGACG-GGCUAG---------------GGUC----gcaa----GACC----------------------aGCGAGGuca------uuuaCGUCAGaua------------------------------------aG-CUUUA---GGUga---gUCACg--gGCC----UAGAGaCgaaa----acuuaGUGAauc--GCCGUCGUAga---GCGu---GUUC-GUCCGC--gaUGCG-GCGGUuaaa------------------------------------------------ucaaauGACa-GAAC-uaaGUA-UGUa--gaaCUCU-----CUGUgg-----agGGCUUgCGgac-gcGGGuucgauuCCCnn-nnnnnnnnnnn
Acid.ferr.-1.      GGGGGCGac--cggcuu-cgaCGCAG-guCgcgaa--GCCuU---CGGu----GCAUGCAGAGCu----GCGGUUc-------GCUCUuaaaacu-----GGUCGCaga-----------------uucauaauugccaacgacagcaauuac------------------------------------------------------gcuCUCGCUG----cuuaa---------------------------------gCAGU--GAG-------------------------------------------CCCUCUG-----------cCCGGAUUug---------UCUGUG-GAUCCGg--------------aGCC----gaaa----GGC--Gc------------------gCGGAGGGucau-----gaaaCACGGAauc-------------------------------------GUGCGCAU--UCUA----gUCUG---UGGG---AUGCGCGCuaaa---uuaaagCAGAauc--GCCaUGUCGUc--aUCCu---GUcU-GUCGGA-ggGCGGU-GcGGUuaaa-----------------------------------------------gccaaaaGAC--AaAC-uacGCA-UGUa----gauCCG---GuGGUcu-----agGuaCUGCGgac-GCGGGuucaauuCCCGC-CGCCUCCacca
Acid.ferr.-2.      GGGGGCGac--cggcuu-cgaCGCAG-guCgcgaa--GCCuU---CGGu----GCAUGCAGAGCu----GCGGUUc-------GCUCUuaaaacu-----GGUCGCaga-----------------uucauaauugccaacgacagcaauuac------------------------------------------------------gccCUCGCUG----cuuaa---------------------------------gCAGU--GAG-------------------------------------------CCCUCUG-----------cCCGGAUUug---------UCUGUG-GAUCCGg--------------aGCC----gaaa----GGC--Gc------------------gCGGAGGGucau-----gaaaCACGGAauc-------------------------------------GUGCGCAU--UCUA----gUCUG---UGGG---AUGCGUGCuaaa---uuaaagCAGAauc--GCCaUGUCGUc--aUCCu---GUcU-GUCGGA-ggGCGGU-GcGGUuaaa-----------------------------------------------gccaaaaGAC--AaAC-uacGCA-UGUa----gauCCG---GuGGUcu-----agGuaCUGCGgac-GCGGGuucaauuCCCGC-CGCCUCCacca
Fran.tula.         GGGGGCGaauaugguuu-cgaCAUGA-AUgucaaa--AUCUaa---GGu----GCAUGCCGAGGa---aGUACcGU-----aaCCUCGuuaaua-----ACaGUACaaa------------ugccaauaauaacuggcaacaaaaaagcaaaccgcguagcggcuaacgacagcaac------------------uuugcugcuguugcUaAAGCUG----ccuag--------------------------------ucUAGC--UUaAua-----------------------------------aucuaGAUGcGc--------ACGGAUAUGAua----------GUCUUu-cUUAUGac-------------acUA----ucua----UAca-UCCGUu----------------CaUAUUccg--------cauAAGACgguc------------------------------------U-UuGCU---UUUU----GUCUG---GGAGuuaaGGCuGuAu------uuaa--CAGACuc--GCUaaCUAUU---aCCCu---GGCUaAUUGGG--gAAUAGucaAGCuaaa----------------------------------------------cucaaauaGAUu-AGCC-uaaGCA-UGUa----gauCCa---aAGAUcu-----agaGUUUGUGgac-GCGGGuucaaauCCCGC-CGCCUCCacca
Dich.nodo.         nnnnnnnnnnnnnnnnn-nnnnnnnn-nnnnnnnn--nCUcga---GGU----GCAUGUCGAGAa----UGAGAG------aaUCUCGuuaaaua-----CUUUCAaaa-----------------cuuauaguugcaaacgacgacaacuac------------------------------------------------------gcuuuAGCGG----cuuaa-------------------------------uucCCGC--Uuu-------------------------------------------CGCUUAC------------cUAGAUUug---------UCUGUG-GGUUUAc-------------------------------------------------------cGUAAGCGacau------uaaCACAGAauc--------------------------------------GCUGGU--UAACG---cgUCCGC--UGUUA---AUCGGUuaaa----uuaa-GCGGAauc--GCUUGcAAAa---uGCCu---GAGC-GUUGGC--ugUUUa-UGAGUuaaa-----------------------------------------------ccuaauuAACu-GCUC-uaaACA-UGUa----guACCa---aaAGuua-----aggauucgcggac-GGGGGuucaaauCCCCC-cgccuccacca
Xyle.fast.-1.      GGGGGUGca-cuggcuu-cgaCGGGG-GUcacaaa--AUCaCu--UGGU----GCAUGCCGAGGg---gGCcGCUu------uCCUCGuaaa-----uccAGCaGCaaa-----------------cuuauaguugccaacgaagacaacuuc------------------------------------------------------gcugUAGCCg----cuuaa-----------------------------------GGC-uUAa-------------------------------------------GCcUcG--------aaaC--UGCuuGug---------UCCGUG-CucGCA--------------------------------------Gc-----------------gUaGaGU-------caucauCACGGAa--------------------------------------ccGCUGAGacGUGGc---cGUCUGC---CCAUc--CUCGGUuaaaucua-----GCAGACug--GUUuCCGGGU---gCGCu-uugCCU-GUCGUG-cuGCCCG-GcGACgag------------------------------------------------accuaacGGCg-AGGa-uaaGCA-UGUa----ggGCCG---GgGAUgg-----aguACUUUCGgac-GGCGGuucgauuCCGCC-CACCUCCacca
Xyle.fast.-2.      GGGGGUGca-cuggcuu-cgaCGGGG-GUcacaaa--AUCaCu--UGGU----GCAUGCCGAGGg---gGCcGCUu------uCCUCGuaaa-----uccAGCaGCaca-----------------cuuauaguugccaacgaagacaacuuc------------------------------------------------------gcucUAGCCg----cuuaa-----------------------------------GGU--UAa-------------------------------------------GCcUcG--------aaaC--UGCuuGug---------UCCGUG-CucGCA--------------------------------------Gc-----------------gUaGaGU-------caucauCACGGAa--------------------------------------ccGCUGAGacGUGGc---uGUCUGC---CCACa--CUUGGUuaaaucua-----GCAGACug--GUUuCCGGGU---gCGCu-uugCCU-GUCGUG-cuGCCCG-GcGACgag------------------------------------------------accuaacGGCc-AGGa-uaaGCA-UGUa----ggGCCG---GgGAUgg-----aguACUUUCGgac-GGCGGuucgauuCCGCC-CACCUCCacca
Xyle.fast.-3.      GGGGGUGca-cuggcuu-cgaCGGGG-GUcacaaa--AUCaCu--UGGU----GCAUGCCGAGGg---gGCcGCUu------uCCUCGuaaa-----uccAGCaGCaca-----------------cuuauaguugccaacgaagacaacuuc------------------------------------------------------gcuaUAGCCg----cuuaa-----------------------------------GGC-uUAa-------------------------------------------GCcUcG--------aaaC--UGCuuGug---------UCCGUG-CucGCA--------------------------------------Gc-----------------gUaGaGU-------caucauCACGGAa--------------------------------------ccGCUGAGacGUGGc---cGUCUGC---CCACc--CUUGGUuaaaucua-----GCAGACug--GUUuCUGGGU---gCGCu-uugCCU-GUCGUG-cuGCCCG-GcGACgag------------------------------------------------accuaacGGCc-AGGa-uaaGCA-UGUa----ggGCCG---GgGAUgg-----aguACUUUCGgac-GGCGGuucgauuCCGCC-CACCUCCacca
Xant.camp.         GGGGGUGca-cugguuu-cgaCGGGG-GUcgcgaa--GUCgCu--UGGC----GCAUGCCGAGGg---gGCaGCUu------uCCUCGuuaa-----uccAGCaGCaaa-----------------cuuuuaguugccaacgacgacaacuac------------------------------------------gguucggacuucGCUAucGCCG----cuuaa---------------------------------uUGGC--uuUAGUu--------------guaguucuacagccgccuaaggcgaACCCccg-------aAaC--UACuuGug---------cCCGUG-CucGUA--------------------------------------GaU----------------guaGGGU-------cauuauCACGGaa--------------------------------------ccGCCGGUa-gUGgc---uGCCUGU----CAgcu-AUCGGUuagaucaa-----GCAGGCug--GUUUUCAGGU---gCGCu-uugCCC-gCCGUG-cuGCCUG-GGAACgag------------------------------------------------aucuaacGGa--GGGa-uaaGCA-UGUa----guGCUG---GgGAUgg-----aguGCUUCCGgac-GGCGGuucgauuCCGCC-CACCUCCacca
Xant.axon.         GGGGGUGca-cugguuu-cgaCGGGG-GUcgcgaa--GUCgCu--UGGC----GCAUGCCGAGGg---gGCaGCUu------uCCUCGuuaa-----uccAGCaGCaaa-----------------cuuuuaguugccaacgaugacaacuac------------------------------------------gguucggacuucGCUAucGCCG----cuuaa---------------------------------uUGGC--uuUAGUu--------------guaguucuacagccgccuaaggcgaACCCccg-------aACC--UACuuGug---------cCCGUG-CucGUA--------------------------------------GGU----------------guaGGGU-------cauuauCACGGaa--------------------------------------ccAUCGGUa-gUGgc---uGCCUGU----CAgcu-ACCGGUcagauaaa-----GCAGGCug--GUUUCCAGGU---gCGCu-uuGCCa-aCCGUG-cuGCCUG-GGAACgag------------------------------------------------aucuaacGGa--gGGC-uaaGCA-UGUa----guGCUG---GgGAUgg-----aguGCUUCCGgac-GGCGGuucgauuCCGCC-CACCUCCacca
Legi.pneu.         nnnnnnnnnnnnnnnnn-nnnnGUGG-GUUgcaaa--aCCgga---AGU----GCAUGCCGAGA----agGAGaUc-------UCUCGuaaau-----aaGaCUCaauuaaa----------------uauaaaugcaaacgaugaaaacuuu------------------------------------------gcugguggggaagCUAucGcUG----ccuaa------------------------------uaagCAcU---uUAGuu----------aaaccaucacuguguacuggccaauaaacCcagUAU------cccGUUCgACCGa----gccc---GCUUAU--CGGUa----------------------------------ucGAAU---------caacggucAUAagaG-------------AUAAGCua--------------------------------------GCGUCc---UAAU---cuaUCCCgG--GUUAu----GGCGCgaaa-----cuCaGGGAauc--GCUGUGUaUC---aUCCu---GCCc-GUCGGA---GGaGCcACAGUuaaa-----------------------------------------------uucaaaaGACa-aGGC-uauGCA-UGUa----gaGCUa---aaGGcag-----agGACUUGCggac-gcggnnnnnnnnnnnnn-nnnnnnnnnnn
Coxi.burn.         GGGGGCGac-caggcuu-cgaCGUGG-AUCcugaa--aCCcga---GAC----GCAUGUCGAGCc---gGCAACgaa-----gGCUCGuaaau--ccaucaGUUGCaaaca-----------------gauaguugcaaacgauaguaauuau---------------------------------------------cuccaagaagCUUAcGCA-------uaa-----------------------agcuucuuccguUGC---UGGGau--------------------------------------ucGCUUAU------agaUcccuaccaac----------GGCCAU----------------------------------------------Agcau-------------AUAAGCuc--gcccucaagcAUGGCU--------------------------------------caUGACUU---------------------------gaAGGUUAaa-------cagaagagcuc--GCAGUCAAUUuc--CCCu---GCAc-GUCGGG--cGAUUG-ACUGUuaaa-------------------------------------------------ucauaGACg-aUGC-uaaGCA-UGUa----guGUCa---acGGcgg-----auGAUUUACGgac-GCGGGuucaauuCCCGC-CGCCUCCacca
Meth.caps.         GGGGGCGac-auggcuu-cgaCGUGG-AUCgcgaa--aCCuga---GGU----GCAUGCCGAGGu----GCAGAUGa------CCUCGuaaau-----cCAUCUGCaaacca----------------aauaaacgccaacgacgacguuuac------------------------------------------------------gCAcuGGCCG----cuuaa----------------------------gccggcCGGC--CucUGau-----------------------------------cagcAGCucGC--------UUacaa--GCg--------gcuGguuggggGUaac-------------------------------------ccAAuu--------------GUaaGCU------------------C----------------------------------------GCGCUg---aaGUC---ugUCCGg--GGCcg----aAGCGCuaaaac---ccaaCGGAauc--GCCGucCGUcuuc-CCU----gCCA-CUCGGG--uaGCGu-cCGGCuaaa------------------------------------------------ucaauaGAG--UGGa-uacGCA-UGUa----gaACCg---auGGcag-----agGAUUUGCGgac-GCGGGuucaauuCCCGC-CGCCUCCacca
Uncu.u01a.         nnnnnnnnnnnnnnnnnnnnnnGCUG-GuAacgga--aUCCga---AGu----GCAUGCCGAGGu----GUCAUGu-------CCUCGuaaaa-----ccUGUGACaaccaa----------------gcuaguugcgaacgacuccaacuac------------------------------------------------------GCcCUCGCCG----ccuaa------------------------------auccCGGU--GAGcGU----------------------------------------CcAUccuu-------UCaGUGCCgAU--------acuGGUUggaAUgGGC------------------------------------GUcGAcu--------------cuuAUcG---------------GAUC----------------------------------------GUGGug---aaGCC---cgCCCUU--GGCcg----auCUGCuaaaac--ucuaAGGGGauaa-GCGUUGGagau---CCGu---GCCU-GUCCGG---gagCC-AACGUcgaaaa-----------------------------------------------ccaaaGAUc-AGGC-uaaGCA-UGUa----gauCUg---aGGGcuac----gaU-CUGGCggac-gcggnnnnnnnnnnnnn-nnnnnnnnnnn
Uncu.u01b.         nnnnnnnnnnnnnnnnnnnnnnGCUG-GuAacgga--aUCCga---AGu----GCAUGCCGAGGu----GUCAUGu-------CCUCGuaaa-----uccCGUGGCaaccaa----------------gcuaguugcgaacgacuccaacuac------------------------------------------------------GCcCUCGCCG----ccuaa------------------------------auccCGGU--GAGcGU----------------------------------------CcAUccuu-------UCGGUGCCgAU--------accGGuUggaAUgGGC------------------------------------GUCGAcu--------------cuuAUcG---------------GcUC----------------------------------------GCGGug---aaGCC---cgCCCUU--GGCcg----auCUGCuaaaac--ccuaAGGGGauaa-GCGUUGGagau---CCGu---GCCU-GUCCGG---gaaCC-AACGUcgaaaa-----------------------------------------------ccaaaGAUc-AGGC-uaaGCA-UGUa----gauCUg---aGGGcuac----gaU-CUGGCggac-gcggnnnnnnnnnnnnn-nnnnnnnnnnn
Acin.ADP1.         GGGGAUGuuauuggcuu-cgaCGCUG-GUGaugaa-gCUCaua---GAU----GCAUGCCGAGAg---cGCAuuUU------cUCUCGuaaa----uaaAAuuUGCauuuuaaua-----------------gucgcaaacgacgaaacuu------------------------------------------------------ACGcuCUaGCUg----ccuaa----------------------------------gGGC---AGuuUGUc-------------------------------------CGCUuCc--------UaGaaUaCUUG----------ugGUu---UAGGaA------------------------------------ccCgAc---------------cGuAGCG---------------cACgcac-------------------------------------acAAG-UCCGuaUa----gag-UCaA-GccUcGGGgCUUuauaccaaa-cuUaGA-ggauc--GCAUCUuGU----aCCCu---GuUc-GUCGGG--ucACuG-GGUGUuaaa------------------------------------------------acaauaGACgauAuC-uaaGCA-UGUa----guAUUc---ucGAGugu----agUGCUGGCGgac-GCGGGuucaacuCCCGC-CAUCUCCacca
Micr.degr.         GGGGGCGau-uaggauu-cgaCGcCG-GUAacaaa-acCCGa----AGU----GCAUGUCGUGAg---gGUAGCca------aUCACGuaaau---ccaaaGCUGCaaa-----------------uuaucaguugccaaugaugacaacuac------------------------------------------------ggugcuCAAcuAGCUG----cguaa---------------------------------gUAGC--UuuUUG-------------uuacaccaucuagcgguacgccgcuagCGGUUu-------uuAACa---aGG-----------ugCCAGU-aCC---------------------------------------gcGUU---------------aacGACUGucac----uuagaACUGGauc---------------------------------------GCaGug-aaGCCU----gCCUG---GGGUgg--auCaGCuaaac---uuuua-CAGGauc--GCGCUUUuCG---uCCC----GcCC-GCUGGG--cUGuAA-AGCGUuaaa------------------------------------------------ucaauaAGC--GGuUcuaaGCA-UGUa----gaGCUg---aCGGcgg-----agUACUGaCGgac-GGGGGuucaaauCCCCC-CGCCUCCacca
Pseu.aeru.         GGGGCCGau-uaggauu-cgaCGCCG-GUaacaaa-acUUGa----GGg----GCAUGCCGAGcu---gGUAGCAG------aaCUCGuaaau---ucgCUGCUGCaaa-----------------cuuauaguugccaacgacgacaacuac------------------------------------------------------gcuCUAGCUG----cuuaa--------------------------------ugCGGC--UAG-------------------------------------------CAGUCGc--------UagGG--GaU------------gCCUGU-aAaC--------------------------------------CCgaA----------------aCGACUGucag----auagaACAGGauc--------------------------------------GCCGcc---AaGUUc---GCUGUA--GACgU----aaCGGCuaaaa---cucaUACAGCuc--GCUCCaaGC----aCCCu---GCCA-CUCGGG--cgGCgc-GGAGUuaac------------------------------------------------ucaguaGAGc-UGGC-uaaGCA-UGUa----gaaCCg---aUAGcgg-----aaaGCUGGCGgac-GGGGGuucaaauCCCCC-CGGUUCCacca
Azot.vine.         GGGGCCGau-uaggauu-cgaCGCCG-GUaacgaa-acUUGa----GGg----GCAUGCCGAGUu---gGUGGCAG------aACUCGuaaau---ccaCUGCUACaaa-----------------cucauaguugccaacgacgacaacuac------------------------------------------------------gcuCUGGcGG----cuuaa--------------------------------ugCC-C--CAG-------------------------------------------CAGUCUC--------cagGG--GaU------------gCCUGU-aAaC--------------------------------------CCgaa----------------GAGACUGucag----auagaACAGGauc--------------------------------------GCCGcc---AaGUUc---GCUGUA--GACgU----aaCGGCuaaaa---cucaUACAGCuc--GCCCAaaGC----aCCCu---GCCC-GUCGGG--ccGCaa-UGGGUuaac------------------------------------------------ucaauaGACa-GGGC-uaaGCA-UGUa----gaaCCg---aUAGcgg-----agaGCUGGCGgac-GGGGGuucaaauCCCCC-CGGCUCCacca
Pseu.puti.         GGGGCCGau-uaggauu-cgaCGCCG-GUAgcgaa-acUcua----GGU----GCAUGCCGAGUu---gGUAACAG------aACUCGuaaau---ccaCUGUUGCaac----------------uuuuauaguugccaaugacgaaaacuac------------------------------------------ggcgcggaguacAAGCUgGCUG----cguaa----------gcaguccucuugacccguugcuucUGGU---AGCUUcggcuccag-------------------------------CAAUCAc--------UagGG--GaU------------gCCUGU-aAaC--------------------------------------CCgaA----------------cUGAUUGucau----acagaACAGGa--------------------------------------ucGUCGCgU--aGCaC----GUUGUG--G-GC---gAaGUGACuaaaacu---uaCACAACuc--AucCaaaGc----aCCCu---GCCc-GUCGGGc-ggcUgc-gGa-Uuaaa------------------------------------------------ucaguaGACa-cGGC-uaaGCA-UGUa----guACCg---acaGcgg-----agUACUGGCGgac-GGGGGuucaaauCCCCC-CGGCUCCacca
Pseu.fluo.-1.      GGGGCCGuu-uaggauu-cgaCGCCG-GUugcgaa-acUcua----GGU----GCAUGCCGACUu---gGUAACAG------aAGUCGuaaau---ccaCUGUUGCaac---------------uacuuauaguugccaaugacgaccaauac------------------------------------------------ggugcuGcucUcGCUG----cuuaa--------uugcagc-----ugaca-ugcacuccUGGU---AccuUcggguccag-------------------------------CAAUCAU--------cagGG--GaU------------gUCUGU-aAaC--------------------------------------CCaaa----------------AUGAUUGucau----auagaACAGAa--------------------------------------ucGCCGUgc--aGUaC----GUUGUG--G-AC---gaaGCGGCuaaaacu---uaCACAACuc--GCCCaaAGC----aCCCu---GCCc-GUCGGG--ucGCUg-aGGGUuaac------------------------------------------------uuaauaGACa-cGGC-uacGCA-UGUa----guACCg---acaGcgg-----aguACUGGCGgac-GGGGGuucaaauCCCCC-CGGCUCCacca
Pseu.fluo.-2.      GGGGCCGuu-uaggauu-cgaCGCCG-GUcgcgaa-acUUua----GGU----GCAUGCCGAGUu---gGUAACAG------aACUCGuaaau---ccaCUGUUGCaac---------------uucuuauaguugccaaugacgaaaacuac------------------------------------------ggccaggaauucGcucUcGCUG----cguaa----------gcagcucu---agccuugagcuucUGGU---AccuUcggguccag-------------------------------CAAUCAC--------cagGG--GaU------------gUCUGU-aAaC--------------------------------------CCaaa----------------GUGAUUGucau----auagaACAGAa--------------------------------------ucGCCGUgc--aGUaC----GUUGUG--G-AC---gaaGCGGCuaaaacu---uaCACAACuc--GCCCaaAGC----aCCCu---GCCc-UUCGGG--ucGCUg-aGGGUuaac------------------------------------------------uuaauaGAAa-cGGC-uacGCA-UGUa----guACCg---acAGcgg-----aguACUGGCGgac-GGGGGuucaaauCCCCC-CGGCUCCacca
Pseu.syri.-1.      GGGGCCGau-uuggauu-cgaCGCCG-GUGaugaa-acUUua----GGU----GCAUGCCGAGUu---gGUAACAG------aACUCGuaaau---ccaCUGUUGCaac---------------uuucuauaguugccaaugacgaaaacuac------------------------------------------------ggugcucAaCUaGCUG----cguaa----------gcagc-cu---agcc--gcacuucUGGU---AGcUucggcuccag-------------------------------CAAUCAU--------cagGG--GaU------------gCCUGU-aAaC--------------------------------------CCaaa----------------AUGAUUGucau----auagaACAGGa--------------------------------------ucGUCGCcU--aGUaC----GUUGUG--G-AC---gAaGCGACuaaaacu---uaCGCAACuc--GCCCaauGC----aCCCu---GCCc-GUCGGG--ucGCug-aGGGUuaac------------------------------------------------uuaauaGACa-cGGC-uacGCA-UGUa----guACCg---acAGcgg-----agUACUGGCGgac-GGGGGuucaaauCCCCC-CGGCUCCacca
Pseu.syri.-2.      GGGGCCGau-uuggauu-cgaCGCCG-GUGaugaa-acUUua----GGU----GCAUGCCGAGUu---gGUAACAG------aACUCGuaaau---ccaCUGUUGCaac---------------uuucuauaguugccaaugacgaaaccuac---------------------------------------------ggggaauacGcuCUcGCCG----cguaa----------gcggugcu---agcc--uuccuucUGGU---AGcuUcggcuccag-------------------------------CAAUCAU--------cagGG--GaU------------gCCUGU-aAaC--------------------------------------CCgaa----------------AUGAUUGucau----auagaACAGGa--------------------------------------ucGUCGCcU--aGUaC----GUUGUG--G-AC---gAaGCGACuaaaacu---uaCACAACuc--GCCCaaAGC----aCCCu---GCCc-GUCGGG--ucGCUg-aGGGUuaac------------------------------------------------uuaauaGACa-cGGC-uaaGCA-UGUa----guACCg---acAGcgg-----agUACUGGCGgac-GGGGGuucaaauCCCCC-CGGCUCCacca
Mari.hydr.         nnnnnnnnnnnnnnnnn-nnnnGCCG-GUGacgaa-cCCUUG----GGU----GCAUGCCGAGAu---gGCAGCga------aUCUCGuaaau---ccaaaGCUGCaac-----------------guaauagucgcaaacgacgaaaacuac------------------------------------------------------GCacuGGCGG----cguaa---------------------------------gCCGU--UccaGU----------------------------------------CGUCCUG--------GCUGa--GGc------------GCCUAU-aaCUc------------------------------------agUAGCaacauc---------cCAGGACGucauc----gcuuAUAGGCu---------------------------------------GCUccGuU-cACCAga--GCUCac--UGGU---GuU-cGGCuaaga----uuaaaGAGCuc--GCCUCuuGC----aCCCu---Gacc-UUcGGG--ucGCuu-GAGGUuaaa-----------------------------------------------ucaauagaAGg-acaC-uaaGCA-UGUa----g-ACCu---CAAGGccu----agUGCUGGCggac-gcggnnnnnnnnnnnnn-nnnnnnnnnnn
Pseu.halo.         nnnnnnnnnnnnnnnnn-nnnnGGAA-uuCaagaa--GcCCG---aGGU----GCAUGUCGAGGu----GCGGUUU------gCCUCGuaaa------aAAGCCGCaauuu---------------aaaguaaucgcaaacgacgauaacuac------------------------------------------------------ucuCUAGCaG----cuuag--------------------------------gcUgGC--UAG-------------------------------------------CGCUCCu--------UCCAu--GUaU-----------UCuuGU-GgAC---------------------------------------UGGAuuu-------------uGGAGUGucacc---cuaacACcuGAuc--------------------------------------GCGaCgg--aAACCcu--GGCCGg--GGUU---gaaG-CGUuaaaa---cuaagCGGCCuc--GCCUUUAucU---aCCGu---GUuU-GUCCGG--gAuuUA-AAGGUuaa-------------------------------------------------uuaaauGACa-AuAC-uaaACA-UGUa----guACCg--aCGGuCg------agGcuUUUCggac-ggggnnnnnnnnnnnnn-nnnnnnnnnnn
Uncu.ww11.         nnnnnnnnnnnnnnnnnnnnaCAGGA-UuCacgaa-accCUG----GGa----GCAUGCCGAGG----gGCGGUUg------gCCUCGuaaa-------aAGCCGCaca-----------------guuauaguugcaaacgacgauaacuac------------------------------------------------------gcuCUAGCaG----cuuag--------------------------------gcUaGC--UAG-------------------------------------------CCAUCUG--------ACACa-gGUCU----------cUCAAAU-GGGCg--------------------------------------GUGUau-------------aCAGAUGGucauc---uuac-AUUUGAua--------------------------------------GCGaGgg---AaCUcc--GUCCGG--GGgU---gaaC-CGCgaaau---aguaCCGGACuc--GCCCGAugAA---aUCCu---GUCU-UUCGGA-guUUauU-CGGGUuaau-------------------------------------------------uaaaaGAG--AGAC-uaaGCA-UGUa----gugCCu---UGGaugu-----agGuGUUCUGgac-gcgnnnnnnnnnnnnnn-nnnnnnnnnnn
Shew.putr.         GGGGGCGauucuggauu-cgaCAGGA-uuCacgaa-accCUG----GGa----GCAUGCCGAGG----gGCGGUUg------gCCUCGuaaa-------aAGCCGCaaa-----------------guuauaguugcaaacgacgauaacuac------------------------------------------------------gcuCUAGCcG----cuuaa--------------------------------ugCcGC--UAG-------------------------------------------CCAUCuA--------CCACa-cGCUU----------uGCACau-GGGCa--------------------------------------GUGGau--------------UuGAUGGucauc---ucacaucGUGCua--------------------------------------GCGaGgg---AaCCcu--GUCUGG--GGgU---gaaC-CGCgaaac---aguaCCGGACuc--ACCGUgUGGG---aUCCu---GUCU-UUCGGA--gUUCAa-ACGGUuaaa-------------------------------------------------caauaGAA--AGAC-uaaGCA-UGUa----gcgCCu---UGGaugu-----agGuuUUCUGgac-GCGGGuucaaguCCCGC-CGCCUCCacca
Colw.spec.         GGGGCGGaucuaggauu-cgaCAAGA-uuCaugaa--acUCa---aGGU----GCAUGCCCAGG----gGCGGUUU------gCCUGGuaaa-------AAGCCGCaa-----------------aacuauaauugcuaacgacgauacguuc------------------------------------------------------gcaCuAGCcG----cuuag--------------------------gcuagccaUcGC-cUuGaaa----------------------------------------UCucUcc--------uauug-----------------gUUaG--agga-----------------------------------------------------------ucGauGGucac----cccaaaUaGGaua--------------------------------------GCGagg--gaAGCac---GCUUGA--gGCU---gaa-cCGCgaaau---aguaUCAAGCuca-cCauGacGaA----GCCu---GUCG-CUUGGC--gUcUaa-UugGuuaa-------------------------------------------------auaaauGAG--CGAC-uaaGCA-UGUa----guACCg---aGAacgu-----agGcuUUUUGgac-GGGGGuucgauuCCCCC-CCGCUCCacca
Phot.phos.         nnnnnnnnnnnnnnnnnnnnnnAGGA--Ucacgaa-gGCuuG---UGGa----GCAUGUCGAGGu----GCGGUUu------gCCUCGuuaa------acAGCCGCaaa-----------------aaaauagucgcaaacgacgaaaacuac------------------------------------------------------GCaCUaGCAG----cuuaauac------------------------------cCUGC-uaAGaGC----------------------------------------GCUCCUG--------CCCUa--gCUUcc---------gCUUGU-AAGa--------------------------------------cGGGGaaua-----------aCAGGGGUucaaa---cccaaACGAGaua--------------------------------------GCGaGgg-auUCUUug--ACUAGA--GAGAu--gaaC-CGCgaaau--agaauUCUGGUau--GUAUUagCAU---aGCGu---GUCA-AUUCGC--aGUGac-GAUACgaga-------------------------------------------------auaaaGAU--UGAC-uaaACA-UGUa----gcgCCA---UcuGUuag----acuGaUUUUggac-gcggnnnnnnnnnnnnn-nnnnnnnnnnn
Vibr.chol.         GGGGCUGauucaggauu-cgaCGGGA-AUUuugca--GUCUg---aGGU----GCAUGCCGAGGu----GCGGUag------gCCUCGuuaa------caaACCGCaaa-----------------aaaauagucgcaaacgacgaaaacuac------------------------------------------------------GCaCUaGCAG----cuuaauac------------------------------cCUGC-ucAGaGC----------------------------------------CCUUCCU--------CCCUa--gCUUcc---------GCUUGu-AAGa--------------------------------------cGGGGaaau-----------cAGGAAGGucaaa---ccaaauCAAGCug--------------------------------------GCGUgg--auUCCCcc--ACCUGA--GGGAu--gaa-GCGCgagau--cuaauUCAGGUua--GCCAUUcGUU---aGCGu---GUCG-GUUCGC--aGGC-G-GUGGUgaaa-------------------------------------------------uuaaaGAU--CGAC-uaaGCA-UGUa----guACCa---aAGAUga-----auGGUUUUCGgac-GGGGGuucaacuCCCCC-CAGCUCCacca
Aero.salm.         nnnnnnnnnnnnnnnnn-nnnnAAGA-UUcacgaa--AcCCa---aGGU----GCAUGCCGAGGu----GCGGUag------gCCUCGuuaa------caaACCGCaaa-----------------aaaauagucgcaaacgacgaaaacuac------------------------------------------------------GCaCUAGCAG----cuuaauaa------------------------------cCUGC-aUAGaGC----------------------------------------CCUUCUa--------CCCUA--GcuU-----------gCCUGU-GucC--------------------------------------UAGGGaau-------------cGGAAGGucau----ccuucACAGGauc--------------------------------------GUGUgg---aaGUCcu--GCUCGG--GGCg---gaa-GCAUuaaaa---ccaaUCGAGCua--GUCAAUUCGU---gGCGu---GUCu-CUCCGC--aGCGGG-UUGGCgaau-------------------------------------------------guaaaGAG--uGAC-uaaGCA-UGUa----guACCg---aGGaUgu-----aguAAUUUUggac-ggggnnnnnnnnnnnnn-nnnnnnnnnnn
Uncu.vlw3.         nnnnnnnnnnnnnnnnn-nnaCAAGA-UUcacgaa--AcCCa---aGGU----GCAUGCCGAGGu----GCGGUag------gCCUCGuuaa------caaACCGCaaa-----------------aaaauagucgcaaacgacgaaaacuac------------------------------------------------------GCaCucGCAG----cuuaauaa------------------------------cCUGC-gcuGaGC----------------------------------------CCUUCUa--------CCCUA--GcuU-----------gCCUGU-GucC--------------------------------------UAGGGaau-------------cGGAAGGucau----ccuucACAGGauc--------------------------------------GUGUgg---aaGUCcu--GCUCGG--GGCg---gaa-GCAUuaaaa---ccaaUCGAGCua--GUCAAUUCGU---gGCGu---GUCu-CUCCGC--aGCGGG-UUGGCgaau-------------------------------------------------guaaaGAG--uGAC-uaaGCA-UGUa----guACCg---aGGaUgu-----aguAAUUUUGgac-gggnnnnnnnnnnnnnn-nnnnnnnnnnn
Uncu.vlw4.         nnnnnnnnnnnnnnnnn-nnaCAAGA-UUcacgaa--AcCCa---aGGU----GCAUGCCGAGGu----GCGGUag------gCCUCGuuaa------caaACCGCaaa-----------------aaaauagucgcaaacgacgaaaacuac------------------------------------------------------GCaCucGCAG----cuuaauaa------------------------------cCUGC-gcuGaGC----------------------------------------CCUUCUa--------CCCUA--GcuU-----------gCCUGU-GucC--------------------------------------UAGGGaau-------------cGGAAGGucau----ccuucACAGGauc--------------------------------------GUGUgg---aaGUCcu--GCUCGG--GGCg---gaa-GCAUuaaaa---ccaaUCGAGCua--GUCAAUUCGU---gGCGu---GUCu-CUCCGC--aGCGGG-UUGGCgaau-------------------------------------------------guaaaGAG--uGAC-uaaGCA-UGUa----guACCg---aGGaUgu-----aguAAUUUUGgac-ggggguunnnnnnnnnn-nnnnnnnnnnn
Uncu.vls1.-3.      nnnnnnnnnnnnnnnnn-nnaCAAGA-UUcacgaa--AcCCa---aGGU----GCAUGCCGAGGu----GCGGUag------gCCUCGuuaa------caaACCGCaaa-----------------aaaauagucgcaaacgacgaaaacuac------------------------------------------------------GCaCucGCAG----cuuaauaa------------------------------cCUGC-gcuGaGC----------------------------------------CCUUCUa--------CCCUA--GcuU-----------gCCUGU-GucC--------------------------------------UAGGGaau-------------cGGAAGGucau----ccuucACAGGauc--------------------------------------GUGUgg---aaGUCcu--GCUCGG--GGCg---gaa-GCAUuaaaa---ccaaUCGAGCua--GUCAAUUCGU---gGCGu---GUCu-CUCCGC--aGCGGG-UUGGCgaau-------------------------------------------------guaaaGAG--uGAC-uaaGCA-UGUa----guACCg---aGGaUgu-----aguAAUUUUGgac-gggnnnnnnnnnnnnnn-nnnnnnnnnnn
Uncu.rca3.         nnnnnnnnnnnnnnnnn-nnaCAAGA-UUcacgaa--AcCCa---aGGU----GCAUGCCGAGGu----GCGGUag------gCCUCGuuaa------caaACCGCaaa-----------------aaaauagucgcaaacgacgaaaacuac------------------------------------------------------GCaCucGCAG----cuuaauaa------------------------------cCUGC-gcuGaGC----------------------------------------CCUUCUa--------CCCUA--GcuU-----------gCCUGU-GucC--------------------------------------UAGGGaau-------------cGGAAGGucau----ccuucACAGGauc--------------------------------------GUGUgg---aaGUCcu--GCUCGG--GGCg---gaa-GCAUuaaaa---ccaaUCGAGCua--GUCAAUUCGU---gGCGu---GUCu-CUCCGC--aGCGGG-UUGGCgaau-------------------------------------------------guaaaGAG--uGAC-uaaGCA-UGUa----guACCg---aGGaUgu-----aguAAUUUUGgac-gggnnnnnnnnnnnnnn-nnnnnnnnnnn
Uncu.vls1.-4.      nnnnnnnnnnnnnnnnn-nnaCAAGA-UUcacgaa--AcCCa---aGGU----GCAUGCCGAGGu----GCGGUag------gCCUCGuuaa------caaACCGCaaa-----------------aaaauagucgcaaacgacgaaaacuac------------------------------------------------------GCaCucGCAG----cuuaauaa------------------------------cCUGC-gcuGaGC----------------------------------------CCUUCUa--------CCCUA--GcuU-----------gCCUGU-GucC--------------------------------------UAGGGaau-------------cGGAAGGucau----ccuucACAGGauc--------------------------------------GUGUgg---aaGUCcu--GCUCGG--GGCg---gaa-GCAUuaaaa---ccaaUCGAGCua--GUCAAUUCGU---gGCGu---GUCu-CUCCGC--aGCGGG-UUGGCgaau-------------------------------------------------guaaaGAG--uGAC-uacGCA-UGUa----guACCg---aGGaUgu-----aguAAUUUUGgac-gggnnnnnnnnnnnnnn-nnnnnnnnnnn
Uncu.ww-9.         nnnnnnnnnnnnnnnnn-nnaCAAGA-UUcacgaa--AcCCa---aGGU----GCAUGCCGAGGu----GCGGUag------gCCUCGuuaa------caaACCGCaaa-----------------aaaauagucgcaaacgacgaaaacuac------------------------------------------------------GCaCucGCAG----cuuaauaa------------------------------cCUGC-gcuGaGC----------------------------------------CCUUCUa--------CCCUA--GcuU-----------gCCUGU-GucC--------------------------------------UAGGGaau-------------cGGAAGGucau----ccuucACAGGauc--------------------------------------GUGUgg---aaGUCcu--GCUCGG--GGCg---gaa-GCAUuaaaa---ccaaUCGAGCua--GUCAAUGCGU---gGCGu---GUCu-CUCCGC--aGCGUG-UUGGCgaau-------------------------------------------------guaaaGAG--uGAC-uaaGCA-UGUa----guACCg---aGGaUgu-----aguAAUUUUGgac-gggnnnnnnnnnnnnnn-nnnnnnnnnnn
Uncu.ww10.         nnnnnnnnnnnnnnnnn-nnaCAAGA-UUcacgaa--AcCCa---aGGU----GCAUGCCGAGGu----GCGGUag------gCCUCGuuaa------caaACCGCaaa-----------------aaaguagucgcaaacgacgaaaacuac------------------------------------------------------GCaCucGCAG----uuuaauaa------------------------------cCUGC-gcuGaGC----------------------------------------CCUUCUa--------CCCUA--GcuU-----------gCCUGU-GucC--------------------------------------UAGGGaau-------------cGGAAGGucau----ccuucACAGGauc--------------------------------------GUGUgg---aaGUCcu--GCUCGG--GGCg---gaa-GCAUuaaaa---ccaaUCGAGCua--GUCAAUGCGU---gGCGu---GUCu-CUCCGC--aGCGUG-UUGGCgaau-------------------------------------------------guaaaGAG--uGAC-uaaGCA-UGUa----guACCg---aGGaUgu-----aguAAUUUUGgac-gggnnnnnnnnnnnnnn-nnnnnnnnnnn
Uncu.vlw5.         nnnnnnnnnnnnnnnnn-nngCAAGA-UUcacgaa--A-CCa---aGGU----GCAUGCCGAGGu----GCGGUag------gCCUCGuuaa------caaACCGCaaa-----------------aaaauagucgcaaacgacgaaaacuac------------------------------------------------------GCaCucGCAG----cuuaauaa------------------------------cCUGC-gcuGaGC----------------------------------------CCUUCUc--------CCCUA--GcuU-----------gCCUGU-GucC--------------------------------------UAGGGaau-------------uGGAAGGucau----ccuucACAGGauc--------------------------------------GUGUgg---aaGUCcu--GCUCGG--GGCg---gaa-GCAUuaaaa---ccaaUCGAGCua--GU-AAUGCGU---gGCGu---GUCu-CUCCGC--aGCGUG-UUgGCgaau-------------------------------------------------guaaaGAG--uGAC-uaaGCA-UGUa----guACCg---aGGaUgu-----aguAAUUUUGgac-gggnnnnnnnnnnnnnn-nnnnnnnnnnn
Uncu.vlw6.         nnnnnnnnnnnnnnnnn-nnaCAAGA-UUcacgaa--AcCCa---aGGU----GCAUGCCGAGGu----GCGGUag------gCCUCGuuaa------caaACCGCaaa-----------------aaaauagucgcaaacgacgaaaacuac------------------------------------------------------GCaCucGCAG----cuuaauaa------------------------------cCUGC-gcuGaGC----------------------------------------CCUUCUc--------CCCcA--GcuU-----------gCCUGU-GucC--------------------------------------UaGGGaau-------------uGGAAGGucau----ccuucACAGGauc--------------------------------------GUGUgg---aaGUCcu--GCUCGG--GGCg---gaa-GCAUuaaaa---ccauUCGAGCua--GUCAAUGCGU---gGCGu---GUCu-CUCCGC--aGCGUG-UUGGCgaau-------------------------------------------------guaaaGAG--uGAC-uaaGCA-UGUa----guACCg---aGGaUgu-----aguAAUUUUGgnn-nnnnnnnnnnnnnnnnn-nnnnnnnnnnn
Uncu.rca4.         nnnnnnnnnnnnnnnnn-nnaCGGGA-cACucgaa--AcCCa---aGGU----GCAUGCCGAGGu----GCGGUag------gCCUCGuuaa------uaaACCGCaa------------------aaaauagucgcaaacgacgaaaccuac------------------------------------------------------GCUUUaGCAG----cuuaauaa------------------------------cCUGC-ucGGAGC----------------------------------------CCUUCUA--------CCCUA--GcuU-----------gCCUAU-GucC--------------------------------------UAGGGgau-------------UGGAAGGucau----cuuucAUAGGauc--------------------------------------GCGUgg---aaACCuu--GUCUGG--GGUg---gaa-GCGUuaaaa---cuaaUCAGACua--GUUAUUCAGU---gGCGu---GUCG-GUCCGC--aGCUGG-AUGGUgaau-------------------------------------------------guaaaGAUu-CGAC-uaaGCA-UGUa----guACUg---aGGaUgu-----agGUuUUUCGgac-gcgnnnnnnnnnnnnnn-nnnnnnnnnnn
Uncu.rca7.         nnnnnnnnnnnnnnnnn-nnaCGGGA-cACucgaa--AcCCa---aGGU----GCAUGCCGAGGu-----CGGUag------gCCUCGuuaa------uaaACCGcaa------------------aagauagccgcaaacgacgaaaccuac------------------------------------------------------GCUUUaGCAG----cuuaauaa------------------------------cCUGC-ucGGAGC----------------------------------------CCUUCUA--------CCCUA--GcuU-----------gCCUAU-GucC--------------------------------------UAGGGgau-------------UGGAAGGucau----cuuucAUAGGauc--------------------------------------GCGUgg---aaACCuu--GUCUGG--GGUg---gaa-GCGUuaaaa---cuaaUCAGACua--GUUAUUCAGU---gGCGu---GUCG-GUCCGC--aGCUGG-AUGGUgaau-------------------------------------------------guaaaGAUu-CGAC-uaaGCA-UGUa----guACUg---aGGaUgu-----agGUuUUUCGgac-gcgnnnnnnnnnnnnnn-nnnnnnnnnnn
Uncu.rca8.         nnnnnnnnnnnnnnnnn-nnaCGGGA-cACucgaa--AcCCa---aGGU----GCAUGCCGAGGu----GCGGUag------gCCUCGuuaa------uaaACCGCaa------------------aaaauagucgcaaacgacgaaaccuac------------------------------------------------------GCUUUaGCAG----cuuaauaa------------------------------cCUGC-ucGGAGC----------------------------------------CCUUCUA--------CCCUA--GcuU-----------gCCUAU-GucC--------------------------------------UAGGGgau-------------UGGAAGGucau----cuuucAUAGGauc--------------------------------------GCGUgg---aaGCCuu--GUCUGG--GGUg---gaa-GCGUuaaaa---cuaaUCAGACua--GUUAUUCAGU---gGCGu---GUCG-GUCCGC--aGCUGG-GUGGCgaau-------------------------------------------------guaaaGAUu-CGAC-uaaGCA-UGUa----guACUg---aGGaUgu-----agGUuUUUCGgac-gcnnnnnnnnnnnnnnn-nnnnnnnnnnn
Uncu.lem1.         nnnnnnnnnnnnnnnnn-nnaCGGGA-cACucgaa--AcCCa---aGGU----GCAUGCCGAGGu----GCGGUag------gCCUCGuuaa------uaaACCGCaa------------------aaaauagucgcaaacgacgaaaccuac------------------------------------------------------GCUUUaGCAG----cuuaauaa------------------------------cCUGC-ucGGAGC----------------------------------------CCUUCUA--------CCCUA--GcuU-----------gCCUAU-GucC--------------------------------------UAGGGgau-------------UGGAAGGucau----cuuucAUAGGauc--------------------------------------GCGUgg---aaGCCuu--GUCUGG--GGUg---gaa-GCGUuaaaa---cuaaUCAGACua--GUUAUUCAGU---gGCGu---GUCG-GUCCGC--aGCUGG-GUGGCgaau-------------------------------------------------guaaaGAUu-CGAC-uaaGCA-UGUa----guACUg---aGGaUgu-----agGUuUUUCGgac-gcgnnnnnnnnnnnnnn-nnnnnnnnnnn
Uncu.lem2.         nnnnnnnnnnnnnnnnn-nnaCGGuA-cacuagaa--AaCCa---aGGU----GCAUaCCGAGGu----GCGGUag------gCUUCGuuaa------uaaACCGCaa-------------------aagauagucgcaacgacgaaacgcac------------------------------------------------------GcUUUaGCAG----cuuaauau------------------------------gCUGC-uaGGAcU----------------------------------------CCUUcUn--------naCUA--GcuU-----------gcUUAU-GucC--------------------------------------UAGgggau-------------uGaAAGGucau----cuuucAUAGagac--------------------------------------GCGUgg---aaGCCcu--GUCUGG--GGUg---gaa-GCGUcaaaa---cuaaUCAGGCaa--GUUAUgCAaU---ggCGu---GgCG-GaCCGg--aGcUGg-GUGGCgaau-------------------------------------------------ggaaaGaUu-CGaC-uaaGcA-UGUa----guACUg---aGGaUgu-----aguauUuUCGgac-gcggguucaannnnnnn-nnnnnnnnnnn
Past.mult.         GGGGCUGauucuggauu-cgaCGGGA-UUAgcgaa--GCCca---aGGU----GCACGUCGAGGu----GCGGUag------gCCUCGuaaa------uaaACCGCaaa-----------------aaaauagucgcaaacgacgaacaauac------------------------------------------------------GCUuUaGCAG----cuuaauaa------------------------------cCUGC-ucAuAGC----------------------------------------CUUCUCU--------CCCCa--gCUUcc---------gCUCGU-AAGa--------------------------------------cGGGGauca------------AGAGGAGucaaa---uucaaACGAGauc--------------------------------------GCGUgg---acgCCucc-GCUUGA--GGauc--gaa-GCGUuaaa---uugaaUCAAGCua--GUUUAUCUAU---cGCGu---GUCU-GUCCGC--aGUGGG-UAAAUgaaa-------------------------------------------------uuaaaGACg-AGAC-uaaACG-UGUa----guACUg---aaGGUag-----agUAAUUUCGgac-GCGGGuucaacuCCCGC-CAGCUCCacca
Mann.haem.         GGGGCUGauuuuggauu-cgaCGGGA-UUggcgaa--GCCca---aGGU----GCAUGUCGAGGu----GCGGUag------gCCUCGuaaa------caaACCGCaaa-----------------auaauagucgcaaacgacgaacaauac------------------------------------------------------GCUUUAGCAG----cuuaauaa------------------------------cCUGCuuUAAAGC----------------------------------------CUUAUCU--------CCUCa--gCUUcc---------gCUCGU-AAGa--------------------------------------cGAGGgcaa------------AGAUAAGucac----ccaaaACGAGauc--------------------------------------GUGUgg---acgCCgcc-GCUUUA--GGauc--gaa-ACACuaaa---uuaaaUAAAGCua--GUUUAUUCAU---uGCGu---GUCU-GUCCGC--uGUGAA-UAAGCgaaa-------------------------------------------------uuaaaGACu-AGAC-uaaACA-UGUa----guACUg---aaGGUag-----agaAAUUUCGgac-GGGGGuucaaauCCCCC-CAGCUCCacca
Haem.somn.         GGGGCUGauuuuggauu-cgaCGGAA-UUAgcgaa--GCCca---aGGU----GCACGUCGAGGu----GCGGUag------gCCUCGuaaa------uaaACCGCaaa-----------------acaauagucgcaaacgacgaacaauac------------------------------------------------------GCUuUaGCAG----cuuaauaa------------------------------cCUGC-ucAuAGC----------------------------------------CUUCACU--------CCCUa--gCUUuc---------gCUCGU-AAGa--------------------------------------cGGGGauaa------------AGUGGAGucaaa---uccaaACGAGauc--------------------------------------GUGUug---aagCCgcc-GCUUUA--GGauc--gaa-GCAUuaaa---uugaaUAAAGCua--GUuuuUUGAU---gGCGu---GUCU-GUCCGC--aGUCAA-uuuACgaaa-------------------------------------------------uuaaaGAUu-AGAC-uaaACG-UGUa----ggACUg---aaGGUag-----agUAAUUUCGgac-GGGGGuucaacuCCCCC-CAGCUCCacca
Haem.infl.         GGGGCUGauucuggauu-cgaCGGGA-UUAgcgaa--GCCca---aGGU----GCACGUCGAGGu----GCGGUag------gCCUCGuaaa------uaaACCGCaaa-----------------aaaauagucgcaaacgacgaacaauac------------------------------------------------------GCUuuAGCAG----cuuaauaa------------------------------cCUGC-aUuuAGC----------------------------------------CUUCGCG--------CUCCa--gCUUcc---------gCUCGU-AAGa--------------------------------------cGGGGauaa------------CGCGGAGucaaa---ccaaaACGAGauc--------------------------------------GUGUgg---aAgCCacc-GUUUGA--GGaUc--gaa-GCACuaaa---uugaaUCAAACua--GCUUAAGUuU---aGCGu---GUCU-GUCCGC---AuGCU-UAAGUgaaa-------------------------------------------------uuaaaGACg-AGAC-uaaACG-UGUa----guACUg---aaGGUag-----agUAAUUUCGgac-GGGGGuucaacuCCCCC-CAGCUCCacca
Haem.ducr.         GGGGCUGauucuggauu-cgaCGGGA-UUAgcgaa--GUCca---aGGU----GCACGUCGAGGu----GCGGUag------gCCUCGuaaa------caaACCGCaaa-----------------aaaauagucgcaaacgacgaacaauac------------------------------------------------------GCUuuAGCAG----cuuaauaa------------------------------cCUGC-aUuuAGC----------------------------------------CUUCGCG--------CCCUa--gCUUuc---------gCUCGU-AAGa--------------------------------------cGGGGagca------------CGCGGAGucaaa---ccaaaACGAGauc--------------------------------------GUGUgg---acgCUucg-GCUUGu--AGauc--gaa-ACACcaaa---uugaauCAAGCua--GUUUAUUUCU---uGCGu---GUCU-GUCCGC--uGGAGA-UAAGCgaaa-------------------------------------------------uuaaaGACc-AGAC-uaaACG-UGUa----guACUg---aaGAUag-----agUAAUUUCGgac-GGGGGuucaaauCCCCC-CAGCUCCacca
Acti.pleu.         GGGGCUGauucuggauu-cgaCGGGA-UUAgcgaa--GUCca---aGGU----GCACGUCGAGGu----GCGGUag------gCCUCGuaaa------caaACCGCaaa-----------------aaaauagucgcaaacgacgaacaauac------------------------------------------------------GCUuUaGCAG----cuuaauaa------------------------------cCUGC-ucAuAGC----------------------------------------CUUAUCG--------CCUCa--gCUUcc---------gCUCGU-AAGa--------------------------------------cGAGGgcaa------------CGAUAAGucac----ccaaaACGAGauc--------------------------------------GUGUgg---acgCCgcc-GUUUGA--GGauc--gaa-ACACuaaa---uugaaUCAAACua--GCUUAUUUCU---uGCGu---GUCU-GUCCGC--uGGAGG-UAAGUgaaa-------------------------------------------------uuaaaGACc-AGAC-uaaACG-UGUa----guGCUg---aaGAUgg-----agUAAUUUCGgac-GGGGGuucaaauCCCCC-CAGCUCCacca
Acti.acti.         GGGGCUGauucuggauu-cgaCGGGA-UUagcgaa--GCCcg---aAGU----GCACGUCGAGGu----GCGGUag------gCCUCGuaaa------uaaACCGCaaa-----------------aaaauagucgcaaacgacgaacaauac------------------------------------------------------GCUuuAGCAG----cuuaauaa------------------------------cCUGC-cUuuAGC----------------------------------------CUUCGCU--------CCCCa--gCUUcc---------gCUCGU-AAGa--------------------------------------cGGGGauaa------------AGCGGAGucaaa---ccaaaACGAGauc--------------------------------------GUGUgg---aAgCCacc-gUUUGA--GGaUc--gaa-GCAUuaaa---uuaaaUCAAAgua--GCUUAAUUgU---cGCgu---GUCC-GUCaGC---AgGAU-UAAGUgaau-------------------------------------------------uuaaaGACc-GGAC-uaaACG-UGUa----guGCUa---acGGCag-----aggAAUUUCGgac-GGGGGuucaacuCCCCC-CAGCUCCacca
Buch.aphi.         GGGGCUGuuuuuggauu-cgaCAAAG-UUaucaaa-aAAGUA---AaGU----GCAUGCCGAGG----aACGGUUU------gCCUCGauaa------aAAACCGUaaa-----------------aaaauagcugcaaauaauaaacaaaac---------------------------------------------------uacGCUUUAGCAG----cuuaaaaa------------------------------aCUGU-aUAAAGC----------------------------------------CCUUUCa--------UCUCu-gaCUUc----------UCUCUU-GAGaa-------------------------------------cGAGAaauuu--------uuaaGAAAGGccgac---auuuaAAGAGAaaa-------------------------------------aUGUGag--CACUA--u-GCUUGA--UAGUG----aCACAaaauua--auuuuUCAAGCua--uaaGCUUAAa----aUGa---GUUU-UUCCAa---aUUAA-GCaaugaaaaaa-----------------------------------------------uaaaGAA--AAAC-uaaGCA-UGUa----guAUcUu--UAUUUau-----agaAAUUUUGgac-GCGGGuucaaauCCCGC-CAGCUCCacca
Buch.APS-.         GGGGCUGuuuuuggauu-ugaCAAAA-UUaucaac-aaAGUA---AAGU----GCAUGCCGAGG----aACGGUUU------gCCUCGauaa-------AAGCCGUaaa-----------------aaaauaauugcaaauaauaaacaaaac---------------------------------------------------uacGCUUUAgCAG----cuuaaaaa------------------------------aCUGa-aUAAAGC----------------------------------------CCUUCCU--------UCUUA--UCCUcc--------uUCUCUU-AGGA--------------------------------------UGAGAaaac------------AGGAAGGucaga------aaAAGAGAaaa-------------------------------------aCGUGg--aUAUCa--a-GCUUGA--cGAUA----aCACGaaguag--uuuuuUCAAGCua--uaUACCUAAU----aUGu---GUUU-UUCCAa---AUUAG-GUAaugaau----------------------------------------------ucaauaaaGAAa-AAAC-uaaGCA-UGUa----ggGCUU---UAUUagu-----agaAAUUUUGgac-GCGGGuucgaauCCCGC-CAGCUCCacca
Esch.coli.-1.      GGGGCUGauucuggauu-cgaCGGGA-UUUgcgaa--AcCCa---aGGU----GCAUGCCGAGG----gGCGGUUg------gCCUCGuaaa-------aAGCCGCaa------------------aaaauagucgcaaacgacgaaaacuac------------------------------------------------------GCUUUAGCAG----cuuaauaa------------------------------cCUGC-uUAGAGC----------------------------------------CCUCUCU--------CCCUa--gCCUcc---------gCUCUU-AGGa--------------------------------------cGGGGauca------------AGAGAGGucaaa---cccaaAAGAGauc--------------------------------------GCGUgg---aAGCCcu--GCCUGG--GGUU---gaa-GCGUuaaaa--cuuaaUCAGGCua--GUUUGUUAGU---gGCGu---GUCc-GUCCGC--aGCUGG-CAAGCgaau-------------------------------------------------guaaaGAC--uGAC-uaaGCA-UGUa----guACCg---aGGaUgu-----agGAAUUUCGgac-GCGGGuucaacuCCCGC-CAGCUCCacca
Esch.coli.-2.      GGGGCUGauucuggauu-cgaCGGGA-UUUgcgaa--AcCCa---aGGU----GCAUGCCGAGG----gGCGGUUg------gCCUCGuaaa-------aAGCCGCaa------------------aaaauagucgcaaacgacgaaaacuac------------------------------------------------------GCUUUAGCAG----cuuaauaa------------------------------cCUGC-uUAGAGC----------------------------------------CCUCUCU--------CCCUa--gCCUcc---------gCUCUU-AGGa--------------------------------------cGGGGauca------------AGAGAGGucaaa---cccaaAAGAGauc--------------------------------------GCGUgg---aAGCCcu--GCCUGG--GGUU---gaa-GCGUuaaaa--cuuaaUCAGGCua--GUUUGUUAGU---gGcGu---GUcc-GUCCaC--aGCUGG-CAAGCgaau-------------------------------------------------guaaaGAC--urAC-uaaGCA-UGUa----guACCg---aGGaUgu-----agAAAUUUCGgac-GCGGGuucaacuCCCGC-CAGCUCCacca
Esch.coli.-3.      GGGGCUGauucuggauu-cgaCGGGA-UUUgcgaa--acCCa---aGGU----GCAUGCCGAGG----gGCGGUUg------gCCUCGuaaa-------aAGCCGCaa------------------aaaauagucgcaaacgacgaaaacuac------------------------------------------------------GCUUUAGCAG----cuuaauaa------------------------------cCUGC-uUAGAGC----------------------------------------CCUCUCU--------CCCUa--gCCUcc---------gCUCUU-AGGa--------------------------------------cGGGGauca------------AGAGAGGucaaa---cccaaAAGAGauc--------------------------------------GCGUgg---aAGCCcu--GCCUGG--GGUU---gaa-GCGUuaaaa--cuuaaUCAGGCua--GUUUGUUAGU---gGCGu---GUCc-GUCCGC--aGCUGG-CAAGCgaau-------------------------------------------------guaaaGAC--uGAC-uaaGCA-UGUa----guACCg---aGGacgu-----agGAAUUUCGgac-GCGGGuucaacuCCCGC-CAGCUCCacca
Prov.rett.         nnnnnnnnnnnnnnnnn-nnnnGGGA-UUUgcgaa--AcCCa---aGGU----GCAUGCCGAGG----gGCGGUUg------gCCUCGuaaa-------aAGCCGCaaa-----------------aaaauagucgcaaacgacgaaaacuac------------------------------------------------------GCUUUAGCAG----cuuaauaa------------------------------cCUGC-uUAGAGC----------------------------------------CCUCUCU--------CCCUa--gcCUcc---------gCUCUU-GGa---------------------------------------cGGGGauca------------AGAGAGGucaaa---cccaaAAGAGauc--------------------------------------GCGUgg---auGCCuu--gCCUGG--GGUu---gaa-GCGUuaaa---cuuaaUCAGGaua--GUUUGUUGGU---gGCGu---GUCU-GUCCGC--aGCUGG-CAAAUgaau-------------------------------------------------ucaaaGAC-uAGAC-uaaGCA-UGUa----guACCg---aGGaUgu-----agAAAUUUCnnnn-nnnnnnnnnnnnnnnnn-nnnnnnnnnnn
Salm.typh.-1.      GGGGCUGauucuggauu-cgaCGGGA-UUUgcgaa--AcCCa---aGGU----GCAUGCCGAGG----gGCGGUUg------gCCUCGuaaa-------aAGCCGCaaa-----------------aaaauagucgcaaacgacgaaaccuac------------------------------------------------------GCUUUAGCAG----cuuaauaa------------------------------cCUGC-uUAGAGC----------------------------------------CCUCUCU--------CCCUa--gCCUcc---------gCUCUU-AGGa--------------------------------------cGGGGauca------------AGAGAGGucaaa---cccaaAAGAGauc--------------------------------------GCGCgg---auGCCcu--GCCUGG--GGUu---gaa-GCGUuaaaa---cgaaUCAGGCua--GUCUGGUAgU---gGCGu---GUCc-GUCCGC--aGgUGC-CAGGCgaau-------------------------------------------------guaaaGAC--uGAC-uaaGCA-UGUa----guACCg---aGGaUgu-----agGAAUUUCGgac-GCGGGuucaacuCCCGC-CAGCUCCacca
Salm.typh.-2.      GGGGCUGauucuggauu-cgaCGGGA-UUUgcgaa--AcCCa---aGGU----GCAUGCCGAGG----gGCGGUUg------gCCUCGuaaa-------aAGCCGCaaa-----------------aaaauagucgcaaacgacgaaaccuac------------------------------------------------------GCUUUAGCAG----cuuaauaa------------------------------cCUGC-uUAGAGC----------------------------------------CCUCUCU--------CCCUa--gCCUcc---------gCUCUU-AGGa--------------------------------------cGGGGauca------------AGAGAGGucaaa---cccaaAAGAGauc--------------------------------------GCGCgg---auGCCcu--GCCUGG--GGUu---gaa-GCGUuaaaa---cgaaUCAGGCua--GUCUGGUAgU---gGCGu---GUCc-GUCCGC--aGgUGC-CAGGCgaau-------------------------------------------------guaaaGAC--uGAC-uaaGCA-UGUa----guACCg---aGGaUgu-----agGAAUUUCGgac-GCGGGuucaacuCCCGC-CAGCUCCacca
Salm.ente.-1.      GGGGCUGauucuggauu-cgaCGGGA-UUUgcgaa--AcCCa---aGGU----GCAUGCCGAGG----gGCGGUUg------gCCUCGuaaa-------aAGCCGCaaa-----------------aaaauagucgcaaacgacgaaaccuac------------------------------------------------------GCUUUAGCAG----cuuaauaa------------------------------cCUGC-uUAGAGC----------------------------------------CCUCUCU--------CCCUa--gCCUcc---------gCUCUU-AGGa--------------------------------------cGGGGauca------------AGAGAGGucaaa---cccaaAAGAGauc--------------------------------------GCGUgg---auGCCcu--GCCUGG--GGUu---gaa-GCGUuaaaa---cgaaUCAGGCua--GUCUGGUAgU---gGCGu---GUCc-GUCCGC--aGgUGC-CAGGCgaau-------------------------------------------------guaaaGAC--uGAC-uaaGCA-UGUa----guACCg---aGGaUgu-----agGAAUUUCGgac-GCGGGuucaacuCCCGC-CAGCUCCacca
Salm.ente.-2.      nnnnnnnnnnnnnnnnn-nnaCGGGA-UUUgcgaa--AcCCa---aGGU----GCAUGCCGAGG----gGCuGgUUg-----gCCUCGuaaa-------AAgCcGCaaa-----------------aaaauagucgcaaacgacgaaaacuac------------------------------------------------------GCUUUAGCAG----cuuaauaa------------------------------cCUGC-uUAGAGC----------------------------------------CCUCUCU--------CCCUa--gCUUcc---------gCUCUU-AAGa--------------------------------------cGGGGauca-----------aAGAGAGGucaaa---cccaaAAGAGauc--------------------------------------GCGUgg---auGCCcu--GCCUGG--GGUu---gaa-GCGUuaaaa---cgaaUCAGGCua--GUCUGGUAgU---gGCGu---GUCc-GUCCGC--aGgUGC-CAGGCgaau-------------------------------------------------guaaaGAC--aGAC-uaaGCA-UGUa----guACCg---aGGaUgu-----agGAAUUUCGgac-gcgnnnnnnnnnnnnnn-nnnnnnnnnnn
Shig.flex.         GGGGCUGauucuggauu-cgaCGGGA-UUUgcgaa--AcCCa---aGGU----GCAUGCCGAGG----gGCGGUUg------gCCUCGuaaa-------aAGCCGCaaa------------------aaauagucgcaaacgacgaaaacuac------------------------------------------------------GCUUUAGCAG----cuuaauaa------------------------------cCUGC-uUAGAGC----------------------------------------CCUCUCU--------CCCUa--gCCUcc---------gCUCUU-AGGa--------------------------------------cGGGGauca------------AGAGAGGucaaa---cccaaAAGAGauc--------------------------------------GCGUgg---aAGCCcu--GCCUGG--GGUU---gaa-GCGUuaaaa--cuuaaUCAGGCua--GUUUGUUAGU---gGCGu---GUCc-GUCCGC--aGCUGG-CAAGCgaau-------------------------------------------------guaaaGAC--uGAC-uaaGCA-UGUa----guACCg---aGGaUgu-----agGAAUUUCGgac-GCGGGuucaacuCCCGC-CAGCUCCacca
Shig.dyse.         GGGGCUGauucuggauu-cgaCGGGA-UUUgcgaa--acCCa---aGGU----GCAUGCCGAGG----gGCGGUUg------gCCUCGuaaa-------aAGCCGCaaa------------------aaauagucgcaaacgacgaaaacuac------------------------------------------------------GCUUUAGCAG----cuuaauaa------------------------------cCUGC-uUAGAGC----------------------------------------CCUCUCU--------CCCUa--gCCUcc---------gCUCUU-AGGa--------------------------------------cGGGGauca------------AGAGAGGucaaa---cccaaAAGAGauc--------------------------------------GCGUgg---aAGCCcu--GCCUGG--GGUU---gaa-GCGUuaaaa--cuuaaUCAGGCua--GUUUGUUAGU---gGCGu---GUCc-GUCCGC--aGCUGG-CAAGCgaau-------------------------------------------------guaaaGAC--uGAC-uaaGCA-UGUa----guACCg---aGGacgu-----agGAAUUCCGgac-GCGGGuucaacuCCCGC-CAGCUCCacca
Salm.para.         GGGGCUGauucuggauu-cgaCGGGA-UUUgcgaa--AcCCa---aGGU----GCAUGCCGAGG----gGCGGUUg------gCCUCGuaaa-------aAGCCGCaaa-----------------aaaauagucgcaaacgacgaaaacuac------------------------------------------------------GCUUUAGCAG----cuuaauaa------------------------------cCUGC-uUAGAGC----------------------------------------CCUCUCU--------CCCUa--gCCUcc---------gCUCUU-AGGa--------------------------------------cGGGGauca------------AGAGAGGucaaa---cccaaAAGAGauc--------------------------------------GCGCgg---auGCCcu--GCCUGG--GGUu---gag-GCGUuaaaa---cgaaUCAGGCua--GUCUGGUAgU---gGCGu---GUCc-GUCCGC--aGgUGC-CAGGCgaau-------------------------------------------------guaaaGAC--uGAC-uaaGCA-UGUa----guACCg---aGGaUgu-----agGAAUUUCGgac-GCGGGuucaacuCCCGC-CAGCUCCacca
Erwi.caro.         GGGGCUGauucuggauu-cgaCGGGA-UUugcaaa--GCCca---aGGU----GCAUGCCGAGG----gGCGGUUU------gCCUCGuaaa-------AAGCCGCaa-----------------aaaaauagucgcaaacgacgaaaacuac------------------------------------------------------GCUUUaGCAG----cuuaauaa------------------------------cCUGC-uaAGAGC----------------------------------------CCUCUCU--------CCCUa--gCCUcc---------gCUCUU-AGGa--------------------------------------cGGGGauca------------AGAGAGGucaaa---cccaaAAGAGauc--------------------------------------GCgUgg---auGCCcu--GCCUGG--GGUu---gaa-GgGCuaaau---cuaaUCAGGCua--GUUUGUUAGU---gGCGu---GUCu-GUCCGC--aGCUGG-CAAGCgaau-------------------------------------------------guaaaGAC--uGAC-uaaGCA-UGUa----guACCg---acGGUag-----aguAGUUCCGgac-GGGGGuucaaauCCCCC-CAGCUCCacca
Erwi.chry.         GGGGCUGauucuggauu-cgaCGGGA-UUCgcgaa--ACCca---aGGU----GCAUGCCGAGG----gGCGGUUg------gCCUCGuaaa-------aAGCCGCaa-----------------aaaaauagucgcaaacgacgaaaacuuc---------------------------------------------gcaccugcuGcUUUagcAG----cuuaauca-----------------------------gcCU---aaGAAaCugaaggugu-------------------------------CCUCUCU--------CCCUa--gCCUcc---------gCUCUU-AGGa--------------------------------------cGGGGauca------------AGAGAGGucaaa---ccuaaAAGAGauc--------------------------------------GCGUgg---auGCCcu--GCCUGG--GGUu---gaa-GCGUuaaau---ccaaUCAGGCua--GUUUGUUAGU---gGCGu---GUCU-GUCCGC--aGCUGG-CAAGCgaau-------------------------------------------------guaaaGAC-cAGAC-uaaGCA-UGUa----guACCg---acGGUgu-----agGAAUUUCGgac-GCGGGuucaacuCCCGC-CAGCUCCacca
Kleb.pneu.         GGGGCUGauucuggauu-cgaCGGGA-UUUgcgaa--AcCCa---aGGU----GCAUGCCGAGG----gGCGGUUg------gCCUCGuaaa-------aAGCCGCaa------------------aaaauagucgcaaacgacgaaaacuac------------------------------------------------------GCUUuaGCAG----cuuaauaa------------------------------cCUGC-ucuGAGC----------------------------------------CCUCUCU--------CCCUa--cgUUcc---------gCUCUU-AAga--------------------------------------cGGGGaucaa-----------AGAGAGGucaaa---cccaaAAGAGauc--------------------------------------GCGUgg---auGCCcu--GCCUGG--GGUu---gaa-GCGUuaaau---cuaaUCAGGCua--GUUUGUUAGU---gGCGu---GUCu-GUCCGC--aGCUGG-CAAGCgaau-------------------------------------------------guaaaGAC--uGAC-uaaGCA-UGUa----guGCCg---aGGaUgu-----agGAAUUUCGgac-GCGGGuucaacuCCCGC-CAGCUCCacca
Uncu.rca1.         nnnnnnnnnnnnnnnnn-nnaCaAGA-UUcacgaa--ACCca---aGGU----GCAUGCCGAGGu----GCGGUag------gCCUCGuuaa------caaACCGCaa------------------aaaauagucgcaaacgacgaaaacuac------------------------------------------------------GCaCUAGCAG----cuuaauac------------------------------cCUGC-uUAGaGC----------------------------------------CCUCUCU--------CCCUa--gCUUcc---------gCUCUU-AAGa--------------------------------------cGGGGauaa------------AGAGAGGucaaa---cccaaAAGAGauc--------------------------------------GCAUgg---aAGUCcu--GCCUGG--GGCU---aaa-GUGCuaaau---cuaaUCAGGCua--GUUuGUUAGU---gGCGu---GUCC-AUCCGC--aGCUGA-CcGGCgaau-------------------------------------------------guaaaGAUu-GGAC-uaaGCA-UGUa----guGCCg---acGGUgu-----agcAAUUUcGgac-gggnnnnnnnnnnnnnn-nnnnnnnnnnn
Uncu.vls1.-1.      nnnnnnnnnnnnnnnnn-nnaCAGGA-UUugcaaa--GCCcU---aGGa----GCAUGCCGAGG----gGCGGUUU------gCCUCGuaaa-------AAGCCGCaa------------------aaaauaguugcaaacgacgaaaacuac------------------------------------------------------GCaCUAGCAG----cuuaauaa------------------------------cCUGC-uUAGaGC----------------------------------------CCUCUCU--------CCCUa--gCUUcc---------GUUCUU-AAGa--------------------------------------cGGGGauca------------AGAGAGGucaaa---ccuaaAAGAACuc--------------------------------------GCGUga---auACCuu--GCCUGG--GGUu---gaa-GCGCuaaaau--cuaaUCAGGCua--GUUuGUCAGU---gGCGu---GUCC-GUUCGC--aGCUGA-CcGGCgaau-------------------------------------------------guaaaGAU--GGAC-uaaGCA-UGUa----gugCCg---AcGGUgu-----aguAAUUCUGgac-gcgnnnnnnnnnnnnnn-nnnnnnnnnnn
Uncu.vls3.         nnnnnnnnnnnnnnnnn-nnaCAGGA-UUugcaaa--GCCcU---aGGa----GCAUGCCGAGG----gGCGGUUU------gCCUCGuaaa-------AAGCCGCaa-----------------aaaaauaguugcaaacgacgaaaacuac------------------------------------------------------GCaCUAGCAG----cuuaauaa------------------------------cCUGC-uUAGaGC----------------------------------------CCUCUCU--------CCCUa--gCUUcc---------GUUCUU-AAGa--------------------------------------cGGGGauca------------AGAGAGGucaaa---ccuaaAAGAACuc--------------------------------------GCGUga---auACCuu--GCCUGG--GGUu---gaa-GCGCuaaaau--cuaaUCAGGCua--GUUuGUCAGU---gGCGu---GUCC-GUUCGC--aGCUGA-CcGGCgaau-------------------------------------------------guaaaGAU--GGAC-uaaGCA-UGUa----gugCCg---AcGGUgu-----aguAAUUCUGa-c-gcgnnnnnnnnnnnnnn-nnnnnnnnnnn
Uncu.vls4.         nnnnnnnnnnnnnnnnn-nnaCAGGA-UUugcaaa--GcCCU---aGGa----GCAUGCCGAGG----gGCGGUUU------gCCUCGuaaa-------AAGCCGCaa-----------------aaaaauaguugcaaacgacgaaaacuac------------------------------------------------------GCaCUAGCAG----cuuaauaa------------------------------cCUGC-uUAGaGC----------------------------------------CCUCUCU--------CCCUa--gCUUcc---------GUUCUU-AAGa--------------------------------------cGGGGauca------------AGAGAGGucaaa---ccuaaAAGAACuc--------------------------------------GCGUga---auACCuu--GCCUGG--GGUu---gaa-GCGCuaaaau--cuaaUCAGGCua--GUuuGUcaGU---gGCGu---GUCc-CUCCGC--aGCguG-U-uGCgaau-------------------------------------------------guaaaGAG--uGAC-uaaGCA-UGUa----guaCCg---AGGaUgu-----aguAAUUUUGgac-ggggnnnnnnnnnnnnn-nnnnnnnnnnn
Uncu.ww-1.         nnnnnnnnnnnnnnnnn-nnaCGGGA-UUUgcgaa--AcCCa---aGGU----GCAUGCCGGGG----gGCGGUUg------gCCUCGuaaa-------aAGCCGCaa-----------------aaaaauagucgcaaacgacgaaaacuac------------------------------------------------------GCUUUaGCAG----cuuaauaa------------------------------cCUGC-uaAGAGC----------------------------------------CCUCUCU--------CCCUa--gCUUcc---------gCUCUU-AAGa--------------------------------------cGGGGauaa------------AGAGAGGucaaa---cccaaAAGAGauc--------------------------------------GCGCgg---auGUCcu--GCCUGG--GGCu---gaa-GUGCuaaaa---cuaaUCAGGCua--GUuCGUUAGU---gGCGu---GUCu-G-CCGC--aGCUGG-CGuGCgaau-------------------------------------------------guaaaGaC--uGAC-uaaGCA-UGUa----guACCg---aGGaUgu-----agGAAUUUCGgac-gcgnnnnnnnnnnnnnn-nnnnnnnnnnn
Uncu.rca2.         nnnnnnnnnnnnnnnnn-nnaCGGGA-UUUgcgaa--AcCCa---aGGU----GCAUGCCGAGG----gGCGGaUg------gCCUCGuaaa-------aAgCCGCaa-----------------aaaaauagucucaaacgacgaaaacuac------------------------------------------------------GCUUUaGCAG----cuuaauaa------------------------------cCUGC-uaAGAGC----------------------------------------CCUCUCU--------CCCUa--gCUUcc---------gCUCUU-AAGa--------------------------------------cGGGGauaa------------AGAGAGGucaaa---cccaaAAGAGauc--------------------------------------GCGCgg---auGUCcu--GCCUGG--GGCu---gaa-GUGCuaaaa---cuaaUCAGGCua--GUuCGUUAGU---gGCGu---GUCu-GUCCGC--aGCUGG-CGuGCgaau-------------------------------------------------guaaaGAC--uGAC-uaaGCA-UGUa----guGCCg---aGGaUgu-----agGAAUUUCGgac-gcgnnnnnnnnnnnnnn-nnnnnnnnnnn
Uncu.ww-2.         nnnnnnnnnnnnnnnnn-nnaCGGGA-UUUgcgaa--AcCCa---aGGU----GCAUGCCGGGG----gGCGGUUg------gCCUCGuaaa-------aAGCCGCaa-----------------aaaaauagucgcaaacgacgaaaacuac------------------------------------------------------GCUUUaGCAG----cuuaauaa------------------------------cCUGC-uaAGAGC----------------------------------------CCUCUCU--------CCCUa--gCUUcc---------gCUCUU-AAGa--------------------------------------cGGGGauaa------------AGAGAGGucaaa---cccaaAAGAGauc--------------------------------------GCGCgg---auGUCcu--GCCUGG--GGCu---gaa-GUGCuaaaa---cuaaUCAGGCua--GUuCGUUAGU---gGCGu---GUCu-GU-CGC--aGCUGG-CGuGCgaau-------------------------------------------------guaaagAC--uGAC-uaaGCA-UGUa----guACCg---aGGaUgu-----agGAAUUUCGgac-gcgnnnnnnnnnnnnnn-nnnnnnnnnnn
Uncu.ww-3.         nnnnnnnnnnnnnnnnn-nnaCGGGA-UUCgcgaa--AcCCa---aGGU----GCAUGCCGAGG----gGCGGUUg------gCCUCGuaaa-------aAGCCGCaa-----------------aaaaauagucgcaaacgacgaaaacuac------------------------------------------------------GCUUUaGCAG----cuuaauaa------------------------------cCUGC-uaAGAGC----------------------------------------CCUCUCa--------CCCUa--gCUUcc---------gCUCUU-AAGa--------------------------------------cGGGGauaa------------aGAGAGGucaaa---cccaaAAGAGauc--------------------------------------GCGCgg---auGUCcu--GCCUGG--GGCu---gaa-GUGCuaaaa---cuaaUCAGGCua--GUuCGUUAGU---gGCGu---GUCu-GUCCGC--aGCUGG-CGuGCgaau-------------------------------------------------guaaaGAC--uGAC-uaaGCA-UGUa----guACCg---aGGaUgu-----agGAAUUUCGgac-gcgnnnnnnnnnnnnnn-nnnnnnnnnnn
Uncu.ql-1.         nnnnnnnnnnnnnnnnn-nnaCGGGA-UUUgcgaa--AcCCa---aGGU----GCAUGCCGAGG----gGCGGUUg------gCCUCGuaaa-------aAGCCGCaa------------------aaaauagucgcaaacgucgaaaacuac------------------------------------------------------GCUUUaGCAG----cuuaauaa------------------------------cCUGC-uaAGAGC----------------------------------------CCUCUCU--------CCCUa--gCUUcc---------gCUCUU-AAGa--------------------------------------cGGGGauaa------------AGAGAGGucaaa---cccaaAAGAGauc--------------------------------------GCGCgg---auGUCcu--GCCUGG--GGCu---gaa-GUGCuaaaa---cuaaUCAGGCua--GUuCGUUAGU---gGCGu---GUCu-GUCCGC--aGCUGG-CGuGCgaau-------------------------------------------------guaaaGAC--uGAC-uaaGCA-UGUa----guGCCg---aGGaUgu-----agGAAUUUCGgac-gunnnnnnnnnnnnnnn-nnnnnnnnnnn
Uncu.ww-4.         nnnnnnnnnnnnnnnnn-nnaCGGGA-UUCgcgaa--AcCCa---aGGU----GCAUGCCGAGG----gGCGGUUg------gCCUCGuaaa-------aAGCCGCaa-----------------aaaaauagucgcaaacgacggaaacuac------------------------------------------------------GCUUUaGCAG----cuuaauaa------------------------------cCUGC-uaAGAGC----------------------------------------CCUCUCU--------CCCUa--gCUUcc---------gCUCUU-AAGa--------------------------------------cGGGGauaa------------AGAGAGGucaaa---cccaaAAGAGacc--------------------------------------GCGCgg---auGUCcu--GCCUGG--GGCu---gaa-GUGCuaaaa---cuaaUCAGGCua--GUuCGUUAGU---gGCGu---GUCu-GUCCGC--aGCUGG-CGuGCgaau-------------------------------------------------guaaaGAC--uGAC-uaaGCA-UGUa----guGCCg---aGGaUgu-----agGAAUUUCGgac-gcgnnnnnnnnnnnnnn-nnnnnnnnnnn
Uncu.vls1.-2.pa.   ------------------------------------------------------------------------------------------------------------------------------------------------------------------------------------------------------------------------------------------------------------------------------------------------------------------------------------------------------------------------------------------------------------------------------------------------------------------------------------------------------------------------------------------------------nnnnnnnnnnnn--nnnnnnnAGU---gGCGu---GUCu-G-CCGC--aGCUgg-cgugcgaau-------------------------------------------------guaaaGsC--uGAC-uaagca-ugua----guggcg---aggaugu-----aggnnnnnnnnnn-nnnnnnnnnnnnnnnnn-nnnnnnnnnnn
Uncu.vls5.         nnnnnnnnnnnnnnnnn-nnaCGGGA-UUUgcgaa--AcCCa---aGGU----GCAUGCCGAGG----gGCGGUUg------gCCUCGuaaa-------aAGCCGCaa-----------------aaaaauagucgcaaacgacgaaaccuac------------------------------------------------------GCUUUAGCAG----cuuaauaa------------------------------cCUGC-uUAGAGC----------------------------------------CCUCUCU--------CCCUa--gCCUcc---------gCUCUU-AGGa--------------------------------------cGGGGauca------------AGAGAGGucaaa---cccaaAAGAGauc--------------------------------------GCGCgg---auGCCcu--GCCUGG--GGUu---gaa-GCGUuaaaa---cgaaUCAGGCua--GUCUGGUAgU---gGCGu---GUCc-GUCCGC--aGgUGC-CAGGCgaau-------------------------------------------------guaaaGAC--uGAC-uaaGCA-UGUa----guACCg---aGGaUgu-----agGAAUUUCGgac-gcgnnnnnnnnnnnnnn-nnnnnnnnnnn
Uncu.fs-1.         nnnnnnnnnnnnnnnnn-nnaCGGGA-UUUgcgaa--AcCCa---aGGU----GCAUGCCGAGG----gGCGGUUg------gCCUCGuaaa-------aAGCCGCaa------------------aaaaauagucgcaaacgcgaaaccuac------------------------------------------------------GCUUUAGCAG----cuuaauaa------------------------------cCUGC-uUAGAGC----------------------------------------CCUCUCU--------CCCUa--gCCUcc---------gCUCUU-AGGa--------------------------------------cGGGGauca------------AGAGAGGucaaa---cccaaAAGAGauc--------------------------------------GCGCgg---auGCCcu--GCCUGG--GGUu---gaa-GCGUuaaaa---cgaaUCAGGCua--GUCUGGUAgU---gGCGu---GUCc-GUCCGC--aGgUGC-CAGGCgaau-------------------------------------------------guaaaGAC--uGAC-uaaGCA-UGUa----guACCg---aGGaUgu-----agGAAUUUCGgac-gcnnnnnnnnnnnnnnn-nnnnnnnnnnn
Uncu.vls6.         nnnnnnnnnnnnnnnnn-nnaCGGGA-UUUgcgaa--AcCCa---aGaU----GCAUGCCGAGG----gGCGGUUg------gCCUCGuaaa-------aAGCCGCaa-----------------aaaaauagucgcaaacgacgaaaacuac------------------------------------------------------GCUUUaGCAG----cuuaauaa------------------------------cCUGC-uaAGAGC----------------------------------------CCUCUCU--------CCCUa--gCUUcc---------gCUCUU-AAGa--------------------------------------cGGGGaucaa-----------AGAGAGGucaaa---cccaaAAGAGauc--------------------------------------GCGUgg---aGGCCcu--GCCUGG--GGUU---gaa-GCGUuaaau---cuaaUCAGGCua--GUCUGGUAgU---gGCGu---GUCu-GUCCGC--aGgUGC-CAGGCgaau-------------------------------------------------guaaaGAC--uGAC-uaaGCA-UGUa----guAcCg---aGGaUgu-----agGAAUUUCGgac-gcggnnnnnnnnnnnnn-nnnnnnnnnnn
Uncu.fs-2.         nnnnnnnnnnnnnnnnn-nnaCGGGA-UUUgcgaa--AcCCa---aGGU----GCAUGCCGAGG----gGCGGUUg------gCCUCGuaaa-------aAGCCGCaa-----------------aaaaauagucgcaaacgacgaaaacuac------------------------------------------------------GCUUUaGCAG----cuuaauaa------------------------------cCUGC-uaAGAGC----------------------------------------CCUCUCU--------CCCUa--gCUUcc---------gCUCUU-AAGa--------------------------------------cGGGGaucaa-----------AGAGAGGucaaa---cccaaAAGAGauc--------------------------------------GCGUgg---aGGCCcu--GCCUGG--GGUU---gaa-GCGUuaaau---cuaaUCAGGCua--GUCUGGUAgU---gGCGu---GUCu-GUCCGC--aGgUGC-CAGGCgaau-------------------------------------------------guaaaGAC--uGAC-uaaGCA-UGUa----guACCg---aGGaUgu-----agGAAUUUCGgac-gcgnnnnnnnnnnnnnn-nnnnnnnnnnn
Uncu.ww-5.         nnnnnnnnnnnnnnnnn-nnaCGGGA-UUUgcgaa--AcCCa---aGGU----GCAUGCCGAGG----gGCGGUUg------gCCUCGuaaa-------aAGCCGCaa-----------------aaaaauagucgcaaacgacgaaaacuac------------------------------------------------------GCUUUaGCAG----cuuaauaa------------------------------cCUGC-uaAGAGC----------------------------------------CCUCUCU--------CCCUa--gCUUcc---------gCUCUU-AAGa--------------------------------------cGGGGaucaa-----------AGAGAGGucaaa---cccaaAAGAGauc--------------------------------------GCGUgg---aGGCCcu--GCCUGG--GGUU---gaa-GCGUuaaaa---cuaaUCAGGCua--GUCUGGUAgU---gGCGu---GUCu-GUCCGC--aGgUGC-CAGGCgaau-------------------------------------------------guaaaGAC--uGAC-uaaGCA-UGUa----guGCCg---aGGaUgu-----agGAAUUUCGgac-gcggnnnnnnnnnnnnn-nnnnnnnnnnn
Uncu.ww-6.         nnnnnnnnnnnnnnnnn-nnaCGGGA-UUCgcgaa--AcCCa---aGGU----GCAUGCCGAGG----gGCGGUUg------gCCUCGuaaa-------aAGCCGCaa-----------------aaaaauagucgcaaacgacgaaaacuac------------------------------------------------------GCUUuaGCAG----cuuaauaa------------------------------cCUGC-ucuGAGC----------------------------------------CCUCUCU--------CCCUa--gCUUcc---------gCUCUU-AAGa--------------------------------------cGGGGaucaa-----------AGAGAGGucaaa---cccaaAAGAGauc--------------------------------------GCGUgg---aGGCCcu--GCCUGG--GGUU---gaa-GCGUuaaaa---cuaaUCAGGCua--GUCUGGUAgU---gGCGu---GUCu-GUCCGC--aGgUGC-CAGGCgaau-------------------------------------------------guaaaGAC--uGAC-uaaGCA-UGUa----gcGCCg---aGGaUgu-----agGAAUUUCGgac-gcggnnnnnnnnnnnnn-nnnnnnnnnnn
Uncu.vlw1.         nnnnnnnnnnnnnnnnn-nnaCGGGA-aUUgcgaa--AcCCa---aGGU----GCAUGCCGAGG----gGCGGUUg------gCCUCGuaaa-------aAGCCGCaa-----------------aaaaauagucgcaaacgacgaaaacuac------------------------------------------------------GCUUUAGCAG----cuuaauaa------------------------------cCUGC-uUAGGGC----------------------------------------CCUCUCU--------CCCUa--gCUUcc---------gCUCUU-AAGa--------------------------------------cGGGGaucaa-----------AGAGAGGucaaa---cccaaAAGAGauc--------------------------------------GCGUgg---auGCCcu--GCCUGG--GGUu---gaa-GCGUuaaaa---cuaaUCAGGCua--GUCUGGUAgU---gGCGu---GUCc-GUCCGC--aGgUGC-CAGGCgaau-------------------------------------------------guaaaGAC--aGAC-uuaGCA-UGUa----guACCg---aGGaUgu-----agGAaUUUCGgac-gcggnnnnnnnnnnnnn-nnnnnnnnnnn
Uncu.vls7.         nnnnnnnnnnnnnnnnn-nnaCGGGA-UUUgcgaa--AcCCa---aGGU----GCAUGCCGAGG----gGCGGUUg------gCCUCGuaaa-------aAGCCGCaa-----------------aaaaauagucgcaaacgacgaaaacuac------------------------------------------------------GCUUUAGCAG----cuuaauaa------------------------------cCUGC-uUAGAGC----------------------------------------CCUCUCU--------CCCUa--gCUUcc---------gCUCUU-AAGa--------------------------------------cGGGGaucaa-----------AGAGAGGucaaa---cccaaAAGAGauc--------------------------------------GCGUgg---auGCCcu--GCCUGG--GGUu---gaa-GCGUuaaaa---cgaaUCAGGCua--GUCUGGUAgU---gGCGu---GUCc-GcCCGC--aGgUGC-CAGGCgaau-------------------------------------------------guaaaGaC--aGAC-uaaGCA-UGUa----guACCg---aGGaUgu-----agGAAUUUCGgac-gcggguucannnnnnnn-nnnnnnnnnnn
Uncu.vls8.         nnnnnnnnnnnnnnnnn-nnaCGGGA-UUUgcgaa--AcCCa---aGGU----GCAUGCCGaGG----gGCGGUUg------gCCgCGuaaa-------aAGCCGCaa------------------uaaauagucgcaaacgacgaaaacuac------------------------------------------------------GCUUUAGCAG----cuuaauaa------------------------------cCUGC-uUAGAGC----------------------------------------CCUCUCU--------CCCUa--gCCUcc---------gCUCUU-AGGa--------------------------------------cGGGGauca------------AGAGAGGucaaa---cccaaAAGAGauc--------------------------------------GCGUgg---aAGCCcu--GCCUGG--GGUU---gaa-GCGUuaaaa--cuuaaUCAGGCua--GUUUGUUAGU---gGCGu---GUCc-GUCCGC--aGCUGG-CAAGCgaau-------------------------------------------------guaaaGAC--uGAC-uaaGCA-UGUa----guACCg---aGGaUgu-----agGAAUUUCGgac-gcgnnnnnnnnnnnnnn-nnnnnnnnnnn
Uncu.vls9.         nnnnnnnnnnnnnnnnn-nnaCGGGA-UUugcgaa--GCCcU---aGGa----GCAUGCCGAGG----gGCGGUUg------gCCUCGuaaa-------aAGCCGCaa-----------------aaaaauagucgcaaacgacgaaaacuac------------------------------------------------------GCUUUAGCAG----cuuaauac------------------------------cCUGC-uUAGAGC----------------------------------------CCUCUCU--------CCCUa--gCCUcc---------gCUCUU-AGGa--------------------------------------cGGGGauca------------AGAGAGGucaaa---cccaaAAGAGauc--------------------------------------GCGUgg---auGUCcu--GCCUGG--GGCu---gaa-GCGUuaaac---ucaaUCAGGCua--GUCuGUCAGU---aGCGu---GUCc-AUCCGC--aGCUGG-CcGGCgaau-------------------------------------------------guaaaGAU--uGAC-uaaGCA-UGUa----gugCCg---AcGGUgu-----aguAAUUUCGgac-ggnnnnnnnnnnnnnnn-nnnnnnnnnnn
Uncu.vlw2.         nnnnnnnnnnnnnnnnn-nnaCGGGA--Uugugaa--GCCuU---aGGa----GCAUGCCGAGG----gGCGGUUU------gCCUCGuaaa-------AAGCCGCaa-----------------aaaaauagucgcaaacgacgaaaacuac------------------------------------------------------GCaCUAGCAG----cuuaauaa------------------------------cCUGC-uUAGaGC----------------------------------------CUUCUCU--------CCCUa--gCCUcc---------GCUCUU-AGGa--------------------------------------cGGGGauca------------AGAGAAGucaaa---cccaaAAGAGCuc--------------------------------------GUGUgg---auGUCuu--gCCUGG--GGCu---gaa-GCACuaaaa---cuaaUCAGGaua--GUUuGGUAAU---gGCGu---GUCu-AUCCGC--aGUUGC-CcGGCgaau-------------------------------------------------guaaaGAUa-uGAC-uaaGCA-UGUa----gugCCg---AcGGUgu-----aguAaUUUCGgac-gcgggucnnnnnnnnnn-nnnnnnnnnnn
Uncu.ww-7.         nnnnnnnnnnnnnnnnn-nnaCGGGA-UUCgcgaa--AcCCa---aGGU----GCAUGCCGAGG----gGCGGUUg------gCCUCGuaaa-------aAGCCGCaa-----------------aaaaauagucgcaaacgacgaaaacugc------------------------------------------------------GCUUUaGCAG----cuuaauaa------------------------------cCUGC-uaAGAGC----------------------------------------CCUCUCU--------CCCUa--gCUUcc---------gCUCUU-AAGa--------------------------------------cGGGGaauaa-----------AGAGAGGucaaa---cccaaAAGAGauc--------------------------------------GCGUgg---aAGCCcu--GCCUGG--GGUU---gaa-GCGUuaaaa---cuaaUCAGGCua--GUUuGUCAGU---gGCGu----UCC--UCCGC--aGCUGG-CcAGCgaau-------------------------------------------------guaaaGAcu-GGAc-uaaGCA-UGUa----guGCCg---aGGaUgu-----agGAAUUUCGgac-gcgnnnnnnnnnnnnnn-nnnnnnnnnnn
Uncu.ww-8.         nnnnnnnnnnnnnnnnn-nnaCGGGA-UUUgcgaa--AcCCa---aGGU----GCAUGCCGAGG-----GCGGUUg------gCCUCGuaaa-------aAGCCGCaa--------------aaaaaauauagucgcaaacgacgaaaacuac------------------------------------------------------GCUUuaGCAG----cuuaauaa------------------------------cCUGC-uccGAGC----------------------------------------CCUCUCU--------CCCUa--gCUUcc---------gCUCUU-AAGa--------------------------------------cGGGGaucaa-----------AGAGAGGucaaa---cccaaAAGAGauc--------------------------------------GCGUgg---aGGCCcu--GCCUGG--GGUU---gaa-GCGUuaaau---cuaaUCAGGCua--GUCUGGUAgU---gGCGu---GUCu-GUCCGC--aGgUGC-CAGGCgaau-------------------------------------------------guaaaGAC--uGAC-uaaGCA-UGUa----guACCg---aGGaUgu-----agGAAUUUCGgac-nnnnnnnnnnnnnnnnn-nnnnnnnnnnn
Yers.ente.         GGGGCUGauucuggauu-cgaCGGGA-UUcgcgaa--aCCcaa---GGU----GCAUGCCGAGG-----UGCGGag------gCCUCGuaaa------aaaCCGCAa-----------------aaaaaauaauugcaaacgacucgcaauacgaaucugcu---------------------------------------------gcUUUAGCaG----cuuaau---------------------------------CaGC-cUAAGaaac--------------------------ugaagauuc---CCUCUCU--------CCCUa--gCCUcc---------gCUCUU-AGGa--------------------------------------cGGGGauca------------AGAGAGGucaaa---ccuaaAAGAGauc--------------------------------------GCGUgg---aCACCuu--gCCUGG--GGUG---aaa-GCGUuaaac---ccaaUCAGGaua--GUUuGUUAGU---gGCGu---GUCC-GUCCGC--aGCUAA-CcGGCgaau-------------------------------------------------guaauGACu-GGAC-uaaGCA-UGUa----guGCCg---acGGcgu-----aguAAUUUCGgac-GGGGGuucaaauCCCCC-CAGCUCCacca
Yers.pest.         GGGGCUGauucuggauu-cgaCGGGA-UUcgcgaa--ACCca---aGGU----GCAUGCCGAGG-----UGCGGUg------gCCUCGuaaa------aaACCGCAaaa-----------------aaaauaguugcaaacgacgaaaacuac------------------------------------------------------GCaCUAGCAG----cuuaauaa------------------------------cCUGC-uUAGaGC----------------------------------------CCUCUCU--------gCCUa--gCCUcc---------GCUCUU-AGGa--------------------------------------cGGGgauca------------AGAGAGGucaaa---ccuaaAAGAGCuc--------------------------------------GUGUgg---aaACCuu--gCCUGG--GGUg---gaa-GCAUuaaaa---cuaaUCAGGaua--GUUuGUCAGU---aGCGu---GUCC-AUCCGC--aGCUGG-CcGGCgaau-------------------------------------------------guaauGAUu-GGAC-uaaGCA-UGUa----guGCCg---acGGUgu-----aguAAUUUCGgac-GGGGGuucaaauCCCCC-CAGCUCCacca
Desu.vulg.         GGGGGCGca-cugguuu-cgaCGGGG-AcGuagaa--GcCGG----AGUu---GCAGGCCGAGGc-----GCCGCUg-----gCCUCGuaaaa------AGCGGCaca------------------aaaguaauugccaa---caauuacgacuac---------------------------------------------------gcUcUGGCUG----cuuaa------------------------------uugaCGGC--CAcG-----------------------------------------uCGaacCg--------GUUca-cG--------------CCUGAU-agaCc---------------------------------------GGU-----------------GagaCGuaacu------ucAUCAGGc-------------------------------------ugGCAUCUga-CGgc-----GCUCGCC---gCG--aaGGGUGCgagaa-aaacaGGUGAGC----UgGUCgaAGGuu--gcCu--gGUCG-GgGGcgaaaCCUga-GAUgAcgau----------------------------------------------------ugUaCu-CGACcuaaGCC-UGUa---gacGCU----UCG-Ugugg---aaCaUUCUCGgac-GCGGGuucgauuCCCGC-CGCCUCCacca
Desu.desu.         nnnnnnnnnnnnnnnnn-nnnnGGGG-ACGuggaa--GcCGu----AGCG---GCAGGUCGAGGc-----GCCGCUg-----gCCUCGuaaaa------AGCGGCaca------------------aaaguaauugccaacaacgauuacgacuac---------------------------------------------------gcUUaCGCUG----ccuaauaa-------------------------------CAGC--GaGG-------------------caaugaccguuuaacggucgcgcCGaUCAg--------gGcCA-uG--------------CCUGAU-aacCc-------------------------------------UGaU------------------UGG-CGacacu-------uAUCAGGc-------------------------------------ugGCGAAAAc-Cggc----uCUCGCCGG----G-guUUUUCGCgaggaguuuaCCGGCGGGa--uUGCUGCGUUGu---GcCu--gGUCA-GgGGcCaa-CAGCG-CGGUGaaaua---------------------------------------------------caUaCu-UGACcuaaACC-UGUa---gaUGCU----uCG-Ugugg---aaUGUUCUCggac-ggggnnnnnnnnnnnnn-nnnnnnnnnnn
Geob.sulf.         GGGGGUGua-cagguuu-cgaCGGGG-AUag-gaa--GCUGAu---GGUu---GCAUGCCGAGGu----CCGGGCUg-----gCCUCGuuaaaa-----AGCCCGGggc-----------------gaauuacacgccgauaauuacgacuac------------------------------------------------------gcuGUAGCAG----cuuaauaa------------------------------cCUGC--UAC-------------------------------------------CGAUCCG--------CUga---GCCUcu---------CCCAC--GGGU---------------------------------------gaAGa---------------UGGAUCGuca------uuuuaGUGGGa-------------------------------------uaGUUgcGGGG---a-----GGgUCCAC--gg--CCCCaaGGCgaaacc-uuaGUGGAaUCgc-uucCCGgaaAU----CcC---uGCCA-GGGGaG--aGUcgggCGGcuaaauca--------------------------------------------------aaUCC--UGGUauaaGCA-UGUa---gacGCCu---UCGGUgu-----augAUCUUCGgac-GCGGGuucgacuCCCGC-CACCUCCacca
Geob.meta.         GGGGGUGua-cagguuu-cgaCGGGG-AUag-gaa--ACuaAa---GGUu---GCAUGCCGAGGu----CCGGGCUg-----gCCUCGuuaaaa-----AGCCCGGggc-----------------gaauuacacgccgauaauuacgacuac------------------------------------------------------gccGUAGCAG----cuuaauac------------------------------cCUGC--UAC-------------------------------------------CGAUCCG--------Ccga---GCCUcu---------CCCAC--GGGU---------------------------------------gaaGa---------------UGGAUCGuua------uuucaGUGGGa-------------------------------------uaGUCaaGGGG---a-----GugCC--Cgcgg--CCCCaaGGCgaaacu-uuaGcaGGauCgc-uucCCGgaaAU----CcC---uGCCA-GGGGaG--aGUcgggCGGcuaaauca--------------------------------------------------aaUCC--UGGCauaaGCA-UGUa---gacGCCu---UcuGUgu-----augAUCUUCGgac-GCGGGuucgacuCCCGC-CACCUCCacca
Heli.pylo.         GGGGCUGac-uuggauuucgaCAGAu-uUCuuguc--GCACA----GAUa---GCAUGCCAAGC---------GCU-------GCUUGuaaaac-----AGCaaca--------------------aaaauaacuguaaacaacacagauuac------------------------------------------gcuccagcuuaCgCUAAAGcuG----cgugaguuaa----------------------------uCucC-uUUUGGaGc---------------------------------------UGGAcU--------GAUUA--GAAUu----------UCUAGC-GUUU--------------------------------------UAAUC----------------GcUCCA-uaa----ccuuaaGCUAGAcg------------------------------------cuUUUAAA----AGGU-----GGUUC--GCCU-----UUUAAAcuaaga-aacaaGAACUcuu-gAAACUAuCUCa-agGUUuu-aGAAAgUUGGAC-caGAGcU-AGUUUuaaggcuaa----------------------------------------aaaaccaacCAAu-UUUC-uaaGCAuUGUa---gaaGUU----UGUGUuuagggcaaGAuuUUUGgac-UGGGGuucgauuCCCCA-CAGCUCCacca
Camp.jeju.         GGGAGCGac-uuggcuu-cgaCAGGA-GUAagucu--gCUUA----GAUg---GCAUGUCGCUU-------UGGGC------aAAGCGuaaaaa-----GCCCAaaua------------------aaauuaaacgcaaacaacguuaaauuc------------------------------------------gcuccugcuuacgcUaaAGCUG----cguaaguu-------------------------------CAGU--UgaGc-----------------------------------------CUGAAAu--------UUAAG---UcAu----------ACUAUC-UaG---------------------------------------CUUAA----------------uUUUCGGuca-----uuuuuGAUAGUgu-------------------------------------aGCCUUGc---GUuU-----GACaa--GcGU-----UGAGGUgaaau------aaaGUCuua--GCCUUGCUUUu-gaGUUuu-gGaAG-AUGAGC---GAAGU-AGGGUgaag--------------------------------------------------uaguCAUcuUUgC-uaaGCA-UGUa---gagGUCuuugUGGGa--------uUAUUUUUGgac-AGGGGuucgauuCCCCU-CGCUUCCacca
Fuso.nucl.-1.      GGGGAUGca-aagguuu-cgaCGGGG-UUGugagg--UUAUA----GGUa---GCAAGUCAGgC------UU-GUC-------G-CUGugaga------GACuAAacacau---------------cuuuuagauggaaacaaagauuac---------------------------------------------------------GcUuUAGCUG----cuuag--------------------------------uuCAGC--UAcAcCu---------------------------uggauaacuu--aUUcUAU--------aCGGg---UuUu----------UCAaag-GuG----------------------------------------UUGacua-------------GUAuAGauca------ccauaaaUGAuuuc-----------------------------------uCUAag----------------UUUA-------------UGGgacau----uuUAGAg------GAUAGUUuUag-uuagCcc-uGUUU-AC-Ggg---aGuAA-UUAUUacgaaa----------------------------------------------uuuaauaGU--AAAC-uaaACU-UGUa---gaaGCU----UAUGGucau---uaUGAUUUCGgac-ACGAGuucgacuCUCGU-CAUCUCCacca
Fuso.nucl.-2.      GGGGAUGca-aagguuu-cgaCGGGG-UUGugagg--UUAUA----GGUa---GCAAGUCAGgU------UU-GUC-------G-CUGugaaa------GACuAAacacau---------------cguuuagauggaaacaaagaauac---------------------------------------------------------GcUuUAGCUG----cuuag--------------------------------uuCAGC--UAcAcCu---------------------------uggauaacuu--aUUcUAU--------aCGGg---UuUu----------UCAaag-GuG----------------------------------------UUGacua-------------GUAuAGauua------ccauaaaUGAuuuc-----------------------------------uCUAag----------------UUUA-------------UGGgacau----uuUAGAg------GAUAGUUuUag-uuagCcc-uGUUU-AC-Ggg---aGuAA-UUAUUacgaaa----------------------------------------------uuuaauaGU--AAAC-uaaACU-UGUa---gaaGCU----UAUGGucgu---uaUGAUUUCGgac-ACGAGuucgacuCUCGU-CAUCUCCacca
Fuso.nucl.-3.      GGGGACGca-aagguuu-cgaCGGGG-UUGugagg--UUAUA----GGUa---GCAAGUCAGgU------UU-GUC-------G-CUGugaaa------GACuAAacacau---------------cguuuagauggaaacaaagaauac---------------------------------------------------------GcUuUAGCUG----cuuag--------------------------------uuCAGC--UAcAcCu---------------------------uggauaacuu--aUUcUAU--------aCGAg---UuUu----------UCAaag-GuG----------------------------------------UUGacua-------------GUAuAGauua------ccguaaaUGAuuuc-----------------------------------uCUAag----------------UUUA-------------UGGgacau----uuUAGAg------AAUAGUUuUag-uuagCcc-uGUUU-AC-Ggg---aGuAA-UUAUUacgaaa----------------------------------------------ucuaauaGU--AAAC-uaaACU-UGUa---gaaGCU----UAUAGucau---uaUGAUUUCGgac-ACGAGuucgauuCUCGU-CGUCUCCacca
Stre.coel.         GGGGAUGau--cgguuu-cgaCAGCGgcUGucgaa--gCAGG----gGaa---gCGUGUCGAGGaa--gCGGCCaug-----aUCUCGuaaacc----acaGGCCGaa------------------aaaauaaucgccaacaccaagcgcga-----------------------------------uuccucccagcaggccuuCgC-CcUCGCUG----ccugaucu---------------------------caggUAGC--GAaGcGaGcc----------------------uccuacuaagggagUGUCAGc--------CCGGG---gcU-----------GUUCCC-Ga----------------------------------------CCCGGau--------------cCUGGCAuca------gcuaGGGGACua------------------------------------aaCCUUg-----AUCCcg--GUCAC-G-GGGU----gaaGAGGgaaac--caaaCaGUGACug--GGCCCgucGG-----aGacuuGUCC-GCgUgau--CUccg-GGGCCgagaaa-----------------------------------------------aucgaaGC--GGAC-ugcACA-CGga---gaagC-----CCUGauuc----cgCAcCGUUGgac-GCGGGuucgauuCCCGC-CAUCUCCacca
Ther.fusc.         GGGGGUGau--cgguuu-cgaCUUCG-AUGguuguu-GUAGG----gGaa---gCGGGCCGAGGaga-gCGGaCGCa-----gCCUCGuuaaaa---auGCGaCCGcg----------------aaaaaauaagugccaacucuaagcgc-----------------------------------------------acugaguucgcu-cUCGCUg----cuuga------------------------------ucgagAGU--GAcuc-----------------------------------------UGUCGGc--------CUGGG---agC-----------GUCUCC-Ga----------------------------------------CCCAGug--------------uCCGGCAucg------uugaGGAGACuu-------------------------------------gCCGGUCA---GAUCag--GCCACAG-GAUC----UGACUGGgaca---ugcuCUGUGGCug--GGCCUgucgG-----CGacuuGUCU-GCgUGag--Cgccg-GGGCCgaggaga---------------------------------------------acgcugaGC--AGAC-ugcGCC-CGga---gaagC-----CCUGCugc----agCAUCGAAGgac-GCGGGuucgauuCCCGC-CACCUCCacca
Trop.whip.         GGGGAUGac--ugguuu-cgaCGcU--GUuuguguacUCGUG----gGaa---GCGGGUCGAGGau---GCGAGGuu-----gUCUCGuuaacg----auCCUCGCaaaa------------------uauaagugccaacuuaaagcgc-----------------------------------------------acugaucuaagu-cUUGcUG----cuuaa---------------------------------gCGcU-uAGauc-----------------------------------------UGUCGGu-------aUAGGG---cUU-----------GUCUUC-GG----------------------------------------CCCUGua--------------uCCGGCAugg-----uuuuaGAGGACuu-------------------------------------gCCGcGUG--UUUGC--c-GCUUGC--GUGAA---CAC-CGGgacu---uuauuGCAAGCug--GGCCUGuUga----UUuagauUCCG-CUGcAAa--gaAuCaGGGCUgaguau--------------------------------------------aacgguuuUAG--CGGA-uacACC-CGUa---gaagU-----CACGGg---cuccauGCcG-CGgac-GGGGGuucgauuCCCCC-CAUCUCCacca
Myco.lepr.         GGGGCUGaa--agguuu-cgaCUuCGcGCAucgaa--UCAAG----gGaa---gCGUGCCGGUGca--gGCAAgaGa-----cCACCGuaagcg-----UcgUUGCagc-----------------aauauaagcgccgauucauaucagcgc-----------------------------------------------gacuauGCU-cUCGcUG----ccuaa---------------------------------gCGaU--GGcuAGUc--------------------------------------UGUCAGa--------CCGGG---aAC-----------gCCCUC-GU----------------------------------------CCCGGag--------------cCUGGCAuca------gcuaGAGGGauc------------------------------------uaCCGAUGG---GUUCg---GUCGCG--GGAC----UCGUCGGgacac---caacCGCGACug--GGAUCgUCau---ccUGgcuaGUUC-GCgUGau--caGGa-GAUCCgaguag----------------------------------------------aggcauaGC--GAAC-uacGCA-CGga---gaagC-----CUUGAggg---aaaUGCCGuAGgac-CCGGGuucgauuCCCGG-CAGCUCCacca
Myco.aviu.         GGGGCUGaa--cgguuu-cgaCUuCGcGCAucgaa--UCAAG----gGaa---gCGUGCCGGUGca--gGCAAgaGa-----cCACCGuaagcg-----UcgUUGCgac-----------------caaauaagcgccgauucacaucagcgc-----------------------------------------------gacuacGCU-cUCGcUG----ccuaa---------------------------------gCGaC--GGcuAGUc--------------------------------------UGUCAGa--------CCGGG---aaC-----------gCCCUC-Gg----------------------------------------CCCGGac--------------cCUGGCAuca------gcuaGAGGGauc------------------------------------caCCGAuGA---GUCCg---GUCGCg--GGAC----UCcUCGGgacaa---ccacaGCGACug--GGAUCgUCau---cUCGgcuaGUUC-GCgUGAc--cgGGa-GAUCCgagcag----------------------------------------------aggcauaGC--GAAC-ugcGCA-CGga---gaagC-----CUUGAggg----aaUGCCGuAGgac-CCGGGuucgauuCCCGG-CAGCUCCacca
Myco.tube.         GGGGCUGaa--cgguuu-cgaCUuCGcGCAucgaa--UCAAG----gGaa---gCGUGCCGGUGca--gGCAAgaGa-----cCACCGuaagcg-----UcgUUGCgac-----------------caaauaagcgccgauucacaucagcgc---------------------------------------------------------cUCGcUG----ccuaa---------------------------------gCGaC--GGcuaguc--------------------------------------UGUCAGa--------CCGGG---aaC-----------gCCCUC-Gg----------------------------------------CCCGGac--------------cCUGGCAuca------gcuaGAGGGauc------------------------------------caCCGAuGA---GUCCg---GUCGCg--GGAC----UCcUCGGgacaa---ccacaGCGACug--GGAUCgUCau---cUCGgcuaGUUC-GCgUGAc--cgGGa-GAUCCgagcag----------------------------------------------aggcauaGC--GAAC-ugcGCA-CGga---gaagC-----CUUGAggg----aaUGCCGuAGgac-CCGGGuucgauuCCCGG-CAGCUCCacca
Myco.bovi.         GGGGCUGaa--cgguuu-cgaCUuCGcGCAucgaa--UCAAG----gGaa---gCGUGCCGGUGca--gGCAAgaGa-----cCACCGuaagcg-----UcgUUGCgac-----------------caaauaagcgccgauucacaucagcgc-----------------------------------------------gacuacGCU-cUCGcUG----ccuaa---------------------------------gCGaC--GGcuAGUc--------------------------------------UGUCAGa--------CCGGG---aaC-----------gCCCUC-Gg----------------------------------------CCCGGac--------------cCUGGCAuca------gcuaGAGGGauc------------------------------------caCCGAuGA---GUCCg---GUCGCg--GGAC----UCcUCGGgacaa---ccacaGCGACug--GGAUCgUCau---cUCGgcuaGUUC-GCgUGAc--cgGGa-GAUCCgagcag----------------------------------------------aggcauaGC--GAAC-ugcGCA-CGga---gaagC-----CUUGAggg----aaUGCCGuAGgac-CCGGGuucgauuCCCGG-CAGCUCCacca
Myco.mari.         GGGGCUGaa--cgguuu-cgaCUuCGcGCAucgaa--UCAAG----gGaa---gCGUGCCGGUGca--gGCAagaGa-----cCACCGuaagcg-----UcgaUGCaac-----------------uagauaagcgccgauucacaucagcgc-----------------------------------------------gacuacGCU-cUCGcUG----ccuaa---------------------------------gCGaC--GGcuAGUc--------------------------------------UGUCGGa--------CCGGG---aaC-----------gCCCUC-Gc----------------------------------------CCCGGac--------------cCCGGCAuca------gcuaGAGGGauc------------------------------------aaCCGAUGA---GUUCg---GUCGCg--GGGC----UCAUCGGgacau---caacaGCGACug--GGAUCgUCau---ccUGgcuaGUUC-GCgUGac--caGGa-GAUCCgagcag----------------------------------------------agacauaGC--GGAC-ugcGCA-CGga---gaagC-----CUUGAggg----aaUGCCGuAGgac-CCGGGuucgauuCCCGG-CAGCUCCacca
Myco.smeg.         GGGGCUGaa--cgguuu-cgaCUuCGaGCAucgaa--UCcAG----gGaa---gCGUGCCGGUGca--gGCAAgaGa-----cCACCGuaagcg-----UcgUUGCaac-----------------caauuaagcgccgauuccaaucagcgc-----------------------------------------------gacuacGCC-cUCGcUG----ccuaa---------------------------------gCGaC--GGcuGGUc--------------------------------------UGUCAGa--------CCGGG---agU-----------gCCCUC-Gg----------------------------------------CCCGGau--------------cCUGGCAuca------gcuaGAGGGacc------------------------------------caCCCACGG---GUUCg---GUCGCg--GGAC----CUGUGGGgacau--caaacaGCGACug--GGAUCgUCau---cUCGgcuuGUUC-GUgUGAc--cgGGa-GAUCCgaguag----------------------------------------------agacauaGC--GAAC-ugcGCA-CGga---gaagC-----CUcGAgga----caUGCCGuAGgac-CCGGGuucaauuCCCGG-CAGCUCCacca
Cory.diph.         GGGGCUGauuuugguuu-cgaCUGCGugCAuuaa---GCUAG---gGGaa---gCGUGCCGGUGca--gGCUAgaGa-----cCACCGauuuuaagcg-UcgUAGCaaa-----------------uauauaagcgcagagaacacucagcgc-----------------------------------------------gacuacGCcC-UcGcUG----ccuaa---------------------------uuugcagCGaC-cuGcGuGUc--------------------------------------UGUCAGc--------CUAGA----UU----------cGUCUCU-GG----------------------------------------UUUAGug--------------uCUGGCAucg------auuaAGAGACuu-------------------------------------gCUGGUUU---GUUgu---GUCAaCGG-GGC----AAACUGGgacuuu--caCCGaUGACug--GGCUCaUCau---ccgGguguGUUC-GUcCaag--ccGGa-GAGCCgaguag-------------------------------------------agauccacgcGC--GAAC-ugcGCA-CGga---gaagCC----CUAGCga-----ggUGaCGUAGgac-CCGGGuucaauuCCCGG-CAGCUCCacca
Cory.glut.         GGGGCUGa-caugguuu-cgaCUACG-UUGauuua--GCCAG---gGGaa---gCGUGCCGGUGaa--gGCUGgaGa-----cCACCGuaagcg-----UcgCAGCaaa-----------------ccaauaagcgccgagaagucucagcgc-----------------------------------------------gacuacGCcC-UcGcUG----cauaa-----------------------------gcaagCGaC--cGcGuGUc--------------------------------------UGUCAGc--------CUAGG---gaa-----------GUCCCU-ga----------------------------------------CCUAGau--------------cCUGGCAucg------acuaAGGGACuu-------------------------------------gCUGUUCA---AUCgc---GUCAGCGG-GGU----UGAACAGgacuuu--uaCCGUUGACug--GGCUCaUCau---ccGGaaguGUUC-GUcCUag--ccGGa-GGGCCaagcag-------------------------------------------agaucuuugcGC--GAAC-ugcGCA-CGga---gaagCC----CUGGCga-----ggUGACGUAGgac-CCGGGuucaauuCCCGG-CAGCUCCacca
Desu.hafn.         GGGGGUGca-cuggcuu-cgaCGGGGguCGcaaaa--UCgCU----UGGC---GCAUGCCGAGGg----GUAGCUu------uCCUCGuaaaac------AGCUGCaaa-----------------acucuaguugccaacgacgac-----------------------------------------------------aacuacGCuC-ucGCcG----cuuaa------------------ggcguaagccccgaaaCuGC--uu-GuGUccgug----------------------------------CUCGcaG-------------CGUaGG-----------GUCaCUaUC-ACG-------------------------------------gaau----------------CgcCGGG-------------AGcGGC--------------------------------------------------------c-GCCuGCC-agcuc--ccguucaaccuaua--gcaGGCuGGUccuuGgaUgcGcUu----CGC----GCG---CCGUGc--uGuCccgGgaCgagaucu------------------------------------------------aacGGa--gCGC-uaaGCA-UGUa---guGCCG----GG-GAugg----agUGcCUUCGgac-GGCGGuucgauuCCGCC-CACCUCCacca
Deha.ethe.         GGGGACGcg--ugguuu-cgaCAGGGaAGGua-----GGCUa-cuGGAUU---GCaGgcUGAGGu----GCCAUCc------aCCUCAuuaaac-----aGGUGGCaaa-----------------aaacaacuggcgaacgugaac------------------------------------------------------ucgUUcuuGcuGGCUA----aauaa------------------gacagcacauccugcuUGGC-cCcaCcucAAcgggu----------------------------caggUUUG-gG--------UGccGauuAGuugag-------GUgCCg-uUUccc------------------------------------CaaCG---------------cCgaUGAG-------------gGGgAC-----------------------------------------------------------------------------gaaagaaau---aucggcugguuu--GgCUaaaUUA---gGCc-----GgC-aaaaGC---UAGcc--AGaCaagauua-----------------------------------------------aauagacg-GaUaauccugU-aGUa-----GAUCCu--gGGUCa-------cCUUUUCUGgacgGGGAGuucgaccCUCCCcCGUCUCCacca
Heli.mobi.         ggggaacg----uguuu--gcUuGGG-AuGcg----aGCC-----GGGuU---GCc-GCC-agg-----aCCgUaaaa----agggcG------------gAaGGc----------------------uuuaauugccgaagauaacuac---------------------------------------------------------gcUUUaGCUG----cuuaa-------------------------------uugCAGUc--GAA------------------------------------------CCUCUUc------------uCCUCugu----------GCUCUCgguGAGGau------------------------------------------------------guAAGGGGuca-----uuuaaGAGAGC--------------------------------------ugGCUUc-----GACCaa--UUCucGgaGGUC------cAAGCgag----auuuaUc-GAAua--GCCUgaCcaa---cCuCu---GUCU-GcCGuGc-ggaaG-gaAGGCgaaaucua----------------------------------------------aaacGaC--AGAC-uacGCu-cGUa---guGuCCUuugugGGCa--------UuUCUUcGgac-gcggnnnnnnnnnnnnn-nnnnnnnnnnn
Baci.halo.         GGGGACGuua-cggauu-cgaCAGGG-AUGgaucg-aGCUUA----AGUg---GCGAGCCGAGg-----UGGCG--------aaCUCGuuaaaa-------CGCCAucgc-----------------caauaacuggcaaagaaaacaac---------------------------------------------------aacuUCGCAUUaGCUG----ccuaa--------------------------------gcUAGC--aAAUGCGGu-------------------------------------UCCGcCc-------------U-CCAUc----------GCCCAU-GUGGuAg-------------------------------------------------------guGuCGGGacucac--uuuuaGUGGGCua------------------------------------cgCCUcUc----ACCCucc-GUCUGu--GGGU------GaAGGaagag--auuuauCAGACua--GCCuUACgga---aGCCu---GUCG-CUGGGC--ugaaGUaAcGGCgaaa----------------------------------------------ugcgaauaCAG--UGAC-uacGCU-CGUa---gaaGCU----UAAGUacuc---guUAUUUCUGgac-GUGGGuucgauuCCCAC-CGUCUCCacca
Baci.stea.         GGGGACGauc-cggauu-cgaCAGGG-GUAggucg-aGCUUA----AGCG---GCGAGCCGAGGg----gGACG--------uCCUCGuaaaaa-------CGUCaccuaa----------------agauaacuggcaaacaaaacuac---------------------------------------------------------GcuUUAGCUG----ccuaa----------------------------uugcugCAGC--UAGcuCcu--------------------------------------CCCgCcA--------------ucgcCc----------GCgUGgcG-------------------------------------------------------------uUcGaGGGgcucau--augaagCGgGCua------------------------------------cgCCCaa-----AUCCgcc-GCCUGa--GGAU------gaGGGaagag--augaacCAGGCua--GCCGccGgga---gGCCu---GUCG-GUAGGC--ggaaCggaCGGCgaa-----------------------------------------------gcgaaauaUAC--CGAC-uacGCU-CGUa---gaUGCU----UAAGUggc----gaUGCCUCUGgac-GUGGGuucgacuCCCAC-CGUCUCCacca
Baci.mega.         nnnnnnnnnnnnnnnnn-nnnnAGGG--UAguucg-aGCUUA----GGUu---GCGAGUCGAGG-----aGAUG--------gCCUCGuuaaaa-------CAUCaacgcca--------------auaauaacuggcaaaucuaacaauaacu---------------------------------------------------ucgcuuuAGCUG----cauaauaguag--------------------------cuUAGC-gU---------------------------------------------UCCUccC-------------U-CCAUc----------GCCCAU-GUGGuAg-------------------------------------------------------gGuaAGGGacucac--uuuaaGUGGGCua------------------------------------cgCCgga-----GUUCgcc-GUCUGA--GGAC------gaaGGaagag--aauaaUCAGACua--GCGAcUGgga---cGCCu---GUUG-GUAGGC--agaaCAgcUCGCgaa-u---------------------------------------------gaucaauaUGC--CAAC-uacACU-CGUa---gacGCU----UAAGUggc----caUAuUUCUggac-guggnnnnnnnnnnnnn-nnnnnnnnnnn
Baci.subt.         GGGGACGuu-acggauu-cgaCAGGG-AUGgaucg-aGCUUG----AGCu---gCGAGCCGAGA------gGCGa--------UCUCGuaaaca-------CGCacuua-----------------aauauaacuggcaaaacuaacaguuuuaaccaaaacg------------------------------------------uagcaUUaGCUG----ccuaauaa----------------------------gcgCAGC--gAGa------------------------------------------CUCUUCc-------------uGACAUu----------GCCUAU-GUGUCu--------------------------------------------------------guGAAGAGcacauc----caaGUAGGCua------------------------------------cgCUUGC-----GUUCcc--GUCUGa--GAAC------GUAAGaaga---gaugaaCAGACua--GCUCuCggaa---gGCCc---GCCc-GCAGGC--aagaaGauGAGUgaaac---------------------------------------------cauaaauaUGC--aGGC-uacGCU-CG-a---gacGCU----UAAGUuaau--cgaUGUUUCUGgac-GUGGGuucgacuCCCAC-CGUCUCCacca
Stap.sapr.         nnnnnnnnnnnnnnnnnnnnnnnnnG-GUUccaug-aGCUAG----UUaa---GCGUGUCGAAG----gGUUGU---------CUUCGucaccaacacac-ACAGUu-------------------auaauaacuggcaaagaaaacaauaauu---------------------------------------------------ucgcaGUAGcUG----cguaaua-------------------------------gCAcU-cUGCa------------------------------------------UCGCcU------------AACGGUAUc----------UCCUAU-GUACCG------------------------------------UU-------------------aAcGCGAuucaac--cuauaGUAGGAua------------------------------------cgCUAGGC----ACUgcc--GUUUGA---AGUc---uGCCUAGaaga--gauuaaUCAAACua--GCAUAAUGa----cGGau---GUCU-AUCaCC--uccCAU-UAUGCgaaa------------------------------------------------ccuauuGAU--AGAC-uacGCA-CGUa-----gaAG--ugCUAGUauc----agAACCucuggac-gcnnnnnnnnnnnnnnn-nnnnnnnnnnn
Stap.epid.         GGGGACGuucuuggauu-cgaCAGGG-GUCccccg-aGCuuA----UUaa---GCGUGUCGGAG----gGUUGG---------CUCCGucaucaacacauuUCGGUu------------------aaauauaacugacaaaucaaacaauaauu---------------------------------------------------ucgcaGUaGcUG----cguaauag------------------------------cCAcU---GCa------------------------------------------UcGCcU------------AACAGCAUc----------UCCUAC-GUGCUG------------------------------------UU-------------------aAcGCcAuucaac---ccaaGUAGGAua------------------------------------ugCUAAAC----ACUgcc--GCUUGA---AGUc---uGUUUAGaugaa-auauaaUCAAGCua--GUAUCAUGUU----GGUu---GUUU-AUUGCU--uAGCAU-GAUGCgaaaa------------------------------------------------uuaucAAU--AAAC-uacACA-CGUa-----gaAA--gaUuuGUauc----agGACCUCUGgac-GCGGGuucgaauCCCGC-CGUCUCCacca
Stap.aure.-1.      GGGGACGuucauggauu-cgaCAGGG-GUCccccg-aGCuCA----UUaa---GCGUGUCGGAG----gGUUGU---------CUUCGucaucaacacac-ACAGUuu------------------auaauaacuggcaaaucaaacaauaauu---------------------------------------------------ucgcaGUAGcUG----ccuaauc-------------------------------gCAcU-cUGCa------------------------------------------UCGCcU------------AACAGCAUu----------UCCUAU-GUGCUG------------------------------------UU-------------------aAcGCGAuucaac---cuuaAUAGGAua------------------------------------ugCUAAAC----ACUgcc--GUUUGA---AGUc---uGUUUAGaagaa-acuuaaUCAAACua--GCAUCAUGuu----GGUu---GUUU-AUCACU--uuuCAU-GAUGCgaaac------------------------------------------------cuaucGAU--AAAC-uacACA-CGUa-----gaAA--gaUGuGUauc----agGACCUUUGgac-GCGGGuucaaauCCCGC-CGUCUCCacca
Stap.aure.-2.      GGGGACGuuucuggauu-cgaCAGGG-GUCccccg-aGCuCA----UUaa---GCGUGUCGGAG----gGUUGU---------CUUCGucaucaacacac-ACAGUuu------------------auaauaacuggcaaaucaaacaauaauu---------------------------------------------------ucgcaGUAGcUG----ccuaauc-------------------------------gCAcU-cUGCa------------------------------------------UCGCcU------------AACAGCAUu----------UCCUAU-GUGCUG------------------------------------UU-------------------aAcGCGAuucaac---cuuaAUAGGAua------------------------------------ugCUAAAC----ACUgcc--GUUUGA---AGUc---uGUUUAGaagaa-acuuaaUCAAGCua--GCAUCAUGuu----GGUu---GUUU-AUCACU--uuuCAU-GAUGCgaaac------------------------------------------------uuuucGAU--AAAC-uacACA-CGUa-----gaAA--gaUGuGUauc----agGACCUCUGgac-GCGGGuucaaauCCCGC-CGUCUCCacca
Stap.aure.-3.      GGGGACGuucauggauu-cgaCAGGG-GUaccccg-aGCuCA----UUaa---GCGUGUCGGAG----gGUUGU---------CUUCGucaucaacacac-ACAGUuu------------------auaauaacuggcaaaucaaacaauaauu---------------------------------------------------ucgcaGUAGcUG----ccuaauc-------------------------------uCAcU-cUGCa------------------------------------------UCGCcU------------AACAGCAUu----------UCCUAU-AUGCUG------------------------------------UU-------------------aAcGCGAuucaac---cuuaAUAGGAua------------------------------------ugCUAAAC----ACUgcc--GUUUGA---AGUc---uGUUUAGaagaa-acuuaaUCAAGCua--GCAUCAUGuu----GGUu---GUUU-AUCACU--uuuCAU-GAUGCgaaac------------------------------------------------cuuucGAU--AAAC-uacACA-CGUa-----gaAA--gaUGuGUauc----aggACCUCUGgac-GCGGGuucaaauCCCGC-CGUCUCCacca
Stap.aure.-4.      GGGGACGuucauggauu-cgaCAGGG-GUCccccg-aGCuCA----UUaa---GCGUGUCGGAG----gGUUGg---------CUUCGucaucaacacac-aCAGUuu------------------auaauaacuggcaaaucaaacaauaauu---------------------------------------------------ucgcaGUAGcUG----ccuaauc-------------------------------gCAcU-cUGCa------------------------------------------UCGCcU------------AACAGCAUu----------UCCUAU-GUGCUG------------------------------------UU-------------------aAcGCGAuucaac---cuuaAUAGGAua------------------------------------ugCUAAAC----ACUgcc--GUUUGA---AGUc---uGUUUAGaagaa-acuuaaUCAAGCua--GCAUCAUGuu----GGUu---GUUU-AUCACU--uuuCAU-GAUGCgaaac------------------------------------------------cuuucGAU--AAAC-uacACA-CGUa-----gaAA--gaUGuGUauc----agGACCUCUGgac-GCGGGuucaaauCCCGC-CGUCUCCacca
Stap.aure.-5.      GGGGACGuucauggauu-cgaCAGGG-GUCccccg-aGCuCA----UUaa---GCGUGUCGGAG----gGUUGU---------CUUCGucaucaacacac-ACAGUuu------------------auaauaacuggcaaaucaaacaauaauu---------------------------------------------------ucgcaGUAGcUG----ccuaauc-------------------------------gCAcU-cUGCa------------------------------------------UCGCcU------------AACAGCAUu----------UCCUAU-AUGCUG------------------------------------UU-------------------aAcGCGAuucaac---cuuaAUAGGAua------------------------------------ugCUAAAC----ACUgcc--GUUUGA---AGUc---uGUUUAGaagaa-acuuaaUCAAGCua--GCAUCAUGuu----GGUu---GUUU-AUCACU--uuuCAU-GAUGCgaaac------------------------------------------------cuuucGAU--AAAC-uacACA-CGUa-----gaAA--gaUGuGUauc----agGACCUCUGgac-GCGGGuucaaauCCCGC-CGUCUCCacca
Stap.xylo.         nnnnnnnnnnnnnnnnnnnnnnnnnG-GUUccaug-aGCUAG----UUaa---GCGUGUCGGAG----gGUUGU---------CUUCGucaccaacacac-ACAGUu-------------------auaauaacuggcaaagaaaacaauaauu---------------------------------------------------ucgcaGUAGcUG----cguaaua-------------------------------gCAcU-cUGCa------------------------------------------UCGCcU------------AACGGUAUc----------UCCUAU-GUACCG------------------------------------UU-------------------aAcGCGAuucaac--cuauaGUAGGAua------------------------------------cgCUAGGC----ACUgcc--GUUUGA---AGUc---uGCCUAGaaga--gauuaaUCAAACua--GCAUAAUGa----cGGau---GUCU-AUCaCC--uccCAU-UAUGCgaaa------------------------------------------------ccuauuGAU--AGAC-uacGCA-CGUa-----gaAG--ugCUAGUauc----agAACCucuggac-gcnnnnnnnnnnnnnnn-nnnnnnnnnnn
Baci.anth.         GGGGACGuu-acggauu-cgaCAGGG-AUAguucg-aGCUUA----GGUU---GCGAGUCGAGG-----GGauCGg-------CCUCGuu------aaaaCG--UCaaagc----------------cuauaauuggcaaacaaaacaauc------------------------------------------------------uuuCUUUaGcUG----cuuaauu-------------------------------gCAcU---AAAG-----------------------------------------GUUCCU----------CCCU--CCAUc----------GUCCAU-GUGGu-------------------------------------AGGGu----------------aAGGGAC-ucaaa---cuaaGUGGACua------------------------------------cgCCggaG----UUCgcc--GUCUGa---GGA-----CaaaGGaaga--gaacaacCAGACua--GCaAcUUgga---aGCCu---GUCG-AUAGGC--cgaaGAguUcGCgaaa----------------------------------------------ugcuaauaUAU--CGAC-uacACU-CGUa---gaAGCU----UAAGUgcc----gaUAUUUUUGgac-GUGGGuucgacuCCCAC-CGUCUCCacca
List.inno.         GGGGAUGuu-acggauu-cgaCAGGG-AUAguucg-aGCUUG----AGUu---GCGAGUCGGGG-----GGauCGU-------CCUCGuu-----aucaACG--UCaaagcca-------------auaauaacuggcaaagaaaaacaaa---------------------------------------------------accuagcUuUcGCUG----ccuaauaa------------------------------gCAGU--aGcAuagcu-------------------------------------GAUCCU----------CCGU--GCAUc----------GCCCAU-GUGCu-------------------------------------ACGGu----------------aAGGGUC-ucacu---cuaaGUGGGCua------------------------------------caCUAgUUa---AuCUcc--GUCUGA--GGuU-----AAaUAGaaga--gcuuaaUCAGACua--GCugAAUGga---aGCCu---GUUA-CCGGGC--ugaUGUuUauGCgaaa----------------------------------------------ugcuaauaCGG--UGAC-uacGCU-CGUa---gauAUU----CAAGUgcc----gaUAUUUCUGgac-GUGGGuucgacuCCCAC-CAUCUCCacca
List.mono.         GGGGAUGuu-acggauu-cgaCAGGG-AUAguucg-aGCUUG----AGUu---GCGAGUCGGGG-----GGauCGU-------CCUCGuu-----aucaACG--UCaaagcca-------------auaauaacuggcaaagaaaaacaaa---------------------------------------------------accuagcUuUcGCUG----ccuaauaa------------------------------gCAGU--aGcAuagcu-------------------------------------GAUCCU----------CCGU--GCAUc----------GCCCAU-GUGCu-------------------------------------ACGGu----------------aAGGGUC-ucacu---cuaaGUGGGCua------------------------------------caCUAgUUa---AuCUcc--GUCUGG--GGuU-----AAaUAGaaga--gcuuaaUCAGACua--GCugAAUGga---aGCCu---GUUA-CCGGGC--cgaUGUuUauGCgaaa----------------------------------------------ugcuaauaCGG--UGAC-uacGCU-CGUa---gauAUU----UAAGUgcc----gaUAUUUCUGgac-GUGGGuucgacuCCCAC-CAUCUCCacca
List.gray.-1.      nnnnnnnnn-nnnnnnn-nnaCAGGG-AUAguucg-aGCUUG----AGUu---GCGAGUCGGGG-----GGauCGU-------CCUCGuu-----aucaACG--UCaaagccaa-------------uaauaacuggcaaagaaaaacaaa---------------------------------------------------accuagcUuUcGCUG----ccuaauaa------------------------------gCAGU--aGcAuagcu-------------------------------------GAUCCU----------CCGU--GCAUc----------GCCCAU-GUGCu-------------------------------------ACGGu----------------aAGGGUC-ucacu---cuaaGUGGGCua------------------------------------caCUAgUUa---AuCUcc--GUCUgA--GGuU-----AAaUAGaaga--gcuuaaUgAGACua--GCugAAUGga---aGCCu---GUUA-CCGGGC--ugaUGUuUauGCgaaa----------------------------------------------ugcuaauaCGG--UGAC-uacGCU-CGUa---gauAUU----CAAGUgcc----gaUAUUUCUGgnn-nnnnnnnnnnnnnnnnn-nnnnnnnnnnn
List.gray.-2.      nnnnnnnnn-nnnnnnn-nnaCAGGG-AUAggucg-aGCUUG----AGUu---GCGAGCCGGGg-----GGauCGg--------CCCGuc-----aucaaCG--UCaaagccaa-------------uaauaacuggcaaacaaaacaaca---------------------------------------------------auuuagcUUUcGCUG----ccuaaua-------------------------------gCAGU-cuGAAuagcu-------------------------------------GAUCCU----------CCGU--GCAUc----------ACCCAU-GUGCu-------------------------------------ACGGu----------------aAGGGUC-ucacu--uuuaaGUGGGUua------------------------------------cgCUgGcUU---auCUcc--GUCUGG--GGca----AA-CgAGaaga--gcauaaUCAGACua--GCuaGAUaga----GCCcu--GacG-CCGGGC--agacAUcUauGCgaaa----------------------------------------------uccaaauaCGG--CaaC-uacGCU-CGUa---gauGCU----CAAGUgcc----gaUAUUUCUGgnn-nnnnnnnnnnnnnnnnn-nnnnnnnnnnn
List.ivan.         nnnnnnnnn-nnnnnnn-nnaCAGGG-AUAguucg-aGCUUG----AGUu---GCGAGUCGGGG-----GGauCGU-------CCUCGuu-----auuaACG--UCaaagccaa-------------uaauaacuggcaaagaaaaacaaa---------------------------------------------------accuagcUuUcGCUG----ccuaauaa------------------------------gCAGU--aGcAuagcu-------------------------------------GAUCCU----------CCGU--GCAUc----------GCCCAU-GUGCu-------------------------------------ACGGu----------------aAGGGUC-ucacu---uuaaGUGGGCua------------------------------------caCUAAaUa---AuCUcc--GUCUGG--GGuU-----AgUUAGaaga--gcuuaaUCAGACua--GCugAAUGga---aGCCu---GUUA-CCGGGC--ugaUGUuUauGCgaaa----------------------------------------------ugcuaauaCGG--UGAC-uacGCU-CGUa---gauAUU----UAAGUgcc----gaUAUUUCUGgnn-nnnnnnnnnnnnnnnnn-nnnnnnnnnnn
List.seel.         nnnnnnnnn-nnnnnnn-nnaCAGGG-AUAguucg-aGCUUG----AGUu---GCGAGUCGGGG-----GGauCGU-------CCUCGuu-----aucaACG--UCaaagccaa-------------uaauaacuggcaaagaaaaacaaa---------------------------------------------------accuagcUuUcGCUG----ccuaauaa------------------------------gCAGU--aGcAuagcu-------------------------------------GAUCCU----------CCGU--GCAUc----------GCCCAU-GUGCu-------------------------------------ACGGa----------------aAGGGUC-ucacu---uuaaGUGGGCua------------------------------------caCUAAaUa---AuCUcc--GUCUGG--GGuU-----AgUUAGaaga--gcuuaaUCAGACua--GCugAAUGga---aGCCu---GUUA-CCGGGC--ugaUGUuUauGCgaaa----------------------------------------------uacuaauaCGG--UGAC-uacGCU-CGUa---gauAUU----UAAGUgcc----caUAUUUCUGgnn-nnnnnnnnnnnnnnnnn-nnnnnnnnnnn
List.wels.         nnnnnnnnn-nnnnnnn-nnaCAGGG-AUAguucg-aGCUUG----AGUu---GCGAGUCGGGG-----GGauCGU-------CCUCGuu-----aucaACG--UCaaagccaa-------------uaauaacuggcaaagaaaaacaaa---------------------------------------------------accuagcUuUcGCUG----ccuaauaa------------------------------gCAGU--aGcAuagcu-------------------------------------GAUCCU----------CCGU--GCAUc----------GCCCAU-GUGCu-------------------------------------ACGGu----------------aAGGGUC-ucacu---cuaaGUGGGCua------------------------------------caCUGGCUa---AuCUcc--GUCUGA--GGuU-----AGUUGGaaga--gcuuaaUCAGACua--GCugAAUGga---aGCCu---GUUA-CCGGGC--cgaUGUuUauGCgaaa----------------------------------------------ugcuaauaCGG--UGAC-uacGCU-CGUa---gauAUU----UAAGUgcc----gaUAUUUCUGgnn-nnnnnnnnnnnnnnnnn-nnnnnnnnnnn
Leuc.mese.         nnnnnnnnnnnnnnnnnnnnnnnnnU-CGUuuaga--GCAUU----GAUu---GCGUUUCGCCa-------UCCG---------GGCGcaaa-------aCGGG-cagaaa----------------uuuuaacugcaaaaaacgaaaacu---------------------------------------------------cuUuCGCuAUCGCUG----ccuaaaaa------------------------------cCAGU--GAU-GCGuA-------------------------------------GCUUAA----------UCUU--GGuU-----------gCUGGC-GuCU--------------------------------------GAGG------------------UUAGGCcauaaa-----agGCCAGauc------------------------------------guAcUGCU----uCUUac--AUCUGGaaAAGc-----AGUAuUaaa-----ucaaUCAGGUua--GUUAUAUUGaa-gaGCCuu--GaGC-UAUGGC-agaCAAU-AUAAUgaac----------------------------------------------uacaaauaAUA--GCcC-uauAAA-CGUa---gauAUU----GGUGUauc----ggGCGAuuuggac-agnnnnnnnnnnnnnnn-nnnnnnnnnnn
Leuc.pseu.         nnnnnnnnnnnnnnnnnnnnnnnnnU-CGUuuaga--GCAUU----GcCu---GCGUUUCGCCg-------UCCG---------GGCGuuaa-------aCGGG-cagaca----------------uuuuaacugcaaaaaacgaaaacu---------------------------------------------------cuUACGcaAUcGCUG----cuuaaaaa------------------------------cCAGC--aAU-aCGUA-------------------------------------GCUUAA----------UCUU--GGuU-----------UCUGAC-GuCU--------------------------------------GAGA------------------UUAGGCcauaaa-----agGUCAGAuc------------------------------------gcAuUGUU----GUUuac--AUCUGGaauGGC-----AAUGuUaaa-----uuuaUCAGAUua--GUUAAUUUGau-uaGCCgu--GUGU-UAUGGC-guaCAAA-UUGAUgaaa----------------------------------------------uucaaaugAUA--GCAC-uauAAG-CGUa---ggcGcC----AAUGUauc----ggACGGuuuggac-agnnnnnnnnnnnnnnn-nnnnnnnnnnn
Leuc.lact.         nnnnnnnnnnnnnnnnnnnnnnnnnC-UGUuuagg--GCAUG----GACU---GCGUUUCGCCa-------UCCG---------GGCGuuaaa------aCGGG-cagaaa----------------uuuuaacugcaaaaaacgaaaacu---------------------------------------------------cuuuCGCaAUCGCUG----ccuaaaaa------------------------------aCAGU--GAU-GCGuc-------------------------------------ACUUAA----------UCUU--GGua-----------gCUGGC-guCU--------------------------------------AAGG------------------UUAGGUcauaaa-----aaGUCAGauc------------------------------------gcGuCGUU----GCUuag--AUUCGGaacAGC-----GGCGuUaaa-----ugaaUCGAGUua--GUGAUUGAUgu-uaGCCuc--GUGU-UGUGGC-ggaAUCA-AUCAUgaaa----------------------------------------------cuuaaaugACA--ACAC-uauAAG-CGUa---gaGGUU----CAUGUacc----ggGCAGuuuggac-annnnnnnnnnnnnnnn-nnnnnnnnnnn
Lact.delb.         nnnnnnnnn-nnnnnnnnnnnnAAGC-ACAguucg-aGCUUG----AaUU---GCGUUUCGUAG-----GUuACGU-------CUACGuuaaa------ACGUuACaguua---------------aauauaacugcuaaaaacgaaaaca------------------------------------------------acucuUACGCuuUAGCUG----ccuaaaaa-------------------------------CAGU--UA--GCGUA-------------------------------------GAUCCU---------CUCGG---CAUc----------gCCCAU-GUGc--------------------------------------UCGAGua---------------AGGGUCucaaauu----uaGUGGGaua------------------------------------cgUUAAAC----UUUucc--GUCUGu---AAA-----GUUUAAaaga---gaucauCAGACua--GCgaUACAGaa--uGCCu---GUCA-cUcGGC-aagCUGU-AaaGCgaaac---------------------------------------------cucaaaugaGu--UGAC-uauGAA-CGU------AGaUuuu-UAAGUguc----gaUGUGUUUnnnn-nnnnnnnnnnnnnnnnn-nnnnnnnnnnn
Lact.gass.         GGGGAUGuuuugggauu-cgaCAGGC-GUAgauuc--gCGUU----gaCU---GCGAUUCGUAG-----GUcACGU-------CUACGuuaaa------ACGUcACaguua--------------aauuauaacugcaaauaacgaaaauu---------------------------------------------------cuuACGCaguAGCUg----cuuaguca--------------------------------GGC--U---GCGU--------------------------------------GAUCCa----------auGA-CGGAUu----------GCUCGU-GUCUG-------------------------------------UCug------------------cGGGUCuuaccauu---uaACGAGCua------------------------------------cgUUUAAC----UAcuua--cCUUAA----UA-----GUUAGAaauaa--gauucUUAGGuua--GUUUUGAUAGuuuaGCCcu--GUUA-UAUGGCguuUUAUC-AAAGCgaag----------------------------------------------uuuaaguaAUA--UAAC-uauGAU-CGU------AGag-guuAACGacgg----aaUACGUUUGgac-AGGGGuucaauuCCCCU-CAUCUCCacca
Lact.sake.         GGGGCCGuu-acggauu-cgaCAUGU-GUAgguga--GCGUg----aaUU---GCACUUCGUAG-----GUuACGU-------CUGCGuuaaca-----ACGUuACaguua---------------auuauaacugcaaacaauaacaauu---------------------------------------------------cuuACGCugUAGCUg----ccuaaaca------------------------------ccAGC-aUA--GCGU--------------------------------------GAUCCG-----------UCU-GACUUc----------ACCCAA-GGGUU-------------------------------------AGAuc-----------------CGGGUCucaaauu----aaUUGGGUua------------------------------------cgUUAAAU----GCcgcc--GUUUGA---aGU-----GUUUAAaaga---gauuuUCAAGCua--GUUAGUCCAuccgaGCCuc-uGUUG-AACGGCguuuUGGG-CUAGCgaauu---------------------------------------------ucuaaauaGUU--CGAC-uauGAG-UGU------AGag-auugACGUggc----aaUGCGCAUGgac-GCGGGuucaaccCCCGC-CGGCUCCacca
Lact.helv.         nnnnnnnnnnnnnnnnnnnnnnnnnC-GUAgauuc--gCAUU----GACU---GCGAUUCGUAG-----GUuACGU-------CUACGuaaaa------ACGUuACaguua---------------aauauaacugcaaauaacaaaaauu---------------------------------------------------cuuACGCAUuaGCUG----cuuaauu--------------------------------UAGC-gc-AUGCGU--------------------------------------UGCUCuu----------UGU-CGGUU----------uACUCGU-GGCUG-------------------------------------ACAc------------------uGAGUAucaacuu----uaGCGAGUua------------------------------------cgUUUAAC----UAUcuc--ACCUGa---AUA-----GUUGAAaaga--gucuuaaCAGGUua--GCUAGCUCAuccuaGCCcu--GUUA-UAUGGCguuuUGAG-CUAGUgaag----------------------------------------------uucaaguaAUA--UAAC-uauGAU-CGUa---gaGGUC----AGUGacgg----aaUGCGucuggac-agnnnnnnnnnnnnnnn-nnnnnnnnnnn
Lact.gall.         nnnnnnnnnnnnnnnnnnnnnnnnnC-GUAgauuc--gCAUU----GA-U---GCGAUUCGUAG-----GUuACGU-------CUACGuaaaa------ACGUuACaguua---------------aauauaacugcaaauaacaaaaauu---------------------------------------------------cuuACGCAUuaGCUG----cuuaauu--------------------------------UAGC-gc-AUGCGU--------------------------------------UGCUCuu----------UGU-CGGUU----------uACUCGU-GGCUG-------------------------------------ACAc------------------uGAGUAucaacuu----uaACGAGUua------------------------------------cgUUUAAC----UAccuc--ACCUGa---aUA-----GUUGAAaaga--gucuuaaCAGGUua--GCUAGCUCAuccuaGCCcu--GUUA-UAUGGCguuuUGGG-CUAGUgaag----------------------------------------------uucaaguaAUA--UAAC-uauGAU-CGUa---gaGuUC----AGUGacgg----aaUGCGuuuggac-acnnnnnnnnnnnnnnn-nnnnnnnnnnn
Lact.plan.-1.      nnnnnnnnnnnnnnnnnnnnnnnnnU-AUAguuug--agUUc----GAUu---GCGCUUCGUAu-----GuuGCGU-------cUACGuaaaa------ACGCu-Caguuua-------------aauuauaacugcaaaaaauaauaacaauu------------------------------------------------cuUACGcUuuaGCUg----ccuaaua---------------------------------AGC-gcuuAaCGUA-------------------------------------GAUCCu-----------cCC-AGGAUc----------GUCCAU-GUUCU-------------------------------------GGa-----------------ucuGGGUCcuaaauu----uaGUGGACuua-----------------------------------cgCUCaaA----GCUucc--ACCUGG---AGU-----UgcGAGaaga--gacuaaUCAGGUua--GUCAUUGCuGg-guGCCcu--gUCA-UACGGCg-uUuGCA-AUGAUgaaa----------------------------------------------uuuaaauaGUA--UGAa-uauGAG-CGUa---gauAUC----cGAggggc----aaUAUGcuuagac-gcnnnnnnnnnnnnnnn-nnnnnnnnnnn
Carn.pisc.pa.      ---------------------------------------------------------------------------------------------------------------------------------------------------------------------------------------------------------------------nnnn----nnnnnnnn------------------------------nnnnn----nnnnnnn-------------------------------------nnnnnn-----------nnC-GGCAUc----------gCCCAU-GUGCU------------------------------------cGgg-----------------uaaggguccuauaa----uaaGUGGGaua------------------------------------cgCUAAAU----UUUucc--GUCUGu---AAA-----GUUUAGaaga---gauuauCAGACua--GCgauGCAUg--auGCCu---GUUA-gGCGGC--uaAUGU-ucaGCgaaa----------------------------------------------ccuuaauaGCa--UGAC-uaugaa-cgua---gauguc----uaagugcc----gauaugcuuggac-AGGGGuucgacuCCCCU-cgucuccacca
Ente.faec.-1.      GGGGGCGuu-acggauu-cgaCAGGC-AUAguuga--GCUUG----AAUU---GCGUUUCGUAG-----GUuACGg-------CUACGuuaaa------aCGUuACaguua---------------aauauaacugcuaaaaacgaaaacaauu------------------------------------------------cuUUCGCUuuaGCUG----ccuaaaaa------------------------------cCAGC----uAGCGAA-------------------------------------GAUCCU-----------CCC-GGCAUc----------gCCCAU-GUGCU------------------------------------cGGG-----------------ucAGGGUCcuaauc----gaaGUGGGaua------------------------------------cgCUAAAU----UUUucc--GUCUGu---AAA-----AUUUAGagga---gcuuacCAGACua--GCaaUACAGa--auGCCu---GuCA-CUCGGCa-cgCUGU-AaaGCgaacc----------------------------------------------uuuaaauGAG--UGuC-uauGAA-CGUa---gaGAUU----UAAGUggc----aaUAUGUUUGgac-GCGGGuucgacuCCCGC-CGUCUCCacca
Ente.faec.-2.      GGGGACGuu-acggauu-cgaCAGGC-ACAgucga--GCUUG----AAuu---GCGUUUCGUAG-----GUuACGU-------CUACGuaaaa------ACGUuACaguua---------------aauauaacugcuaaaaacgaaaacaacu------------------------------------------------cuUACGCUuuaGCUG----ccuaaaaa-------------------------------CAGU----uAGCGUA-------------------------------------GAUCCU-----------CUC-GGCAUc----------gCCCAU-GUGCU------------------------------------cGAG-----------------uaAGGGUCcuaacu----uuaGUGGGaua------------------------------------cgUUUCAA----CUUucc--GUCUGu---AAG-----UUGAAAaaga---gaacauCAGACua--GCgaUACAGa--auGCCu---GUCA-CUCGGCa-agCUGU-AaaGUgaauc----------------------------------------------cuuaaauGAG-uUGAC-uauGAA-CGUa---gauuUU----UAAGUggc----gaUGUGUUUGgac-GCGGGuucgacuCCCGC-CGUCUCCacca
Ente.dura.         nnnnnnnnnnnnnnnnnnnnnnnnnC-ACAguucga-GCUUG----AAuu---GCGUUUCGUAG-----GUuACGU-------CUACGuuaaa------ACGUuACaguua---------------aauauaacugcuaaaaacgaaaacaacu------------------------------------------------cuUACGCUuuaGCUG----ccuaaaaa-------------------------------CAGU----uAGCGUA-------------------------------------GAUCCU-----------CUC-GGCAUc----------gCCCAU-GUGCU------------------------------------cGAG-----------------uaAGGGUCucaaau----uuaGUGGGaua------------------------------------cgUUAAAc----UUUUcc--GUCUGu--AAAG------UUUAAaaga---gaucauCAGACua--GCaaUACAGa--auGCCu---GUCA-CUCGGCa-agCUGU-AaaGCgaaac----------------------------------------------cucaaauGAG-uUGAC-uauGAA-CGUa---gauuUU----UAAGUguc----gaUGUGuuuggac-gcnnnnnnnnnnnnnnn-nnnnnnnnnnn
Oeno.oeni.         GGGGUAGuu-ccggaauucgaCAGGU-UAUuacaa--GCAUG----GAUu---GCGUU-CCCG--------uuug--------uCGGGuuaa---------agacugcagau-----------------auaacugcaaaaaauaaugaac------------------------------------------------caucuUACGCauuaGCUG----ccuaaucg-------------------------------CAGU-----aGCGUA--------------------------------------GCCcu---------------UUGUUga---------GUCCAC-GGCAA------------------------------------cau------------------ucucGGCcuaauu-------aGUGGAUua------------------------------------gcUGCUGgca-UUUAU----GUUCGGa-AUAAA---agCAGUGaaac---aagaaUCGAACua--GCUCucGAU---uuGGaa---GUUA-GCAaCC--uaAUCu-uGAGCgaa-------------------------------------------------auuacuUGU--UAAC-uaugAG-CGUa---gauAUU----UAUGUgauu---ggAUAAUUUGgac-auggguucgacucccau-CUACUCCacca
Lact.lact.-1.      nnnnnnnnnnnnnnnnnnnnnnnnnC-AUUgucgc--GCAUg----AACu---GCAACUgCUG------agGGAU---------CAGgauaauc------AUCCgcagaua---------------aauauaacugcuaaaaauaauacacaaa------------------------------------------------cuuACGCAauGGCaG----ccuaaa---------------------------------CaGC-aCcaUGCGU---------------------------------------GCCuga-----------uuuUUGCUc----------aCUGAU-GGCAA-------------------------------------uu------------------ugacGGCcuaaacu---uuuaGUCAGaua------------------------------------cgUUGU------UGUGga--GGCUUgA-CGCA-------GCAAaaga---gauuUaAAGCCccg-CAAAAUGUCG--cuGUUu---GaG--acUGGCu-uUGGCA-UUUUGuu-----------------------------------------------aaauuugagAaa--gUcC-uauGGU-UGUa---gacGUU----gAUGUagc----aaGGUGuuuggac-agnnnnnnnnnnnnnnn-nnnnnnnnnnn
Lact.lact.-2.      nnnnnnnnnnnnnnnnnnnnnnnnnC-AUUgucgc--GCAUg----AACu---GCAACUgCUG------agGGAU---------CAGgauaauc------AUCCgcagaua---------------aauauaacugcuaaaaauaauacacaaa------------------------------------------------cuuACGCAauGGCaG----ccuaaa---------------------------------CaGC-aCcaUGCGU---------------------------------------GCCuga-----------uuuUUGCUc----------UCUGAU-GGCAA-------------------------------------uu------------------ugacGGCcuaaacu---uuuaGUCAGAua------------------------------------cgUUGU------UGUGga--GGCUUgA-CGCA-------GCAAaaga---gauuUaAAGCCccg-CAAAAUGUCG--cuGUUu---GaG--acUGGCu-uUGGCA-UUUUGuu-----------------------------------------------aaauuugagAaa--gUcC-uauGGU-UGUa---gacGUU----gAUGUagc----aaGGUGuuuggac-agnnnnnnnnnnnnnnn-nnnnnnnnnnn
Lact.lact.-3.      nnnnnnnnnnnnnnnnnnnnnnnnnC-AUUgucgc--GCAUg----AACu---GCAACUgCUG------agGGAU---------CAGgauaauc------AUCCgcagaua---------------aauauaacugcuaaaaauaauacacaaa------------------------------------------------cuuACGCAauGGCaG----ccuaaa---------------------------------CaGC-aCcaUGCGU---------------------------------------GCCuga-----------uuuUUGCUc----------aCUGAU-GGCAA-------------------------------------uu------------------ugacGGCcuaaacu---uuuaGUCAGaua------------------------------------cgUUGU------UGUGga--GACUUgA-CGCA-------GCAAaaga---gauuUaAAGUCccg-CAAAAUGUCG--cuGUUu---GaG--acUGGCu-uUGGCA-UUUUGuu-----------------------------------------------aaauuugagAaa--gUcC-uauGGU-UGUa---gacGUU----gAUGUagc----aaGGUGuuuggac-agnnnnnnnnnnnnnnn-nnnnnnnnnnn
Lact.lact.-4.      nnnnnnnnnnnnnnnnnnnnnnnnnC-AUUgucgc--GCAUg----AACu---GCAACUgCUG------agGGAU---------CAGgauaauc------AUCCgcagaua---------------aauauaacugcuaaaaauaauacacaaa------------------------------------------------cuuACGCAauGGCaG----ccuaaa---------------------------------CaGC-aCcaUGCGU---------------------------------------GCCuga-----------uuuUUGCUc----------aCUGAU-GGCAA-------------------------------------cu------------------ugacGGCcuaaacu---uuuaGUCAGaua------------------------------------cgUUGU------UGUGga--GGCUUgA-CGCA-------GCAAaaga---gauuUaAAGUCccg-CAAAAUGUCG--cuGUUu---GaG--acUGGCu-uUGGCA-UUUUGuu-----------------------------------------------aaauuugagAaa--gUcC-uauGGU-UGUa---gacGUU----gAUGUagc----aaGGUGuuuggac-agnnnnnnnnnnnnnnn-nnnnnnnnnnn
Lact.garv.         nnnnnnnnnnnnnnnnnnnnnnnnnC-AUUgucgc--GCAUg----AACu---GCAACUGGUG------GUGAUG--------uCACCuaaauc------CAUCGCagaua---------------aauauaacugcuaaaaauaacacuu---------------------------------------------------cuuACGCAcucGCUG----ccuaaa------------------------------cacCAGC----uUGCGU---------------------------------------AGCUga-----------uGaUCGUUc----------gCCGAU-GGCGG-------------------------------------Cc------------------ugaAGCUcuaaacc----ucaGUCGGaua------------------------------------cgUUUU------UAUGgu--gGCUUUA-CAUA-------AAAAaagag--auuuUAAAGCaccg-CAAAUUUCUG--cuGUUu---GaG--acUGGCu-uUGGAA-AUUUGuu-----------------------------------------------aaacuagagAaa--gUcC-uauGGU-UGUa---gacGUU----gAUGUagc----aaGGUGucuggac-agnnnnnnnnnnnnnnn-nnnnnnnnnnn
Lact.plan.-2.      nnnnnnnnnnnnnnnnnnnnnnnnnU-GUUgucgc--GUAUg----AGCu---GCGAUUAGUG-------aGACG--------uCACUcuaaac------CGUCgcagaua---------------aauauaacugcuaaaaauacacaaa---------------------------------------------------cuuACGCUuuaGCUG----ccuaaa------------------------------ccaCAGC----cGGCGU---------------------------------------GCCAga-----------cuuUCGUUc----------gCCUAU-GACGA-------------------------------------uu------------------ugaUGGCcuaauu------uaGUAGGaua------------------------------------cgCUUU------UGUGgu--GUCUUg-ACAUG-------AAAGaaga---gauUaaGAGACucg-CCAGCUGACu--uuGuUc---GaGg-auUGuCu--uGUCA-GUUGGgu------------------------------------------------aaauuuagAca--gUcCcuauGAU-UGUa---gacGCU----gAUAUagc----aaGGCAuuuggac-agnnnnnnnnnnnnnnn-nnnnnnnnnnn
Pedi.pent.         GGGGUCGuu-auggauu-cgacaggu-augggucga-GCCAU----GACu---GCAUUUCGUAG-----guuACGg-------CUACGuaaa------aaCGUucaguuua----------------uuauaacugcaaaaaauaauaacaau------------------------------------------------ucuuACGcUuuaGCUG----ccuaauag-------------------------------UAGC---uuGaCGU--------------------------------------aGAUCCG------------cCUAGUCuc---------GUCCAUgGGCUAGa------------------------------------------------------uaCGGGUCcuaaaug-----aaGUGGACuug-----------------------------------cgCUUAa----GUACUg---GcCUG---GGUGC-----uUAAGaagag--gauuuaCAGaCuag-CGuuuUAAuGaa--GCCcc--gaCA-UACGGC--gUcUUA-uuuCGcgaaa---------------------------------------------uuuaaguaGUA--UGaa-uauGAA-UGUa---gauGUC----AUGGUggc----gacaugcuuggac-GCGGGuucgacuCCCGC-CGACUCCacca
Lact.raff.         nnnnnnnnnnnnnnnnnnnnnnnnnU-GUUgucgc--GUAUg----AGCu---GCGAUUAGUG-------CGGCG--------cCACUcuaaac------CGCCGcagaua---------------aauauaacugcuaaaaauacacaaa---------------------------------------------------cuuACGCAGuaGCUG----ccuaaa------------------------------ccaCAGC----CUGCGU---------------------------------------GCCuga-----------cuuUCGCUc----------gCCUAU-GGCGA-------------------------------------uu------------------ugauGGCcuaauu------uaGUAGGaua------------------------------------cgaUUU------GACGgu--GUCUUg-ACGUC-------AGAgaaga---gauUaaGAGACucg-CAAGUUAACu--uuGuUc---GaGg-auUGuCu--cGUUA-GCUUGuu------------------------------------------------aaauuuagAca--gUcCcuauGAU-UGUa---gacGCU----gAUAUagc----aaGGCAuuuggac-agnnnnnnnnnnnnnnn-nnnnnnnnnnn
Stre.equi.         GGGGUUGuu-acggauu-cgaCAGGC-AUUaugag--GCCUA----UUUu---GCAACUCAUC--------GGCA---------GAUGuaaaaa------UGCCa-guuaa----------------auauaacugcaaaaaauaacacaacu---------------------------------------------------uACGCUuuaGCUG----ccuaaaaa------------------------------cCAGC----cGGCGU--------------------------------------GACUUCU------------AUAGGAUu----------GCUUGU-GUCUUA------------------------------------Uu-------------------AGAGGUCucaaaa-----caGCAAGCua------------------------------------cgUUUAG-----GUGAuu--GUCUAG--UCAC------CUAAAaaga---gaaccCUAGACucg-CAGGCUGAu-----GGCuugaGUUA-UGUGUC----cUUG-GUCUGuu------------------------------------------------aaaguaagACA--UAACcuauGGU-UGUa---gacAAA----UAGGUuag----caGGUGUUUGgac-GUGGGuucgacuCCCAC-CAGCUCCacca
Stre.uber.         GGGGUUGuu-acggauu-cgaCAGGC-ACUaugag--GCAUG----UUUu---GCAACUCAUC--------GGCA---------GAUGuaaaaa------UGCCa-guuaa----------------auauaacugcaaaaaauacaaacucu---------------------------------------------------uACGCUuuAGCUG----ccuaaaaa------------------------------cCAGC--U--AGCGU--------------------------------------GACUUCU------------UCAaGAUu----------GCUUGU-GUCcUG------------------------------------Au-------------------AGAAGUCucaaaa----acaGCAAGCua------------------------------------cgACUAA-----aGCcuu--GUCUAg--aGCc------UUAGUaaga---gauuuaUAGACucg-CUAAGUAAu-----GGCuugaGUUA-UGUGUC----cUUA-CUUAGuc------------------------------------------------aaaacaagACA--UAACcuauGGU-UGUa---gacAAA----UAUGUugg----caGGUGUUUGgac-GUGGGuucgacuCCCAC-CAGCUCCacca
Stre.pyog.         GGGGUUGuu-acggauu-cgaCAGGC-AUUaugag--GCAUG----UUUu---GCGUCCCAUC--------GGCA---------GAUGuaaau-------UGCCa-guuaa----------------auauaacugcaaaaaauacaaacucu---------------------------------------------------uACGCUuuAGCUG----ccuaaaaa------------------------------cCAGC--U--AGCGU--------------------------------------GACUUCU------------ACAaGAUu----------GCUUGU-GUCcUG------------------------------------Uu-------------------AGAAGUCucaaaa-----uaGCAAGCua------------------------------------cgGUUAC-----GAAAuu--GUCUAg--UUUC------GUGACaaga---gauugaUAGACucg-CAAACUAAu-----GGCuugaGUUA-UGUGUC----uUUA-GUUUGuu------------------------------------------------aaaugaagACA--UAACcuauGGA-CGUa---gacAAA----UAUGUugg----caGGUGUUUGgac-GUGGGuucgacuCCCAC-CAGCUCCacca
Stre.suis.pa.      gggguuguu-acggauu-cgacaggc-auuaugag--gcuug----uucu---gcaacuCAUC--------GGCA---------GAUGuaaaaa------UGCCa-guuaa----------------auauaacugcaaaaaauacaaacacu---------------------------------------------------uACGCAuuaGCAG----cuuaauaa------------------------------cCUGC----cUGCGU--------------------------------------GACCAGU------------CGCGAAUu----------GCUUGU-GUUUGC-----------------------------------uG--------------------AUUGGUCuuaaaa-----uaGCAAGCua------------------------------------cgUUGGG-----AUUGa---GUCAGg--CAGU------UCCAAaaga---gauuuaCUGACucg-CUUGGuCUa-----GGUuugaguua-ug-AUC----gAGu-UCAAGuu-------------------------------------------------------------------------------------------------------------------------------------------------
Stre.ther.         nnnnnnnnnnnnnnnnnnnnnnnnnC-AUUaugag--GCAUA----UUCu---GCGACUCGUg------U-GGCG----------ACGuuaa--------CGCUcAguuaa----------------auauaacugcaaaaaauacuaauucu---------------------------------------------------uACGCuGuaGCAG----ccuaaaca------------------------------cCUGC----UcGCGU--------------------------------------GACUUCU------------AGCAGAUu----------GCUUAU-GUUUGU-----------------------------------uU--------------------AGAAGUUuuauug---auuuAUAAGCua------------------------------------cgUUCGA-----UCAuu---GCCUAg--cUGG------UCGAAaaga---gauuuaUAGGCucg-CAuAUAGaa-----GGCuugaGUUA-UGUGUC----gcCU-AUcUGuu-----------------------------------------------aaaugaaagACA--UAACcuauGGU-CGUa---gacGAA----UAUGUugg----caGAUGcuuggac-gunnnnnnnnnnnnnnn-nnnnnnnnnnn
Stre.gord.         GGGGUCGuu-acggauu-cgaCAGGC-AUUaugag--GCAUA----UUUu---GCGACUCAUC------U-aGCG---------GAUGuaaaa-------CGC-cAguuaa----------------auauaacugcaaaaaauaauacuucu---------------------------------------------------uACGCUUuaGCUG----ccuaaaaa------------------------------cCAGC----GGGCGU--------------------------------------GACCcGa------------UUCGGAUu----------GCUUGU-GUCUGA-----------------------------------uG--------------------aCaGGUCuuauua----uuaGCAAGCua------------------------------------cgGUAGA-----AUCuu---GUCUAg--uGAU------UUUACaaga---gauugaUAGACucg-CUUGAUUUg-----GGCuugaGUUA-UGUGUC----aAAA-UCAAGuu------------------------------------------------aaaacaauACA--UAGCcuauGGU-UGUa---gacAAA----UGUGUugg----caGAUGUUUGgac-GUGGGuucgacuCCCAC-CGGCUCCacca
Stre.miti.         GGGGUCGuu-acggauu-cgaCAGGC-AUUaugag--GCAUA----UUUu---GCGACUCGUg------U-GGCG----------ACGuaaa--------CGCUcAguuaa----------------auauaacugcaaaaaauaacacuucu---------------------------------------------------uACGCUcUaGCUG----ccuaaaaa------------------------------cCAGC---A-GGCGU--------------------------------------GACCcGa------------UUUGGAUu----------gCUCGU-GUUCAA-----------------------------------uG--------------------aCaGGUCuuauua----uuaGCGAGaua------------------------------------cgAUCAA-----GCCuu---GUCUAg--cGGC------UUGAUaaga---gauugaUAGACucg-CGGUUUCUa-----GGCuugaGUUA-UGUGUC----gAGG-GACUGuu------------------------------------------------aaacgaauACA--UAACcuauGGU-UGUa---gacAAA----UAUGUuag----caGAUGUUUGgac-GUGGGuucgacuCCCAC-CGGCUCCacca
Stre.pneu.         GGGGUCGuu-acggauu-cgaCAGGC-AUUaugag--GCAUA----UUUu---GCGACUCGUg------U-GGCG----------ACGuaaa--------CGCUcAguuaa----------------auauaacugcaaaaaauaacacuucu---------------------------------------------------uACGCUcUaGCUG----ccuaaaaa------------------------------cCAGC---A-GGCGU--------------------------------------GACCcGa------------UUUGGAUu----------gCUCGU-GUUCAA-----------------------------------uG--------------------aCaGGUCuuauua----uuaGCGAGaua------------------------------------cgAUUAA-----GCCuu---GUCUAg--cGGU------UUGAUaaga---gauugaUAGACucg-CAGUUUCUa-----GACuugaGUUA-UGUGUC----gAGG-GGCUGuu------------------------------------------------aaaauaauACA--UAACcuauGGU-UGUa---gacAAA----UAUGUugg----caGGUGUUUGgac-GUGGGuucgacuCCCAC-CGGCUCCacca
Stre.muta.         GGGGUCGuu-acggauu-cgaCAGGC-AUUaugag--ACCUA----UUUu---GCGACUCAUC------U-aGCG---------GAUGuaaaa-------CGC-cAguuaa----------------auauaacugcaaaaaauacaaauucu---------------------------------------------------uACGCAGuaGCUG----ccuaaaaa------------------------------cCAGC----CUGUGU--------------------------------------GAUCAAU------------AACAAAUu----------GCUUGU-GUUUGU------------------------------------Ug-------------------AUUGGUCuuauug----uuaACAAGCua------------------------------------cgUUAGA-----ACuGa---GUCAGg--CuGU------UCUAAaaga---guucuaCUGACucg-CAUCGUUAg-----AGUuugaGUUA-UGUAUU----gUAA-CGGUGuu------------------------------------------------aaauaaacACA--UAACcuauAGU-UGUa---gacAAA----UGGGUuag----caGAUGUUUGgac-GUGGGuucgacuCCCAC-CGGCUCCacca
Myco.capr.         GGGGAUGuc-auggauu-ugaCAGGA-UAUcuuua--GUACA---UAUaa---GCA-GUAGUGUu---gUAGACUaua----aAUACUac--------uaGGUUUA-----------------------aaaaacgcaaauaaaaacgaagaaacuuuugaaaugccagcauuuaugaugaa---uaaugcaucagcuggagcaaacuuUAuGuUUG----cuuaauaa---------------------------cuacUAGu--U-UA------------------------------------------GUUAUAGu-------auuucAcGAAuua-uagauauuUUAAGC--UUUaU---------------------------------------------------------UUAUAACcguauuacc-caaGCUUAAua--------------------------------------GAAUAUAUgA--------UUGCAA------UaaAUAUAUUUgaaa---ucuaaUUGCAAaug-AUAUUUAACc---uUuA----GUUA-AUUUuA----GUUA-AAUAUuuu-----------------------------------------------aauuagaaAAU--UAAC-uaaAC--UGUa---gaaaGUA---UGUAUua-----auAUAUCUUGgac-GCGAGuucgauuCUCGC-CAUCUCCacca
Urea.urea.         GGGGAUGuc-acgguuu-cgaCGUGA-CACauua---AUUuu---UAAUu---GCA-GUGGGGUu---aGcccCUUau----cGCUUUc---------gAGGcauUu-----------------------uaaaugcagaaaauaaaaaaucuucugaaguagaauuaaacccagcguuuau---ggcuucagcuacuaaugcaaacuacGcUUUUGcgu-acuaauuag-----------------------uuauuagUAGA--AaC-------------------------------------------GUUcauu-----------AAcAuAAUuacua----uuGGUUg--GUUuUugggc--------------------------------UUauu----------------uuacAAUaguuuuaaa-uuuaaAAUUc---------------------------------------uuAUUUGUUGuu------UaAAUU------UaAAUAGAUuuaaca-----aauAGUUaGu---uaaUuUUAAa----uUU----GUUUuAUUAGu--uaUUAAcuAcacua---------------------------------------------------uuuuuAAUa-AAAC-uaaAC--UGUa---gauAUUA---uuAAUu------auGUGUUGCGgaa-AGGGGuucgacuCCCCU-CAUCUCCacca
Spir.kunk.         GGGGAUGuu-ccgguuu-cgaCAgGA-AUGaucu---aCUAA---UAUUg---GCA-GUAGUuUa---gUAGACUaua-----AuGCUac--------uaGGUUUG----------------------aagaaacgcaagcaaaaaacaaaaagaagacaaaauugaaaugccagcuuuuau---gaugaacaaucaauuagcaguAuCaAUgUUAGcag-cuuaauucc------------------------ucgcugUUAGaggAUcGcUagcc-----------------------------------GUaAUAG-----------UAGUUAAU---------ugUAGAUU-AUUAAC------------------------------------UA-------------------UUAUcGUaucucaagu-cuuAAUCUAaua-------------------------------------GAAAUUCUAAUu------GAUAAU-----GU-UAGAAUUUUgaaa---gucuuAUUAUCua--AUUAUUUAAU----UAGuu--GUCUaAUCCUA-uuAUUAA-AUAAU-gaa----------------------------------------------aaaaagauGAUu-AGAC-uaaAC--UGUa---gaaGAUA---UUAGaau-----guUAUUUgUGgac-GCGGGuucaauuCCCGC-CAUCUCCacca
Myco.geni.         GGGGAUGuu-uuggguu-ugaCAUAA-uGcugauag-ACAAA---CAGUa---GCA-UUGGGG------UauGCCc--------CCUUaca---gcgcuaGGUucAa-----------------------uaaccgacaaagaaaauaacgaaguguugguagaaccaaauuugaucauu------aaccaacaagcaaguguuaacuuuGCuUUUG----cauaaguag--------------------------auacUAAA---GCu------------------------------------------ACAGCUG-------------GUGAAU----------aGUCAUA-GUUUGC---------------------------------------------------------UAGCUGUcaua------guuUAUGACuc---------------------------------------GAGGUUAAAuc------GUUCaA------U-UUAACCUUua-------aaaaUaGAACuug-UUGUUUCCAu----GAUu---GUUUuGUGAUC-aauUGGA-AACAAcaaga---------------------------------------------caaaaaucCACa-AAAC-uaaAA--UGUa---gaaGCUG---UUUGUug----ugucCuUUAUGgaa-ACGGGuucgauuCCCGU-CAUCUCCacca
Myco.pneu.         GGGGAUGua-gagguuu-ugaCAUAA-uGuugaaag-gAAAA---CAGUU---GCA-GUGGGG------UAUGCC---------CCUUaca---gcucuaGGUAUAa-----------------------uaaccgacaaaaauaacgacgaaguuuugguagauccaauguugaucgcu------aaccaacaagcaaguaucaacuacGCuUUcG----cuuagaac---------------------------auacUaAA---GCu------------------------------------------ACACGAA-------------UUGAAU----------cGCCAUA-GUUUGG---------------------------------------------------------UUCGUGUcaca------guuUAUGGCuc---------------------------------------GGGGUUAAcuG------GUUCAa----C---UUAAUCCUua-------aauuaUGAACuua-UCGUUUACUU----GuUu---GUCUuAUGAuC-uaAAGUA-AGCGAga------------------------------------------------cauuaaaaCAUa-AGAC-uaaAC--UGUa---gaAGCUG---UUUUacc----aaucCuUUAUGgaa-ACGGGuucgauuCCCGU-CAUCUCCacca
Myco.pulm.         GGGGAUGua-augguuu-cgaCAgGC-AUUuauaa---cCUU---AUAAu---GCA-GUAGUUU-----GGC-----------AGACUauaaugcuacuaggcGUUuc-------------------aauaaacggaacaaaaaaacaagaaa---------------------------------------------------aUgacuaUcaAGA----uuuaauga---------------------uuagucaaaaUCUa--aAccaaaA-------------------------------------uCUAGcuUuu------gcUAGcGU-uUa----------GCAgcUaGuuGCucca---------------------------------UUAuau---------------GuuCUGGauaaugga----aGcaUGCcua-agaa--------------------------------cUUaGUGAA-GUUGcu--CUAGCgu-CAAU--UUCAUgAA---------auuuaGCUAGuug-AUUUAAUAGAa---uuuu---gUUC-AAUuuu--uUCUAU-UAAAUcaac----------------------------------------------aauuaaaaAUU--GGAa-uaaAC--UGUa---gauUUAU---AAGcauugu----gAAUGUgUGgac-CCGGGuucgacuCCCGG-CAUUUCCacca
Clos.ther.         GGGGGCGua-uugguuu-cgaCGGGA-UUGuugaag-cUUG-----AGUa---gCGGGUAGAGGa----UUCUCGUug----gCCUCUuuaaaaa----ACGAGAAac-----------------uaaaauaaacgcuaacgaagauaauuac---------------------------------------------------gcuUUaGCuGCUG----ccuaauau----------------------------aggCAGC-ccGUcAG--------------------------------------ucCAGGGuucc----ugcGCCUUGG-Gua----------gcUGGCguCaUUAaguc--------------------------------AGGC------------------cUUUUGagugggaa----gGUUAaacucuagaa--------------------------------CUGUCACAG---Ug----GUAGCCUUgAg---CUGUGAUGGauu----uuaAAGGCUAC----UgaU-AUCCAaa--gcCu--gCCCA-UggGcg--uUGGGU-uGagGg-------------------------------------------------aauuuuuaaaAcaUGGGcugcACC-CGga---gaaGCUcuu-gUGGau-------aCGAUUUCGgac-AGGGGuucgacuCCCCU-CGCCUCCacca
Ther.teng.         GGGGGUGug-uugguuu-cgaCGGGG-GCGgucga--GGUAA---AAGUa---gCGAGCCGAGUu----cUCCACC------aGCUCGuaaaaaa----GGUGGAaaa-------------------aaauaaacgcagacagagaacuc-------------------------------------------------------gcUU--ACGCUG----ccuaauua---------------------------aaggCGGC-cGUccAG--------------------------------------ccugaaaugcc------CaCGUUUcaGG----------acugggcgUCa-AAaga---------------------------------GUGgG-----------------------------------------------gaa--------------------------------CUGGUUUUG-CCU------gcGCCUCgGGG--CAAAACCGGaau----aauGAGGCua-----CCgaAGCGGUua--UcCug--UCGC-UggGaGa--ACCGC-UgaGGg--------------------------------------------------aaagcuaaaAcaGCGAcugcGCU-CGga---gaaGCUU---UUACCggg-----aCGCCUUCGgac-CGGGGuucgaauCCCCG-CACCUCCacca
Clos.perf.         GGGGGCGuu-uuggcuu-cgaCGGGG-GUAa------GaUGGguuuGAUaa--GCGAGUCGAGGg--aaGCAUGgU------gCCUCGauaauaa----AgUAUGCa----------------uuaaagauaaacgcagaagauaauuu---------------------------------------------------------uGcAUuAGCAG----cuuaauuu---------------------------agcgCUGC--UcAU-Cc------------------------------------------UUC--------------CUCAAUu----------gCCCACgGUUGAGag----------------------------------ua-------------------aGGGugucauuuaaaa----GUGGGgaa--------------------------------------CCGagCCUA-GCa------aAGCUUUgaGC--UAGGaaCGGaau---uuauGAAGCU------UacCAaAGAGgaa-GUuu---GUCU-GUGgAC--gUUCUc-UGagGgaa-----------------------------------------------uuuuaaaaCACa-AGAC-uacACU-CGUa--gaaaGUC----UUAcUggu-----cUGCUUUCGgac-ACGGGuucgacaCCCGU-CGCCUCCacca
Clos.botu.         GGGGGCGca-augguuu-cgaCGGGG-GCAaauug-cUCCUG----GGaA---gCGAGUCGaAGg--gaUCCuGUa------cCUgCGuuaaaaa-----ACuGGGa--------------acuuaaauauaauugcaaacgauaauuu---------------------------------------------------------UGcAUuAGCUG----ccuaauag----------------------------uugCAGC--UcGU-CA-----------------------------------------GC----------------CUAAGag----------UCCCACgaCUUAGg-----------------------------------gucu-------------------gGCgucgaca--------GUGGGGaa--------------------------------------CCGagUCUU-ACa------aAGCUUUgaGU--AAGGaaCGGaau---uaauGAAGCUa-----CUgaaUCUAGga--GCCu---GUUU-AUCGGC-gcUUAGA-ggaGGgaa-----------------------------------------------uguaaaagGAU--AAAC-ugcACU-CGga---gaUgCC----CAGGGguu----gcUGCUUUCGgac-AGGGGuucgauuCCCCU-CGCCUCCacca
Clos.diff.         GGGG-CGua-aagguuu-cgaCGUGG-GUUugga---aCUUGa---gGCu---GCAUGUCGUGu---uaCUCUGGg-------uCACGuaaaaa------CUGGGGa--------------acuuuaaaauaaacgcagacgauaauuu---------------------------------------------------------cgcGaUAGCAG----ccuag----------------------------------UUGC--UGcU-------------------------------------------CGUCCc-----------UCUUAGUCc----------UCCUGCcGACUAA------------------------------------GAcu------------------GGACGucauuau-------GCAGGGaa---------------------------------------CUACUUUA-GGg------guGUCUCgaCU--UGAAGUAGa-uu---aauuGGGACug----gCUaaGCAUAGa---GCCu---GCCA-CUUGGC--gCUAUG-UgaGGugaga---------------------------------------------uauuagugAAGu-UGGC-uaaGCA-UGUa---gauGCa---gCAAGgaa-----aaGAUUUGCGgac-AGGGGuucgacuCCCCU-CGuCUCCacca
Clos.acet.-1.pa.   --------------------------------------------------------------------------------------------------------------------------------------------------------------------------------------------------------------------------------------------------------------------------------------------------------------------------------------------------------------------------a-----------------------------------aucu-------------------ggcgucgaga--------gcggggaa--------------------------------------aCGagCCUU-ACa------aAGCUUUgaGU--AAGGaaCGgaau---uuauGAAGCUa----cUgaaGUGAAAa---GCUu---GUUU-GUAGGC--gUUUCA-UggaGggaa----------------------------------------------uguuaaaaUAC--AAAC-ugcacu-cgga---gaugcuu---aaaugaa-----accauuuucggac-AGGGGuucgauuCCCCU-cgccuccacca
Clos.acet.-2.      GGGGGCGcuuuugguuu-cgaCGGGG-AUGauguua-CGUUU---GAGaA---gCGAGUCGaGGg-aacCUGUGGa------cCCgCGuuaaaaaa----CUAUAG-----------------------------gccuuaaaaauaaaggau---------------------------------------aacgaaaauaauuuagcuUUAGCUG----cguag---------------------------------uCAGC--UAA--------------------------------------------CGUC-----------AGc-CUGAGag----------UCCCGCgaCUCAGa-----------------------------------guCU-------------------GGCGucgaca--------GCGGGGaa--------------------------------------CCGagCCUU-ACa------aAGCUUUgaGU--AAGGaaCGGaau---uuauGAAGCUa----cUgaaGUGAAAa---GCUu---GUUU-GUAGGC--gUUUCA-UggaGggaa----------------------------------------------uguuaaaaUAC--AAAC-ugcACU-CGga---gaUgCUU---AAAUGaa-----acCAUUUUCGgac-AGGGGuucgauuCCCCU-CGCCUCCacca
Rumi.albu.         GGGGAUGu-gaagguuu-cgaCGGGG-GUCuug----UAUGgc--GAUaa---GCGAGUGGaGG-----agcCGca-------CUcUCuuaaaa-------CG--------------------------------gcguacguuuuaaaauuaggc------------------------------------ggccacgguuauuuugcaaaGGCaa----gcuaa----------------------------------ggGC--Cacc-------------------------------------------cGUUCuu-------ccUGGgaAGUa-----------CccCGG--GCUggg-----------------------------------UUAaagcg------------uuGGAUaa-------------UUGuuGac------------------------cuuc------gccaGCcgAUG---CGGU------ugcccGGUCGu---UGUauGCcau---uuuaCcaaa------gCgagG--GgGc---GUG----GUCu-GUUUGC---C-CgugCggaGggaaacggcgcaag------------------------------------ccgaaaauAACc-cGAC-uagACU-CGUa---gaa-AUU--cgCGUGaa-----ugGGCUUUCGgac-AGGAGuucgauuCUCCU-CAUCUCCacca
Carb.hydr.         GGGGGCGu-aaaggguu-cgaCGGGGgaGCgaa----GGGCCga-AAGUa---gCGAGGCGAGG---acCCCAUCuua-----CCUCGuuaaaaa----uGGUGGG------------------aaaagauaacugccaacgaaaacuac---------------------------------------------------------GcuUUaGCuG----cuuaauaa-------------------------------C-GC---AGc-Ua----------------------------------------CGUCC---------UGACGGAAGCU----------uGCCCGG-GGCUUC------------------------------------UGUCG-----------------GGGCGu-gacc-----ggaCCGGGCug--------------------------------------GCUUAUGA-AGGGU------GUCCCGGCUCU--UCAUAAGCgag-auuuuaCGGGAUa----gCCCGAUUGuG----GCCu---GCCG-GUAGGC--aC-CAA-UCGGGugaa----------------------------------------------agcaaaaaUAC--CGGC-ugcCCU-CGga---gaaGCUU-ucGGUUC-------uuGUcCUCCGgacgGuGGGuucgauuCCCcC-CGCCUCCacca
support            ---------------------SSSSS-SSS-------SSSS-----SSS----SSSSSSSSSSS-----SSSSSS--------SSSSS------------SSSSSS---------------------------------------------------------------------------------------------------------S-SSSS-------------------------------------------SSSS---S--------------------------------------------SSSSSS---------SSSS-SS-SSS------------SSSSS-SSS-SS---------------SSSS------------SSSS--SSSS-----------------SSSSSS-------------SSSSS-----------------------------------------SSSS------SSS-----SSSSSS---SSS-------SSSS------------SSSSSS----SSSSSSSS------SSS----SSSS-SSSSSS-----SSS-SSSSS--------S--SSSSSS-------SSSSSSSSS---------SSSS------------SSS--SSSS----SSS-SSS-------SSS-----SSSS---------SSSSSSSS----S-SSS-------SSS-S------------
```

*Created by Jody Burks and Christian Zwieb*
