# Supplementary figures and images for: Comparative 3-D Modeling of tmRNA
Source: BMC Mol Biol. 2005 Jun 15;6:14. doi: 10.1186/1471-2199-6-14 (PMC1168896; doi:10.1186/1471-2199-6-14)

tmRNA (*Bacillus anthracis*)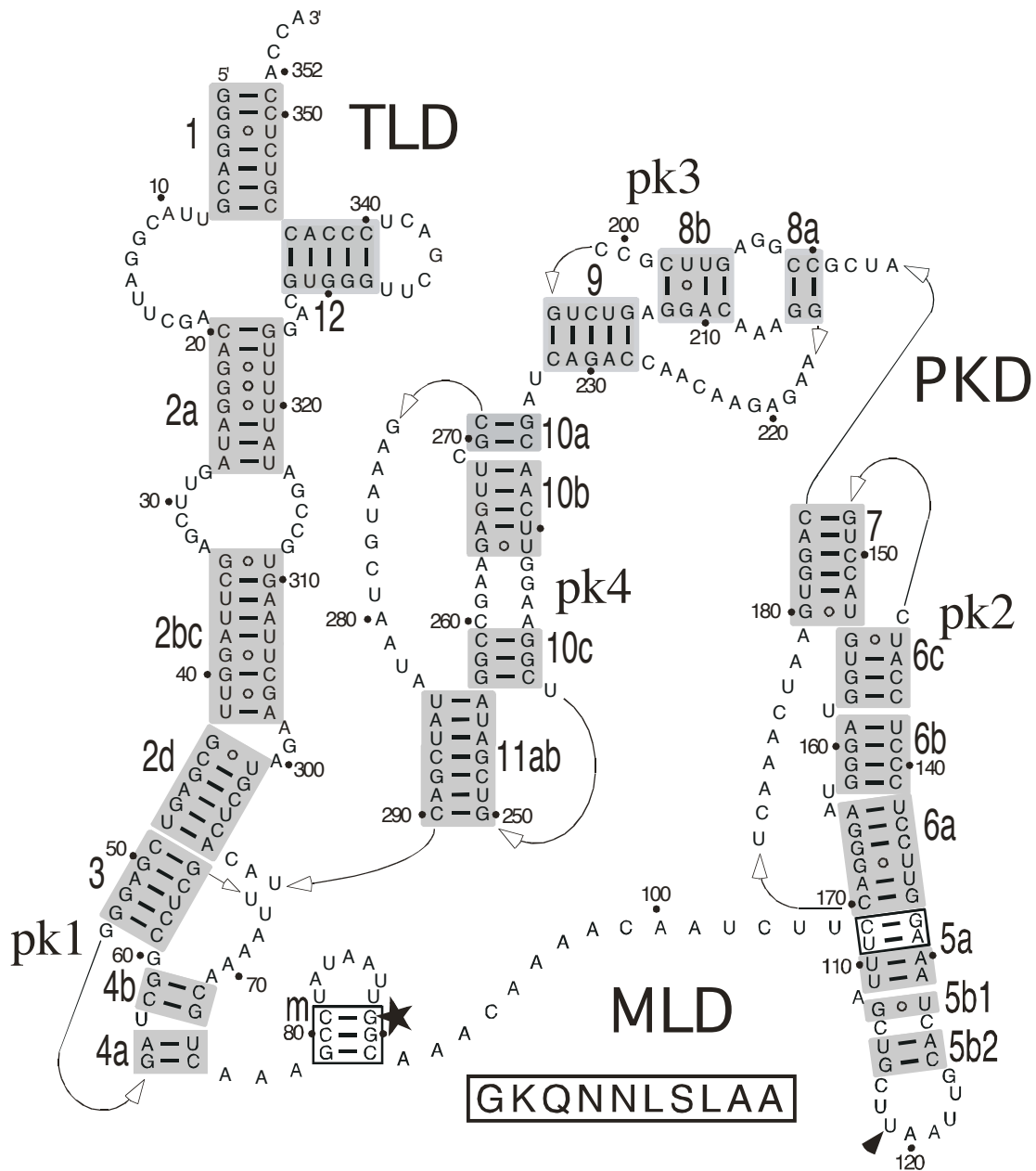

Supplement: Additional File 5 — Secondary structure of Bacillus anthracis tmRNA. Phylogenetically-supported helices are highlighted in gray and numbered from 1 to 12. The 5' and 3' ends are indicated. Arrows represent connections from 5' to 3'. Residues are numbered in increments of ten. Weakly supported regions and basepairs are show in boxes. The star labels the first nucleotide of the resume codon. The tag peptide sequence is shown below the mRNA-like region. The stop codon is indicated with a solid arrowheads. Three domains are distinguished: The tRNA-like domain (TLD), the mRNA-like domain (MLD), and the pseudoknot domain (PKD). [file 1471-2199-6-14-S5.pdf]

tmRNA (*Caulobacter crescentus*)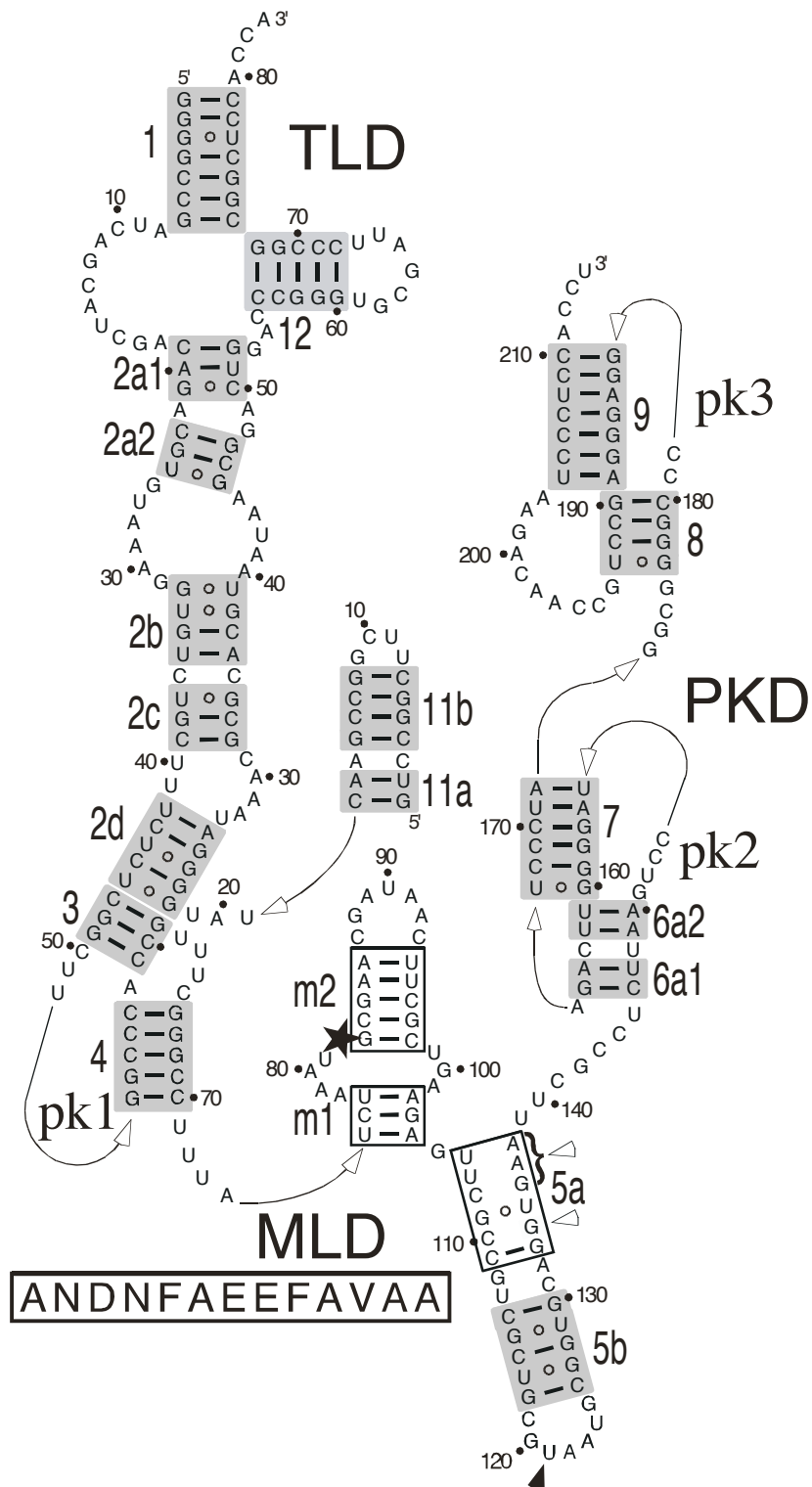

Supplement: Additional File 6 — Secondary structure of Caulobacter crescentus tmRNA. Phylogenetically-supported helices are highlighted in gray and numbered from 1 to 12. The 5' and 3' ends of both chains are indicated. Arrows represent connections from 5' to 3'. Residues are numbered in increments of ten. Weakly supported regions and basepairs are show in boxes. The star labels the first nucleotide of the resume codon. The tag peptide sequence is shown below the mRNA-like region. The stop codons are indicated with solid arrowheads. Three domains are distinguished: The tRNA-like domain (TLD), the mRNA-like domain (MLD), and the pseudoknot domain (PKD). [file 1471-2199-6-14-S6.pdf]
